# Non-equilibrium cobalt(iii) “click” capsules†
†Electronic supplementary information (ESI) available: Synthetic details, X-ray crystallography results, CV, volume calculations, scrambling and guest-binding experiments. CCDC 1014311. For ESI and crystallographic data in CIF or other electronic format see DOI: 10.1039/c4sc03036b



**DOI:** 10.1039/c4sc03036b

**Published:** 2014-10-07

**Authors:** P. R. Symmers, M. J. Burke, D. P. August, P. I. T. Thomson, G. S. Nichol, M. R. Warren, C. J. Campbell, P. J. Lusby

**Affiliations:** a EaStCHEM School of Chemistry , University of Edinburgh , The King's Buildings, David Brewster Road , Edinburgh EH9 3FJ , UK . Email: Paul.Lusby@ed.ac.uk; b Diamond Light Source Ltd , Diamond House, Harwell Science and Innovation Campus, Didcot , Oxfordshire OX11 0DE , UK

## Abstract

Cobalt(iii) tetrahedral capsules have been prepared using an assembly-followed-by-oxidation protocol from a cobalt(ii) precursor and a readily derivatizable pyridyl-triazole ligand system. Experiments designed to probe the constitutional dynamics show that these architectures are in a non-equilibrium state. A preliminary investigation into the host–guest chemistry of a water-soluble derivative shows it can bind and differentiate a range of different neutral organic molecules. The stability of this ensemble also permits the study of guest-binding at high salt concentrations.

## Abstract 2

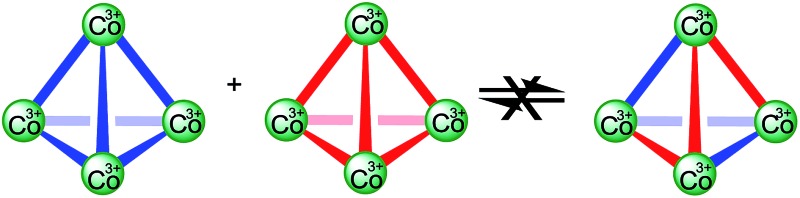
 Constitutionally non-dynamic cobalt(iii) tetrahedral capsules have been prepared using an assembly-followed-by-oxidation protocol.

## Introduction

The chemistry of molecular container species continues to thrive, not least because of applications from storage, sensing and separation, through drug delivery to catalysis.^1^ In the last twenty years, self-assembled systems have appeared, a few that rely on exclusively weak non-covalent interactions such as hydrogen bonding^2^ and many which use metal–ligand interactions.^3^ Whilst using coordination complexes as structural elements greatly increases the palate of molecular building blocks, the real advantage of these systems is that the reversibility of these interactions facilitates thermodynamic self-assembly, often producing discrete architectures in quantitative yield. However, this same facet can be viewed as a double-edged sword, with the dynamics of these systems providing a hurdle to many potential applications.^4^ A strategy that has been used to generate inert coordination based systems is to use metal–ligand interactions that are substitutionally non-labile at room temperature and only become dynamic when heated.^5^ The problem with this method is that (a) longer reaction times and templates are often required,^5*b*^ leading to lower yields and/or kinetically trapped intermediates^6^ and (b) it invariably requires the use of more expensive/more toxic third-row transition metals. An alternative way to circumvent these problems is to alter the characteristics of the transition metal center following self-assembly, most obviously through a change in the oxidation state. In this regard, cobalt would appear an ideal choice, because although Co(ii) is labile, it can be readily oxidized without a change in the coordination geometry preference to give inert Co(iii).^7^ Herein we report the synthesis of highly cationic Co(iii)_4_L_6_^12+^ tetrahedral capsules^8^ using an assembly-followed-by-oxidation protocol. These systems have the characteristics of fully covalent capsules^9^ in that they appear constitutionally non-dynamic, as evidenced by scrambling experiments. Host–guest studies with a water soluble derivative have revealed that the capsule can bind a range of neutral organic guests, and is further able to differentiate structurally similar molecules. The kinetic inertness of this system has also allowed the study of guest binding at high salt concentrations.

## Results and discussion

### Design strategy and synthesis

**Scheme 1:**
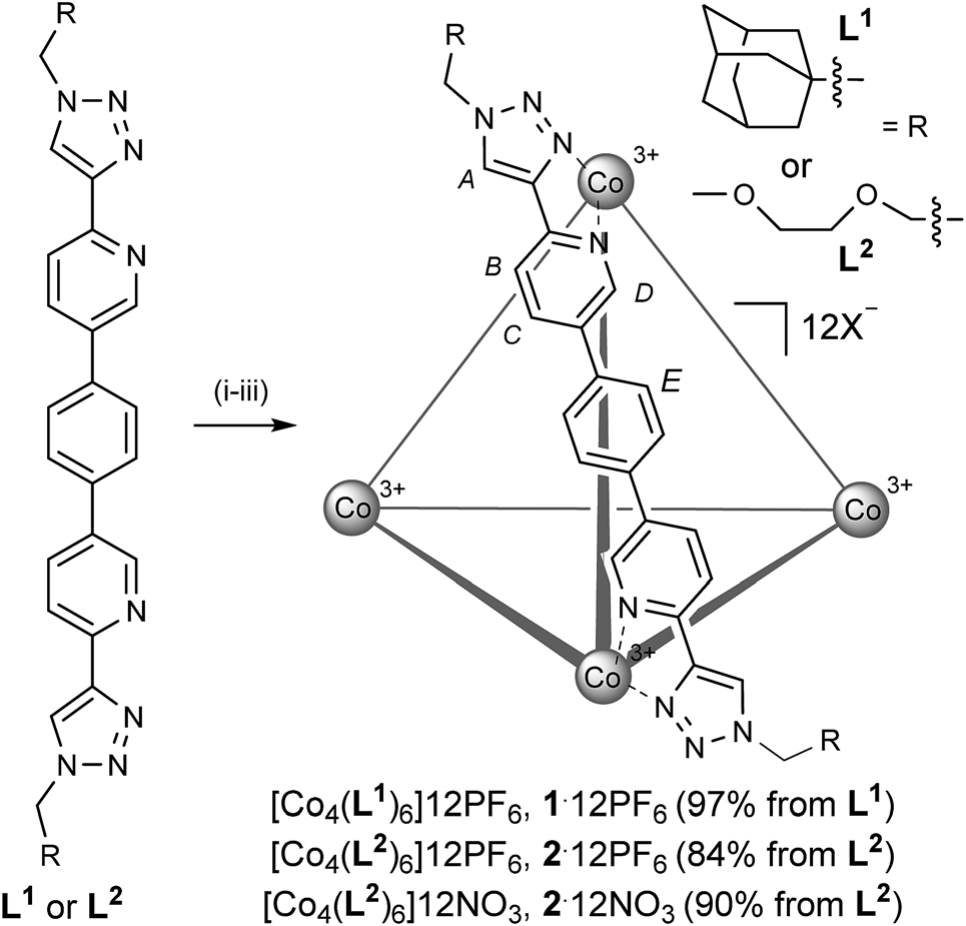
Synthesis of non-equilibrium Co(iii) capsules: (i) Co(ClO_4_)_2_·6H_2_O, CH_3_CN, 50 °C, 17 h; (ii) cerium ammonium nitrate, CH_3_CN, RT, 2.5 h; (iii) (a) NH_4_PF_6_, H_2_O, CH_3_CN, RT, 0.5 h or (b) CG-400 resin, H_2_O, CH_3_CN, RT, 2.5 h and then AgNO_3_, H_2_O, RT, 16 h.

The ligand system, **L**, that we targeted to explore the assembly-followed-by-oxidation protocol is constructed in a modular fashion (see the ESI†), using the popular copper catalyzed azide-alkyne cycloaddition (Cu-AAC) reaction (Scheme 1).^10^ Our motives for targeting this system were multiple. Firstly, the resultant *N*,*N*-donor pyridyl-triazole units are more synthetically accessible than, for example, a classic 2,2′-bipy motif.^11^ Secondly, this motif facilitates *exo*-functionalization of the capsule with different chemical groups thus facilitating various applications.^12^ Thirdly, the ligand itself is constitutionally robust, which is essential for creating non-equilibrium capsules based on substitutionally inert transition metal ions. In this regard, it can be viewed as an alternative approach to the very elegant work to recently come out of Jonathan Nitschke's laboratory.^1c,8e,g,h,14b^

Although **L^1^** showed poor solubility in all solvents, when it was reacted with Co(ClO_4_)_2_·6H_2_O in CH_3_CN, dissolution occurred over several hours at 323 K (Scheme 1, step (i)). When a small portion of this reaction was analyzed, the broadness and the position of the chemical shifts in the ^1^H NMR spectrum were strongly indicative of a Co(ii) species, while n-ESI-MS (nanoelectrospray mass spectrometry) showed predominant peaks that could be ascribed to [[Co_2_^II^(**L^1^**)_3_]*n*ClO_4_]^(4–*n*)+^ but, interestingly, no obvious indication of a Co(ii)_4_(**L^1^**)_6_ species. Subsequent slow addition of cerium ammonium nitrate (Scheme 1, step (ii)) resulted in an orange precipitate that was isolated by filtration. This intermediate mixed counteranion species was then treated with NH_4_PF_6_ (Scheme 1, step (iii) (a)) to give an orange product in 97% from **L^1^**. The ^1^H NMR spectrum of this revealed the formation of a single, highly symmetric, diamagnetic species, while analysis by n-ESI-MS showed a series of highly charged species that matched the predicted isotopic distribution for [[Co_4_(**L^1^**)_6_]*n*PF_6_]^(12–*n*)+^ (see the ESI†).

**Fig. 1:**
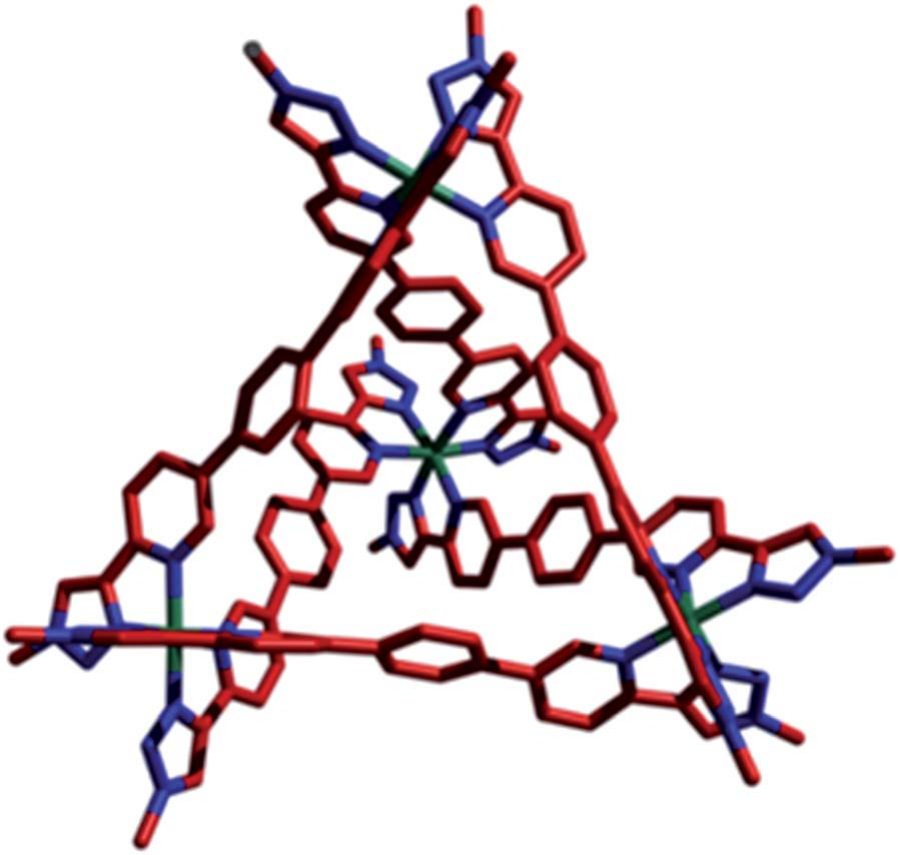
X-ray crystal structure of **1·**12PF_6_. For clarity, PF_6_ counteranions and the peripheral adamantyl groups have been removed. Color code: Co, green; C, red; N, blue.

Single crystals of [Co_4_(**L^1^**)_6_]12PF_6_, **1·**12PF_6_, suitable for XRD were grown from diisopropyl ether diffusion into saturated acetonitrile solutions. However, these crystals suffered severely from immediate and rapid solvent loss when removed from the mother liquor, such that early attempts to collect data resulted in only poorly resolved structures. Using the combination of capillary mounting in the mother liquor and a synchrotron radiation source (see the ESI†), a fully refined structure was finally obtained, which confirms a homochiral, M_4_L_6_ tetrahedral species (Fig. 1).^13^ Notably, only two PF_6_^–^ counteranions per asymmetric unit (*i.e.* per metal ion) could be identified, however, the Co–N bond lengths for the two crystallographically distinct Co environments range from 1.881(8)–2.037(8) Å (see the ESI†), completely consistent with a Co(iii) structure (as is all the other characterization data).

**2·**12PF_6_ has also been accessed using the same assembly-followed-by-oxidation and anion metathesis conditions (see the ESI†). Most probably because of the conformational flexibility of the peripheral PEG groups, we have been unable to obtain XRD-quality crystals for this compound. However, a comparison of the ^1^H NMR DOSY spectra of **1·**12PF_6_ and **2·**12PF_6_ showed very similar diffusion coefficients under the same conditions (log *D* = –9.33 and –9.34 m^2^ s^–1^, respectively), thus indicating that the assembly-followed-by-oxidation protocol with **L^2^** also gives an M_4_L_6_ species. A preliminary electrochemical investigation has also be carried out using **2·**12PF_6_ in CH_3_CN (see the ESI†), which shows a reversible reduction at –791 mV (*vs.* SCE). This single chemically-reversible reduction, which we attribute to the Co(iii)/Co(ii) couple, shows that the metal centers behave independently, and is fully chemically-reversible irrespective of scan rate, down to 50 mV s^–1^. This would indicate that the tetrahedral capsule **2** ^8+^ appears stable and does not undergo rearrangement (as perhaps could be expected with coordinatively flexile, high spin d^7^ metal vertices^14^). Electrochemical experiments also show an irreversible reduction in the region of –1300–1500 mV. These have previously been observed for pyridyl-triazole complexes, and are a result of ligand-based reduction.^15^

In order to assess whether these assemblies are in a non-equilibrium state, we have combined equimolar quantities of **1·**12PF_6_ and **2·**12PF_6_ in CD_3_CN and monitored this mixed solution as a function of time using both ^1^H NMR spectroscopy and n-ESI-MS. Similar experiments have previously been used to demonstrate that metallosupramolecular species are constitutionally dynamic; even for systems which exhibit pronounced kinetic stability, brought about by the cooperative effects of multiple metal–ligand interactions, entropy-driven scrambling of components still happens at room temperature over a few days.^16^ In contrast, we observe no ligand exchange after a week at room temperature. Only through prolonged heating of the same sample, first at 50 °C (1 week), then at 60 °C (1 week) and then finally at 70 °C, could any mixed component species be identified, but even then the ^1^H NMR spectrum remained largely unchanged and only minor peaks were observed by MS (see the ESI†). This indicates that these Co(iii) tetrahedra are constitutionally non-dynamic.

### Host–guest chemistry

Water-soluble systems have featured prominently as solution container compounds,^1b,c,d,f,g,5b,17^ principally because the hydrophobic effect is a powerful driving force for the encapsulation of a wide range of molecules. For charged metallosupramolecular capsules, dissolution in water or other polar media also results in solvation of the associated counteranions (or countercations in the case of Raymond's anionic Ga(iii) tetrahedra^1c,8b,c^), which can occupy the cavity^8d,f,g,14b,18^ and block different guests from binding. While **2·**12PF_6_ is insoluble in water, we were encouraged that the intermediate **2** ^12+^ with mixed ClO_4_–NO_3_ counteranions (*i.e.* the species obtained directly from step (ii)) is soluble in 1 : 1 CD_3_CN : D_2_O. To further increase aqueous solubility, this species was first treated with CG-400 resin and then with AgNO_3_ to give **2·**12NO_3_ (Scheme 1, step (iii) (b)). All the spectroscopic evidence (MS, ^1^H NMR, DOSY, see the ESI†) indicates that anion exchange takes place without perturbation to the tetrahedral framework, and furthermore, the resulting compound is soluble in water at 2.5 mM. It is interesting to note that the use of nitrate counteranions to water-solubilize coordination capsules has largely been limited to those systems which possess 2nd and 3rd row transition metals (most commonly Pd and Pt), probably a reflection of the softer bonding characteristics in comparison to the 1st row elements, which (in addition to nitrate-hydration) ensures outer-sphere coordination is thermodynamically preferred. Despite the oxophilic nature of Co(iii), **2·**12NO_3_ appears indefinitely stable as a 2.5 mM solution in D_2_O, further highlighting that these species exist in an out-of-equilibrium state.^19^

**Fig. 2:**
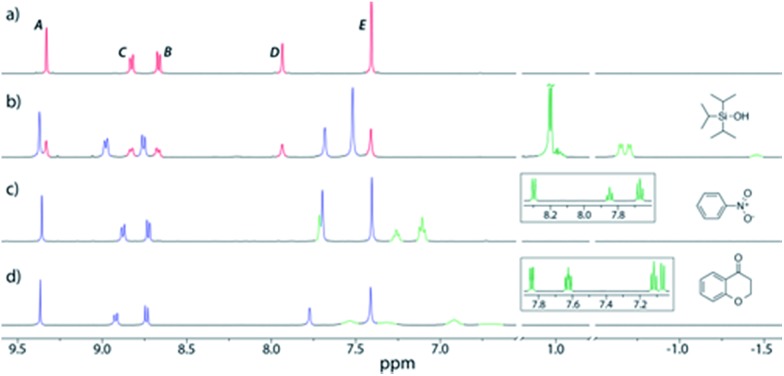
Partial ^1^H NMR spectra (500 MHz, D_2_O, 298 K) of (a) **2·**12NO_3_; (b) **2·**12NO_3_ with excess TIPSOH; (c) **2·**12NO_3_ with excess nitrobenzene; (d) **2·**12NO_3_ with excess chromanone. Color code: free capsule signals, red; bound capsule, blue; bound guest, pale green; free guest, dark green. The insets in spectra (c) and (d) show the partial ^1^H NMR spectra of free guests in D_2_O. The assignments correspond to the lettering in Scheme 1.

To predict the size of guest molecules that **2** ^12+^could bind, calculations were carried out using the atomic coordinates from the X-ray structure of **1** ^12+^,^20^ which revealed the volume of the empty cavity is 358 Å^3^ (see the ESI†). Application of the guidelines for suitable guests laid down by Rebek^21^ would indicate that molecules with volumes of 164–229 Å^3^ should likely be ideal. However, an initial exploration of hydrocarbons close to this size range (2-methylnaphthalene, 168 Å^3^; biphenyl, 183 Å^3^; fluorene, 189 Å^3^; phenanthrene, 201 Å^3^; anthracene, 201 Å^3^; pyrene, 220 Å^3^, *n*-dodecane, 235 Å^3^) showed no evidence for encapsulation. Instead, when excess triisopropylsilyl alcohol (TIPSOH) was added to a sample of **2·**12NO_3_, ^1^H NMR spectroscopy revealed the appearance of a new set of capsule signals (Fig. 2b) alongside those of free **2** ^12+^ (Fig. 2a). In addition, a set of upfield-shifted signals relative to free TIPSOH with equimolar intensity relative to the new capsule resonances, strongly suggest that one silyl guest is encapsulated within **2** ^12+^, and that exchange in and out of the cavity is slow on the NMR timescale. Further evidence for this encapsulation is provided by ^1^H NMR DOSY, which shows that the encapsulated TIPSOH species diffuses at the same rate as both the free and bound cage. Based on the molar ratios at equilibrium, the *K*_a_ of TIPSOH for **2** ^12+^ has been calculated to be *ca.* 1400 M^–1^, while EXSY gives the activation barrier for exchange of this guest as 17.3 kcal mol^–1^ (see the ESI†). Interestingly, the volume of TIPSOH (220 Å^3^) is quite a lot larger than 55% of the empty cavity, however, this could quite easily be a result of the relatively large portals into which the guest can protrude.

The effect of ionic strength on hydrophobically-driven guest encapsulation within synthetic host systems has been rarely studied,^22^ with respect to metallosupramolecular systems, this may be a result of instability towards high concentrations of salt. However, **2·**12NO_3_ is stable even in solutions of 5 M NaNO_3_ in D_2_O. Furthermore, an increasing affinity of the guest in 0.1 M, 1 M and 5 M NaNO_3_ D_2_O solutions is clearly observed through the decrease in % unbound species, such that at the highest salt concentration, free **2** ^12+^ is beyond the spectroscopic detection limit (see the ESI†). Through dilution experiments, it has been calculated that the affinity of the TIPSOH guest increases nearly four-fold in 5 M NaNO_3_ solution to 4700 M^–1^.

**Fig. 3:**
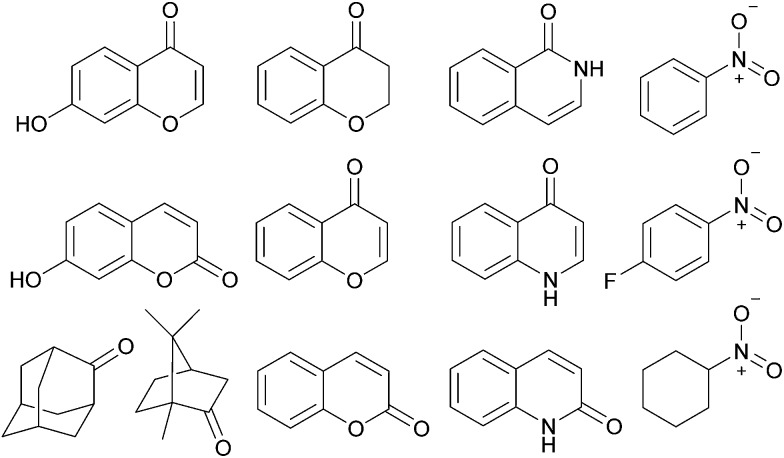
Guest molecules for **2** ^12+^ that exhibit fast exchange on the NMR timescale.

**Fig. 4:**
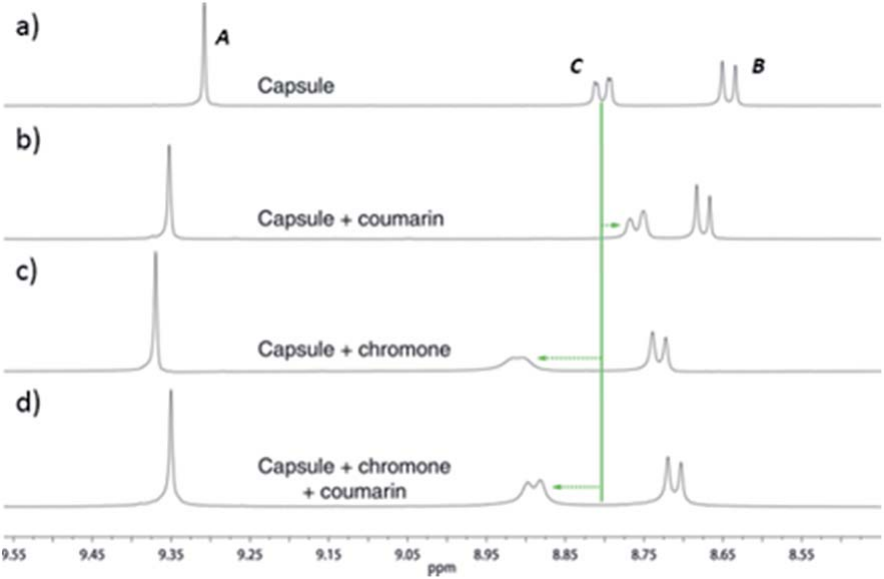
^1^H NMR spectra (500 MHz, D_2_O, 298 K) showing the preferential binding of chromone in the presence of coumarin. (a) **2·**12NO_3_ only; (b) **2·**12NO_3_ in the presence of coumarin; (c) **2·**12NO_3_ in the presence of chromone; (d) **2·**12NO_3_ in the presence of 1 : 1 coumarin and chromone.

In addition to TIPSOH, we have also found that a range of other organic molecules act as guests for **2** ^12+^ (Fig. 3). In contrast, these exhibit exchange fast on the NMR timescale, such that a single set of resonances are observed for both guest and host, for example, Fig. 2c and d, shows the ^1^H NMR spectra of **2·**12NO_3_ in the presence of excess nitrobenzene and chromanone. In these examples, the guest's signals are significantly upfield shifted with respect to the free species in the same solvent, consistent with being encapsulated and experiencing (time-averaged) shielding effects from the capsules' aromatic struts. Furthermore, for the majority of these guests, the direction in which the capsules' signals H_*A*–*E*_ shift are consistent, also similar to what is observed for TIPSOH encapsulation, thus indicating that guests bind in a conserved fashion within **2** ^12+^ (or otherwise cause a similar binding-induced re-organization). Interestingly, the molecules that act as guests could collectively be described as weakly amphiphilic. These general observations points to a mode of binding in which a specific guest functional group–cage interaction(s) is(are) supplemented by the hydrophobic effect.^17*d*^ A preliminary investigation into the relative affinities of some of the guests shown in Fig. 3 reveal that the regioisomeric compounds coumarin and chromone possess binding constants with a ten-fold difference, 120 M^–1^ and 1200 M^–1^, respectively (see the ESI†). This data is also supported by a competition binding experiment involving these two guests. Whereas coumarin-only binding causes an upfield shift in the H_*C*_ environment (Fig. 4b) with respect to free **2** ^12+^ (Fig. 4a), chromone encapsulation causes the same signal to become deshielded (Fig. 4c). In the presence of a 1 : 1 mixture of both analytes (Fig. 4d), this same signal is similarly deshielded, indicating the capsule is able to preferentially bind chromone in the presence of coumarin, showing that the capsule can differentiate molecules based on shape or the relative positioning of functional groups and not solely on the basis of more bulk descriptors.

## Conclusions

Coordination capsules almost always provide an opportunity to explore chemical equilibria, both at the level of the architecture self-assembly process and also due to their reversible interactions with guest molecules. Here we have reported a rare example of a coordination capsule which is not in equilibrium with its disassembled state. Similarly rare are coordination capsules which exhibit non-equilibrium guest binding properties.^23^ The development of metal-based (and fully organic) assemblies that are both constitutionally non-dynamic and also possess non-reversible guest binding properties,^24^ coupled with stimuli-responsive release mechanisms, could lead to improved function for a range of applications. As is the case in the field of synthetic molecular machines,^25^ we envisage that systems able to operate far away from equilibrium will be able to perform tasks not currently possible for their thermodynamic equivalents.
