# Supplementary material for: Non-equilibrium cobalt(iii) “click” capsules
Source: Chem Sci. 2014 Oct 7;6(1):756–60. doi: 10.1039/c4sc03036b (PMC5494537; doi:10.1039/c4sc03036b)
Supplement: Supplementary file 1 [file SC-006-C4SC03036B-s001.pdf]

Electronic Supplementary Material (ESI) for Chemical Science.
This journal is © The Royal Society of Chemistry 2014

Supporting Information

Non-Equilibrium Cobalt(III) "Click" Capsules

Paul R. Symmers,[†] Michael J. Burke,[†] David P. August,[†] Patrick I. T. Thomson,[†] Gary S. Nichol,[†] Mark Warren,[‡] Colin Campbell[†] and Paul J. Lusby.[†,*]

[†]EaSTCHEM School of Chemistry, University of Edinburgh, The King's Buildings, West Mains Road, Edinburgh EH9 3JJ, UK.

[‡]Diamond Light Source Ltd., Diamond House, Harwell Science and Innovation Campus, Didcot, Oxfordshire, OX11 0DE.

Email: Paul.Lusby@ed.ac.uk

1. General Information S2
2. Syntheses S4
3. Electrochemistry S13
4. Constitutional Dynamic Experiments S15
5. Cage Cavity Modelling S19
6. Host-Guest Chemistry S20
7. NMR Spectra of all compounds S40
8. Mass spectrometry of complexes S55
9. X-ray crystallography S58
10. References S107

1. General Information

All reagents were purchased from Sigma-Aldrich, VWR or Alfa-Aesar and used without further purification. Where the use of anhydrous solvent is stated, drying was carried out using a solvent purification system manufactured by Innovative Technology, Newburyport, MA, USA. Column chromatography was performed using Kieselgel 60 (particle size 35-70) microns as the stationary phase and TLC ran on precoated silica 60 gel plates (0.20 mm thick, $60F_{254}$. Merck, Germany) and observed under UV light (unless stated otherwise). All reactions were carried out under N_2 atmosphere, unless stated otherwise. Degassing of solvents was carried out using vacuum-N_2 backfill cycles on standard Schlenk apparatus. An ultrasonic bath was applied during the vacuum half of the cycle to enhance gas release from the liquid.

All ^{1}H and ^{13}C NMR spectra were recorded, as stated, on either Bruker AV400 or AV500 at a constant temperature of 298K (unless stated). All DOSY experiments were recorded on Bruker AV500 (Topspin 2.1) using bipolar gradient pulses for diffusion with two spoil gradients (ledbpg2s.compensated) pulse sequence. The sequence was carried out under automated conditions where the duration of the magnetic pulse gradient was 1.5 ms and the diffusion time was 100 ms. Typically in each PFG NMR experiment, a series of 16 spectra on 32K data points were collected and the eddy current delay was set to 5 min in all experiments. The pulse gradients were incremented from 2 to 95% of the maximum gradient strength in a linear ramp. The temperature was set and controlled at 300K with an air flow of 400 L h^{-1} in order to avoid any temperature fluctuations due to sample heating during the magnetic field pulse gradients. The Stokes-Einstein equation was used to convert diffusion coefficient to hydrodynamic radius. The data was processed using Bruker Topspin 2.1 and MestreLab Research MestReNova 6.0.3. Chemical shifts are reported in parts per million from low to high field and are referenced against values for the residual solvent peaks. Coupling constants (*J*) are reported as observed in Hz. Standard abbreviations indicating multiplicity are used as follows: m = multiplet, t = triplet, d = doublet, s = singlet, br(s/d) = broad (singlet/doublet *etc.*).

Mass spectrometry of organic compounds was carried out on a high resolution Bruker ToF instrument. Mass spectrometry (ESI-MS) of complexes was carried out using either a Micromass Q-Tof2 or a Waters SYNAPT G2 instrument, ion-mobility mass spectrometry

(ESI-IM-MS) was carried out with a Waters SYNAPT G2 instrument with N_2 as a drift cell gas.

2. Syntheses

General Reaction Scheme for Synthesis of Ligands

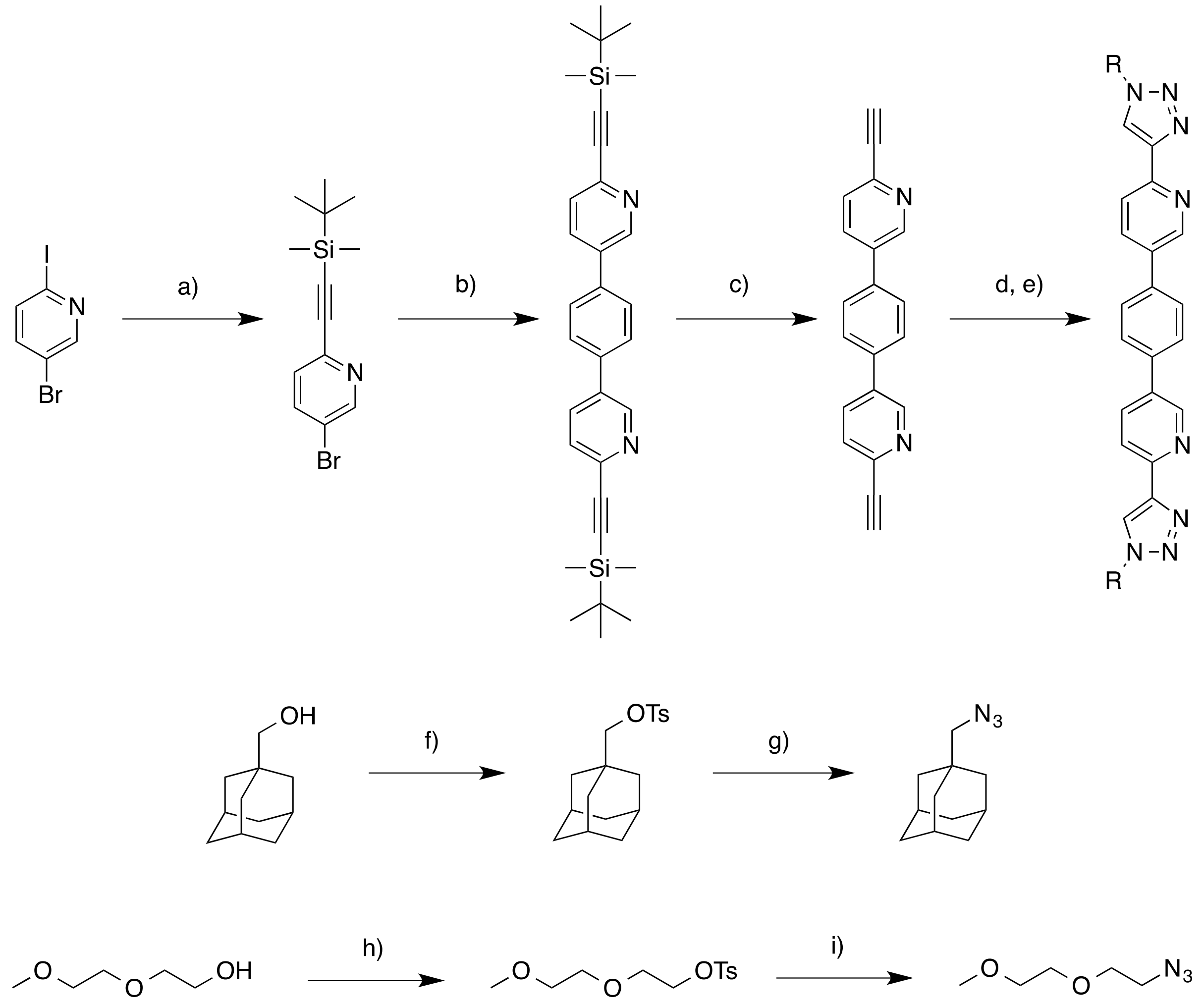

Figure S1: Reaction schemes for synthesis of **L**[1] and **L**[2]. Reaction conditions and yields: a) tert-butyldimethylsilylacetylene, copper(I) iodide, triphenylphosphine, palladium(II) acetate, THF, NEt_3,18 h, RT, 74%; b) Benzene-1,4-diboronic acid, palladium(II) tetrakistriphenylphosphine, sodium carbonate, THF / H_2O / EtOH, 80 °C, 16 h, 69%; c) Tetrabutylammonium fluoride, THF, H_2O, RT, 1.5 h, 95%; d) adamantyl azide, DIPEA, $[Cu(CH_3CN)_4]PF_6$, TBTA, DCE, 90 °C, 42 h, 80%; e) 2PEG azide, Cu/C, triethylamine, DCE, 65 °C, 48h, 86%; f) Tosyl chloride, pyridine, RT, 14 h, 86%; g) sodium azide, DMSO, 80 °C, 48 h, 78%; h) Tosyl chloride, sodium hydroxide, THF, 0-12 °C then RT, 120 h, 87%; i) sodium azide, DMF, 50 °C, 39 h, 92%.

2-tert-butyldimethylsilylethynyl-5-bromopyridine

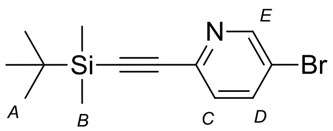

A degassed solution of 5-bromo-2-iodopyridine (5.06 g, 17.8 mmol) in THF (100 mL) was charged to a 3-necked flask containing PPh_3 (468 mg, 1.78 mmol)), $Pd(OAc)_2$ (205 mg, 0.91 mmol) and CuI (256 mg, 1.34 mmol). The mixture was cooled to 0 °C and (*tert*-butyldimethylsilyl)acetylene (2.48 g, 17.6 mmol), then triethylamine (27 mL) were added. The mixture was allowed to warm to RT and stirred for 18 h under N_2. The reaction mixture was concentrated *in vacuo*, then CH_2Cl_2 and H_2O were added. The organic layer was separated, dried ($MgSO_4$), filtered and the solvent removed under reduced pressure. The crude product was purified using a silica flash column (hexane-CH_2Cl_2, 2:1) to give the title compound as a colourless powder. Yield = 3.91 g (74%). m.p. 89-92 °C. ^{1}H NMR (400 MHz, $CDCl_3$): δ8.62 (dd, J = 2.3, 0.6 Hz, 1H, H_E), 7.77 (dd, J = 8.3, 2.4 Hz, 1H, H_D), 7.34 (dd, J = 8.3, 0.7 Hz, 1H, H_C), 1.00 (s, 9H, H_A), 0.20 (s,6H, H_B). ^{13}C NMR (126 MHz, $CDCl_3$): δ151.2, 141.6, 138.9, 128.6, 120.4, 103.5, 95.1, 26.3, 16.8, -4.7. HR-ESI MS (*m/z*): 296.04810 (predicted $[M+H]^+$ = 296.04647), 318.02950 (predicted $[M+Na]^+$ = 318.02841).

1,4-bis(6-((tert-butyldimethylsilyl)ethynyl)pyridin-3-yl)benzene

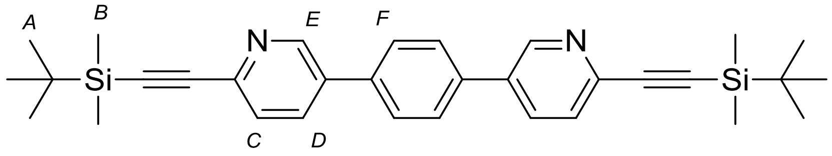

A degassed solution of 2-*tert*-butyldimethylsilylethynyl-5-bromopyridine (307 mg, 1.04 mmol) and $Pd(PPh_3)_4$ (60.7 mg, 52.5 µmol) in THF (5 mL) was charged to a 2-necked flask equipped with reflux condenser under N_2. A degassed solution of Na_2CO_3 (280 mg, 2.64 mmol) in H_2O (2.5 mL) was added *via* cannula, then a degassed solution of benzene-1,4-diboronic acid (86.0 mg, 0.52 mmol) in EtOH (4 mL), after which a precipitate started to form. The mixture was heated at 80 °C for 16 h under N_2. The precipitate was isolated by filtration. The crude product was purified using a silica flash column (CH_2Cl_2) to give the title compound as colourless crystalline solid. Yield = 183 mg (69%). m.p. 276-278 °C. ^{1}H NMR (600 MHz, $CDCl_3$): δ8.86 (dd, J = 2.3, 0.7 Hz, 2H, H_E), 7.88 (dd, J = 8.1, 2.4 Hz, 2H,

H_D), 7.70 (s, 4H, H_F), 7.55 (dd, J = 8.1, 0.7 Hz, 2H, H_C), 1.03 (s, 18H, H_A), 0.23 (s, 12H, H_B). ^{13}C NMR (126 MHz, $CDCl_3$): δ148.5, 142.3, 137.4, 135.0, 134.3, 128.0, 127.6, 104.5, 94.5, 26.3, 16.9, -4.6. HR-ESI MS (*m/z*): 509.27730 (predicted $[M+H]^+$ = 509.28028), 531.25960 (predicted $[M+Na]^+$ = 531.26223).

1,4-bis(6-ethynylpyridin-3-yl)benzene

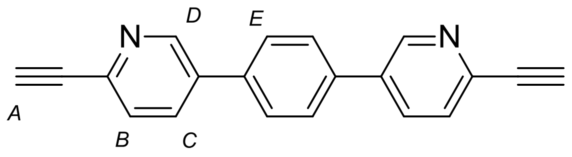

To a suspension of 1,4-*bis*(6-((tert-butyldimethylsilyl)ethynyl)pyridin-3-yl)benzene (264 mg, 0.52 mmol) in THF (15 mL) and H_2O (0.2 mL), tetrabutylammonium fluoride solution (1.0M in THF, 1.15 mL, 1.15 mmol) was charged. The mixture was stirred at RT under N_2 for 1.5 h, when TLC showed full consumption of starting material. Aqueous NH_4Cl saturated solution (3 mL) was added and the organic layer was removed *in vacuo*. CH_2Cl_2 (150 mL) and H_2O (50 mL) were added, and the organic layer was separated and washed with more H_2O (50 mL) and brine (30 mL). The organic layer was separated, dried ($MgSO_4$), filtered and the solvent removed *in vacuo*. The crude product was purified using a silica flash column (CH_2Cl_2:CH_3OH, 200:1) to give the title compound as a colourless powder. Yield = 139 mg (95%). ^{1}H NMR (400 MHz, CD_2Cl_2): δ8.88 (dd, J = 2.4, 0.7 Hz, 2H, H_D), 7.94 (dd, J = 8.1, 2.4 Hz, 2H, H_C), 7.76 (s, 4H, H_E), 7.59 (dd, J = 8.1, 0.7 Hz, 2H, H_B), 3.26 (s, 2H, H_A). ^{13}C NMR (126 MHz, CD_2Cl_2): δ148.8, 141.6, 137.5, 135.7, 134.6, 128.2, 127.9, 83.1, 77.8. HR-ESI MS (*m/z*): 281.10680 (predicted $[M+H]^+$ = 281.10733).

1-adamantanemethyltosylate

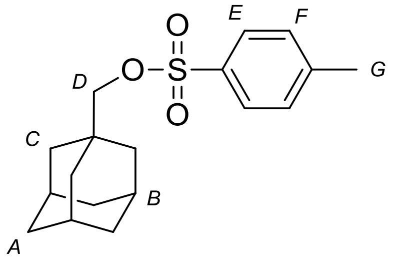

To a solution of 1-adamantanemethanol (2.58 g, 15.5 mmol) in pyridine (12 mL) cooled to 0 °C, *p*-toluenesulfonylchloride (4.45 g, 23.3 mmol) was added to give a pale yellow solution. The mixture was stirred at RT for 14h before the solvent was removed *in vacuo*. EtOAc (200 mL) was added, and after filtration, the organic solvent was washed with saturated $NaHCO_3$ solution (2 x 100 mL) and brine (100mL). The organic layer was separated, dried ($MgSO_4$), filtered and the solvent removed *in vacuo*. The crude product was dissolved in toluene (150 mL) and *N,N*-dimethylethylenediamine (1.03 g, 11.6 mmol) was added. The solution was stirred for 0.5 h, before H_2O (100 mL) was added and the organic layer separated. The crude product was purified using a silica flash column (CH_2Cl_2) to give the title compound as a colourless crystalline solid. Yield = 4.27 g (86%). ^{1}H NMR (400 MHz, $CDCl_3$): δ 7.77 (d, J = 8.2 Hz, 2H, H_E), 7.34 (d, J = 8.1 Hz, 2H, H_F), 3.55 (s, 2H, H_D), 2.45 (s, 3H, H_G), 1.95 (br s, 3H, H_B), 1.69 (br d, J = 12.2 Hz, 3H, H_C), 1.60 (br d, J = 12.1 Hz, 3H, H_C), 1.47 (s, 6H, H_A). ^{13}C NMR (126 MHz, $CDCl_3$): δ 144.7, 133.3, 129.9, 128.1, 79.9, 38.8, 36.8, 33.5, 28.0, 21.8. HR-ESI MS (*m/z*):343.13520 (predicted $[M+Na]^+$ = 343.13384).

1-adamantanemethylazide

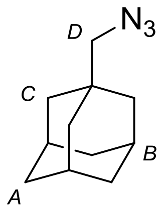

To a solution of NaN_3 (2.27g, 34.9 mmol) in DMSO (70 mL) was added 1-admantanemethyltosylate (3.73g, 11.6 mmol). The mixture was heated at 80 °C for 48 h under N_2. H_2O (140 mL) was added to induce the precipitation of a white solid. This was then extracted with EtOAc (200 mL) and the organic layer washed with H_2O (140 mL) and

brine (70 mL), dried ($MgSO_4$), filtered and the solvent removed *in vacuo*. The crude product was purified using a silica flash column (hexane) to give the title compound as a clear, colourless oil. Yield = 1.73 g (78%). The staining mixture described by Schröder, Gartner, Grab and Bräse was used to visualise product on TLC and confirm the presence of the azide functional group.[1] ^{1}H NMR (400 MHz, $CDCl_3$): δ 2.95 (s, 2H, H_D), 1.99 (br s, 3H, H_B), 1.72 (br d, J = 12.2 Hz, 3H, H_C), 1.64 (br d,J = 12.4 Hz,3H, H_C), 1.52 (br s, 6H, H_A). ^{13}C NMR (126 MHz, $CDCl_3$): δ 64.5, 40.2, 37.0, 34.9, 28.3. HR-ESI MS (*m/z*):191.14183 (predicted $[M+H]^+$ = 191.14170).

2-(2-methoxyethoxy)ethyl 4-methylbenzenesulfonate

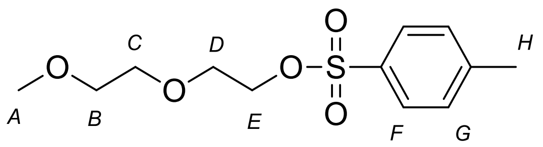

To a solution of 2-(2-methoxyethoxy)ethanol (20.0 g, 167 mmol) in THF (60 mL) at 0 °C was added a solution of NaOH (14.0 g, 351 mmol) in H_2O (60 mL) over 1.5 h, ensuring the internal temperature kept below 0 °C. A solution of tosyl chloride (38.4 g, 201 mmol) in THF (100 mL) was then added over 1.5 h. The biphasic mixture was allowed to warm to RT, and stirred for 120 h under N_2. Et_2O (200 mL) and NaOH (2.5 M, 50 mL) were added, and the organic layer was separated. The aqueous phase was washed with Et_2O (50 mL), and the combined organic fractions were washed with H_2O (2 x 100 mL) and brine (50 mL). The organic layer was separated, dried ($MgSO_4$), filtered and concentrated *in vacuo* to give the title compound as a clear, colourless oil. Yield = 39.9 g (87%). ^{1}H NMR (500 MHz, $CDCl_3$): δ 7.80 (d, J = 8.3 Hz, 2H, H_F), 7.33 (d, J = 8.6 Hz, 2H, H_G), 4.16 (dd, J = 5.4, 4.4 Hz, 2H, H_E), 3.71 – 3.65 (m, 2H, H_D), 3.60 – 3.54 (m, 2H, H_C), 3.50 – 3.45 (m, 2H, H_B), 3.34 (s, 3H, H_A), 2.44 (s, 3H, H_H). ^{13}C NMR (126 MHz, $CDCl_3$): δ 144.9, 133.1, 129.9, 128.1, 71.9, 70.8, 69.3, 68.8, 59.2, 21.8. HR-ESI MS (*m/z*): 275.09580 (predicted $[M+H]^+$ = 275.09477).

1-azido-2-(2-methoxyethoxy)ethane

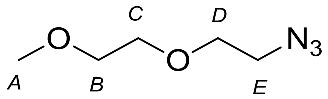

NaN_3 (36.6 g, 563 mmol) was added to a solution of 2-(2-methoxyethoxy)ethyl tosylate (30.9 g, 112 mmol) in DMF (150 mL). The mixture was stirred at 50 °C for 39 h under N_2. Et_2O (100 mL) and H_2O (100 mL) were added, the organic layer was separated, washed with H_2O, dried ($MgSO_4$) and filtered and the solvent removed *in vacuo* to give the title compound as a clear, colourless oil. Yield = 15.1 g (92%). The presence of the azide functional group was confirmed using the method described by Schröder *et al.*[1] ^{1}H NMR (500 MHz, $CDCl_3$): δ 3.65 – 3.58 (m, 4H, $H_{D,E}$), 3.53 – 3.49 (m, 2H, H_C), 3.38 – 3.31 (m, 5H, $H_{A,B}$). ^{13}C NMR (126 MHz, $CDCl_3$): δ 71.9, 70.5, 70.0, 59.0, 50.6. HR-ESI MS (*m/z*): 146.09320 (predicted $[M+H]^+$ = 146.09240).

1,4-bis(1-adamantanemethyl)-1H-1,2,3-triazol-4-yl)pyridin-3-yl)benzene (L^1)

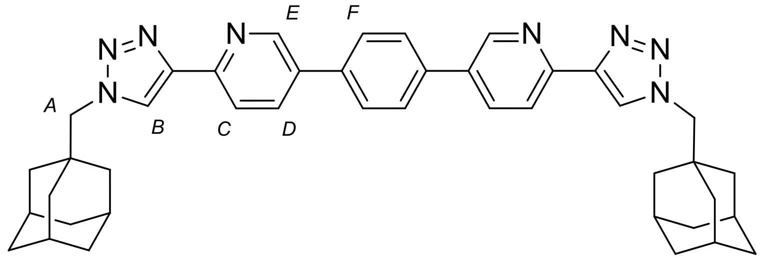

To a solution of 1,4-bis(6-ethynylpyridin-3-yl)benzene (82.8 mg, 0.295 mmol) in DCE (80 mL), adamantanemethylazide (466 mg, 2.44 mmol) and diisopropylethylamine (352 mg, 2.7 mmol) were added, followed by $[Cu(CH_3CN)_4]PF_6$ (58.4 mg, 0.157 mmol) and tris[(1-benzyl-1*H*-1,2,3-triazol-4-yl)methyl]amine (99.6 mg, 0.188 mmol). The reaction was then heated at 90 °C for 42 h under N_2. The resultant precipitate was isolated by filtration, washed with DCE and CH_3CN and dried under vacuum to give the title compound as a colourless solid. Yield = 156 mg (80%). m.p. 359-362 °C. The product is highly insoluble in most solvents, therefore characterisation by NMR was achieved in a mixture of deuterated acetonitrile and deuterated trifluoroacetic acid (~10:1). The value of the residual acetonitrile peak is taken to be δ1.94 (for ^{1}H) or δ118.26 (for ^{13}C), and product peaks are referenced against that value. ^{1}H NMR (400 MHz, CD_3CN-TFA-*d*): δ 8.96 (br s, 2H, H_E), 8.92 (br d, *J*

= 8.8 Hz, 2H, H_C), 8.61 (br s, 2H, H_B), 8.43 (br d, J = 8.6 Hz, 2H, H_D), 8.03 (s, 4H, H_F), 4.24 (s, 4H, H_A), 2.00 (br s, 4H, $H_{adamantane}$), 1.78 – 1.54 (m, 26H, $H_{adamantane}$). ^{13}C NMR (126 MHz, CD_3CN): δ 169.7, 146.8, 143.1, 139.9, 138.8, 136.0, 129.6, 128.8, 125.5, 63.4, 55.9, 52.6, 45.5, 35.1. HR-ESI MS (*m/z*): 663.39206 (predicted $[M+H]^+$ = 663.39182).

1,4-bis(2-(1-(2-(2-methoxyethoxy)ethyl)-1H-1,2,3-triazol-4-yl)pyridin-3-yl)benzene (L²)

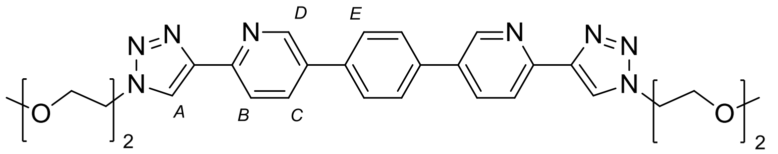

The Cu/C catalyst was prepared as described by Lipshutz and Taft.[2] A solution of 1,4-*bis*(6-ethynylpyridin-3-yl)benzene (174 mg, 0.621 mmol) in DCE (75 mL) was added to a flask containing Cu/C catalyst (272 mg) and PEG azide (732 mg, 4.54 mmol). To this triethylamine (0.44 mL, 3.16 mmol) was added and the mixture was stirred for 48 h at 65 °C under N_2. The mixture was hot filtered through celite (which was subsequently rinsed with hot DCE). The solvent was removed *in vacuo*, then CH_2Cl_2 (50 mL) and Na_4EDTA aqueous solution (100 mL, 0.1 M) were added. The mixture was stirred for 0.25 h before the organic layer was separated, washed with H_2O (50 mL), dried ($MgSO_4$), filtered and the solvent removed *in vacuo*. The crude product was purified using a silica flash column (CH_2Cl_2-CH_3OH-Et_3N, 98:1:1) to give the title compound as a colourless solid. Yield = 323 mg (86%). m.p. 223-225 °C. ^{1}H NMR (400 MHz, DMSO) δ 9.02 (dd, J = 2.4, 0.8 Hz, 2H, H_D), 8.64 (s, 2H, H_A), 8.29 (dd, J = 8.3, 2.4 Hz, 2H, H_C), 8.14 (dd, J = 8.3, 0.7 Hz, 2H, H_B), 7.95 (s, 4H, H_E), 4.63 (t, J = 5.2 Hz, 4H, H_{PEG}), 3.90 (t, J = 5.2 Hz, 4H, H_{PEG}), 3.60 – 3.54 (m, 4H, H_{PEG}), 3.46 – 3.40 (m, 4H, H_{PEG}), 3.22 (s, 6H, $H_{O\text{-}CH3}$). ^{13}C NMR (126 MHz, DMSO): δ 149.2, 147.6, 146.9, 136.4, 135.0, 133.7, 127.4, 123.7, 119.4, 71.1, 69.4, 68.6, 58.1, 49.6. HR-ESI MS (*m/z*): 571.27650 (predicted $[M+H]^+$ = 571.27758), 593.25840 (predicted $[M+Na]^+$ = 593.25952).

Co_4L_6 Tetrahedra Syntheses

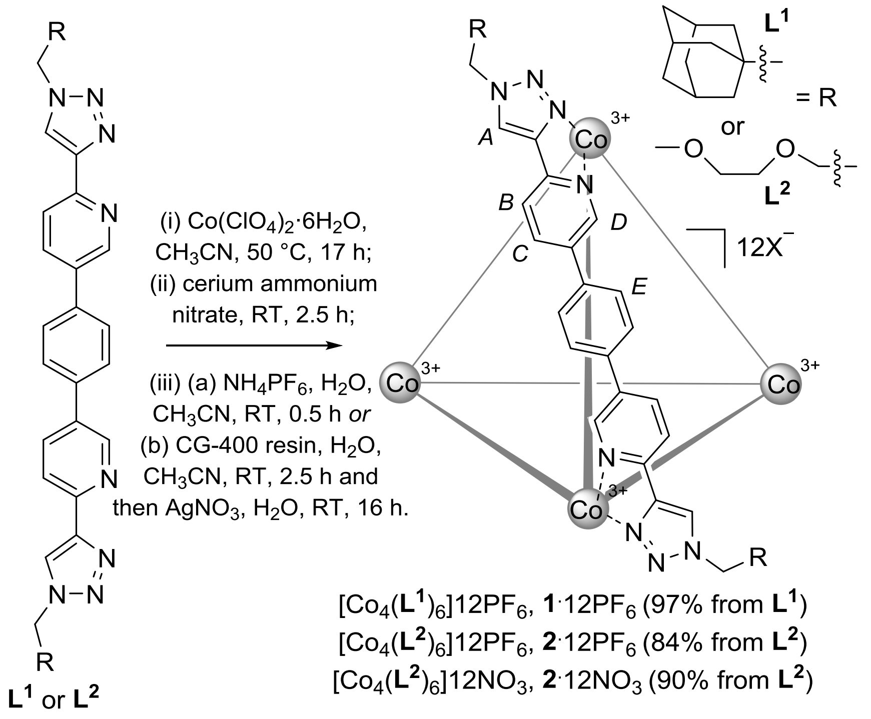

Figure S2: Reaction scheme for the synthesis of **1**·12PF6, **2**·12PF6 and **2**·12NO3.

$[Co_4(L^1)_6]12PF_6$, 1·12PF6

To a suspension of **L**[1] (28.9 mg, 43.6 μmol) in degassed CH_3CN (50 mL) was added $Co(ClO_4)_2.6H_2O$ (10.5 mg, 28.7 μmol). Following further degassing, the solution was heated at 50 °C for 17 h under N_2. The reaction was allowed to cool to RT before ammonium cerium(IV) nitrate (24.0 mg, 43.8 μmol) in CH_3CN (60 mL) was added dropwise over 1.5 h. The suspension was stirred for 1 h then filtered on to celite, washed with CH_3CN (20 mL) and released into solution with H_2O-CH_3CN (1:1). The addition of NH_4PF_6 (140 mg, 859 μmol) to this solution resulted in the formation of an orange precipitate, which was removed by filtration on to celite, which was then washed with H_2O and dissolved in CH_3CN before the solvent was removed *in vacuo* to give the title compound as an orange solid. Yield = 42.0 mg (97%). ^{1}H NMR (500 MHz, CD_3CN): δ8.89 (s, 12H, H_A), 8.79 (dd, *J* = 8.4, 1.5 Hz, 12H, H_C), 8.55 (d, *J* = 8.4 Hz, 12H, H_B), 7.65 (d, *J* = 1.5 Hz, 12H, H_D), 7.47 (s, 24H, H_E), 4.20 (s, 24H, H_F), 2.06 – 2.00 (br m, 36H, H_{adam}), 1.80 (br d, *J* = 12.2 Hz, 36H, H_{adam}), 1.63 (br d, *J* = 11.7 Hz, 36H, H_{adam}), 1.50 (br s, 72H, H_{adam}). ^{13}C NMR (126 MHz, CD_3CN): δ150.1, 148.4, 148.0, 142.9, 140.4, 135.6, 130.1, 128.7, 126.2, 66.0, 41.4, 37.0, 35.2, 29.0. ^{1}H DOSY NMR

(500 MHz, CD_3CN): Log *D* = −9.33 $m^2 s^{-1}$; calculated hydrodynamic radius = 12.6 Å. ESI-MS (*m/z*): 1343 (+4), 1045 (+5), 847 (+6), 705 (+7) (see Section 7 for full spectrum, expansions of each charge state and isotopic distributions).

$[Co_4(L^2)_6]12PF_6$, $2\cdot12PF_6$

$\mathbf{L^2}$ (40.0 mg, 70.1 μmol) was used to give the title compound as an orange solid following the same procedure as described for $\mathbf{L^1}$. Yield = 53.0 mg (84%). ^{1}H NMR (500 MHz, CD_3CN): δ9.04 (s, 12H, H_A), 8.76 (dd, *J* = 8.4, 1.8 Hz, 12H, H_C), 8.54 (d, *J* = 8.4 Hz, 12H, H_B), 7.69 (d, *J* = 1.6 Hz, 12H, H_D), 7.46 (s, 24H, H_E), 4.71 – 4.55 (m, 24H, H_F), 3.91 – 3.79 (m, 24H, H_{PEG}), 3.60 – 3.48 (m, 24H, H_{PEG}), 3.48 – 3.37 (m, 24H, H_{PEG}), 3.25 (s, 36H, H_{PEG}). ^{13}C NMR (126 MHz, CD_3CN): δ150.1, 148.4, 148.0, 143.0, 140.4, 135.5, 129.7, 128.7, 126.1, 72.3, 70.9, 68.6, 58.9, 55.0. ^{1}H DOSY NMR (500 MHz, CD_3CN): Log *D* = -9.34 $m^2 s^{-1}$; calculated hydrodynamic radius = 12.8 Å. ESI-MS (*m/z*): 1205 (+4), 935 (+5), 755 (+6), 626 (+7), 530 (+8) (see Section 7 for full spectrum, expansions of each charge state and isotopic distributions).

$[Co_4(L^2)_6]12NO_3$, $2\cdot12NO_3$

$\mathbf{L^2}$ (148 mg, 0.260 mmol) was used to generate the oxidised mixed counteranion species, following the same procedure as described for $\mathbf{L^1}$. Then this was dissolved in H_2O-CH_3CN (25 mL, 2:1), and CG-400 resin (1.40 g) was added. The mixture was gently stirred for 2.5 h at RT then filtered through celite to remove the resin. The solvent was removed *in vacuo,* redissolved in H_2O (20 mL) and $AgNO_3$ (84.8 mg, 0.499 mmol) was added. The reaction was stirred for 2 h in the dark, then for a further 14 h with exposure to light. Celite filtration was carried out to remove the AgCl precipitate that formed during the reaction, and the solution was freeze dried to give the title compound as an orange solid. Yield = 172 mg (90%). ^{1}H NMR (400 MHz, D_2O, 343 K) δ9.30 (s, 12H, H_A), 8.81 (dd, *J* = 8.4, 1.6 Hz, 12H, H_C), 8.68 (d, *J* = 8.3 Hz, 12H, H_B), 7.83 (d, *J* = 1.0 Hz, 12H, H_D), 7.36 (s, 24H, H_E), 4.80 (br t, *J* = 4.7 Hz, 24H, H_{PEG}), 3.99 – 3.85 (m, 24H, H_F), 3.52 (m, 12H, H_{PEG}), 3.47 – 3.28 m, 36H, H_{PEG}), 3.14 (s, 36H, H_{PEG}). ^{13}C NMR (126 MHz, D_2O, 298 K): δ148.9, 147.3, 147.2, 142.2, 140.4, 134.6, 128.7, 127.7, 125.3, 70.8, 69.5, 67.9, 57.9, 53.8. ^{1}H DOSY NMR (500 MHz, D_2O): Log *D* = -9.76 $m^2 s^{-1}$; calculated hydrodynamic radius = 14.1 Å. ESI-MS (*m/z*): 1039 (+4), 819 (+5), 672 (+6), 567 (+7).

3. Electrochemistry

All electrochemical experiments were carried out in CH_3CN (degassed, anhydrous) with tetrabutylammonium hexafluorophosphate (100 mM) and **2.**$12PF_6$ (1 mM) in a 7 mL vial with a Saturated Calomel reference Electrode (SCE), platinum gauze (0.1mm) counter electrode, and platinum wire (0.5mm) working electrode. All platinum was cleaned by washing and heating with a blowtorch prior to use, and the experiment was maintained under a blanket of argon during use. Data was collected with an AutoLab potentiostat controlled by GPES software. Background scans indicated a wide solvent window, not interfering with the desired ranges (a minimum of -2 Volts SCE, -1756 mV NHE). Collected data at differing scan rates is shown in Figures S3 and 4.

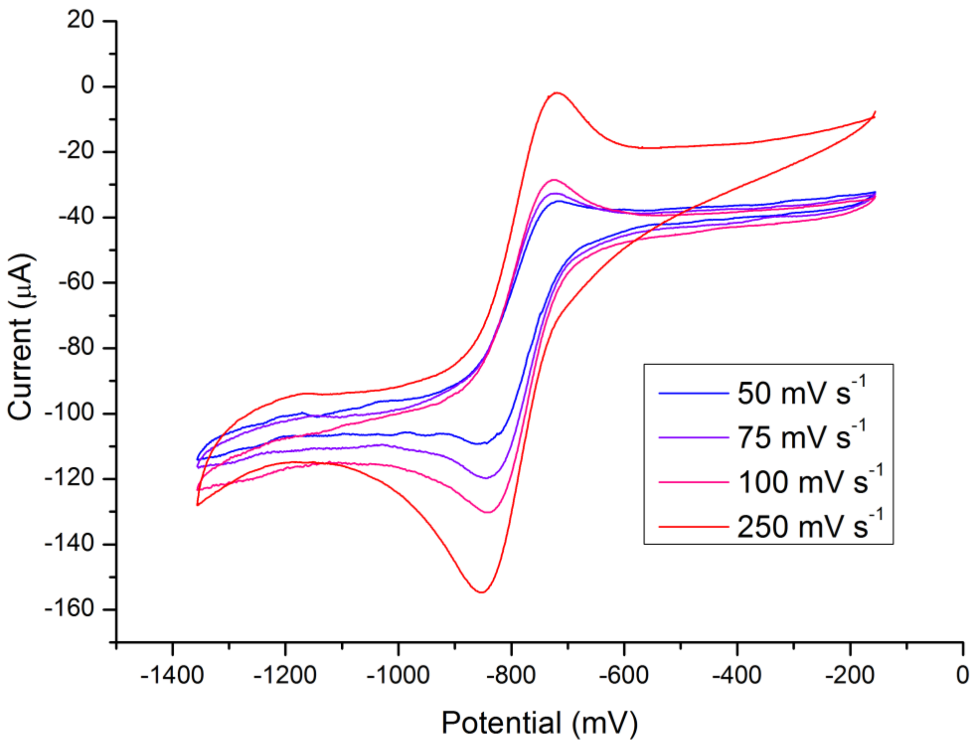

Figure S3: Cyclic voltammetry measurements of **2**.$12PF_6$ between -0.15 V and -1.4 V at various scan rates, showing reversible reduction and re-oxidation.

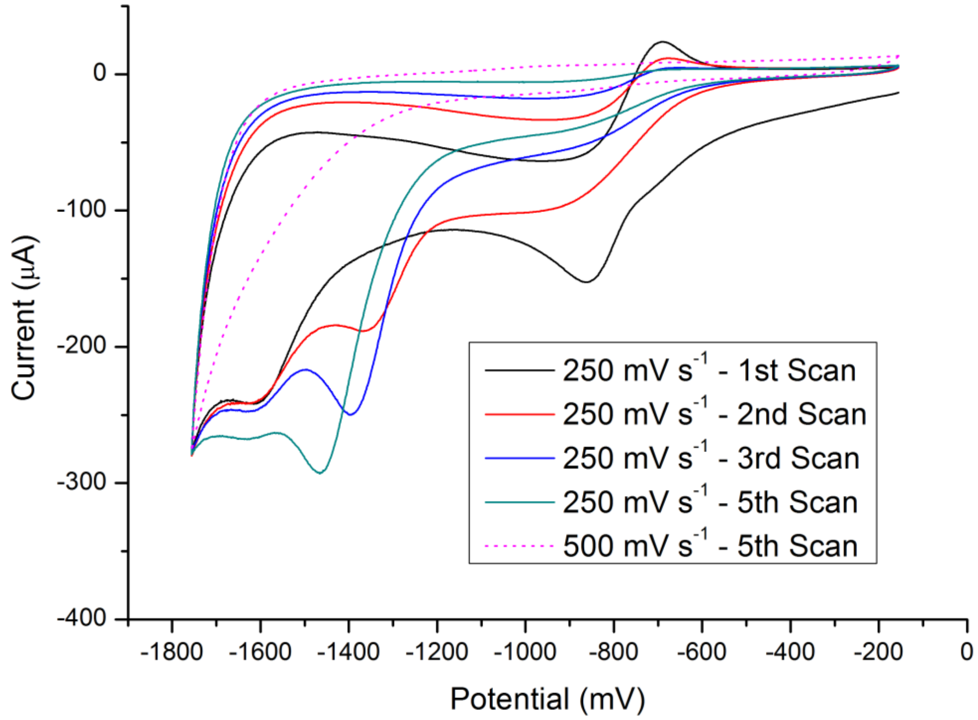

Figure S4: Cyclic voltammetry measurements of **2**.12PF_6 between -0.15 V and -1.8 V over 5 scans, showing the irreversible reduction of the complex. The 5th scan at 500 mV s^{-1} is also included to demonstrate the reduction becoming limited by diffusion of oxidized material to the electrode.

4. Constitutional Dynamic Experiments

Ligand Exchange between 1·12PF$_6$ and 2·12PF$_6$

2.5 mM solutions of **1**·12PF$_6$ and **2**·12PF$_6$ in CD_3CN (0.3 mL each) were combined in an NMR tube. The tube was capped and sealed with Teflon tape to prevent any solvent loss upon heating. The reaction was left at RT for 7 days, then heated at 50 °C for 7 days, then at 60 °C for 7 days and finally at 70 ºC for a further 24 h. NMR spectra were recorded at regular intervals. In addition, small samples were taken and diluted in CH_3CN for ESI-MS and ESI-IM-MS analysis.

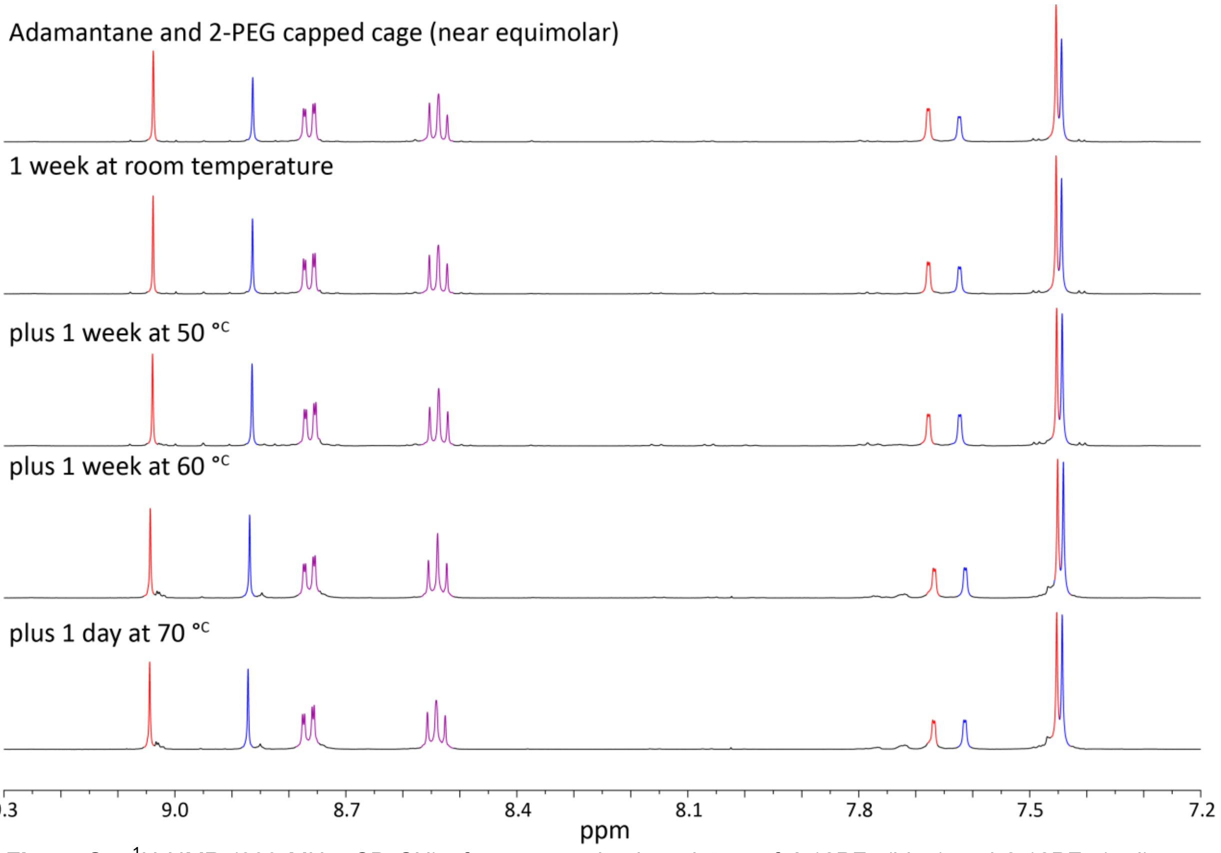

Figure S5: ^{1}H NMR (600 MHz, CD_3CN) of a near equimolar mixture of **1**·12PF$_6$ (blue) and **2**·12PF$_6$ (red) as a function of temperature and time. Peaks shown in purple represent indistinguishable overlap of signals from both complexes. No change over time was observed in regions outside those shown.

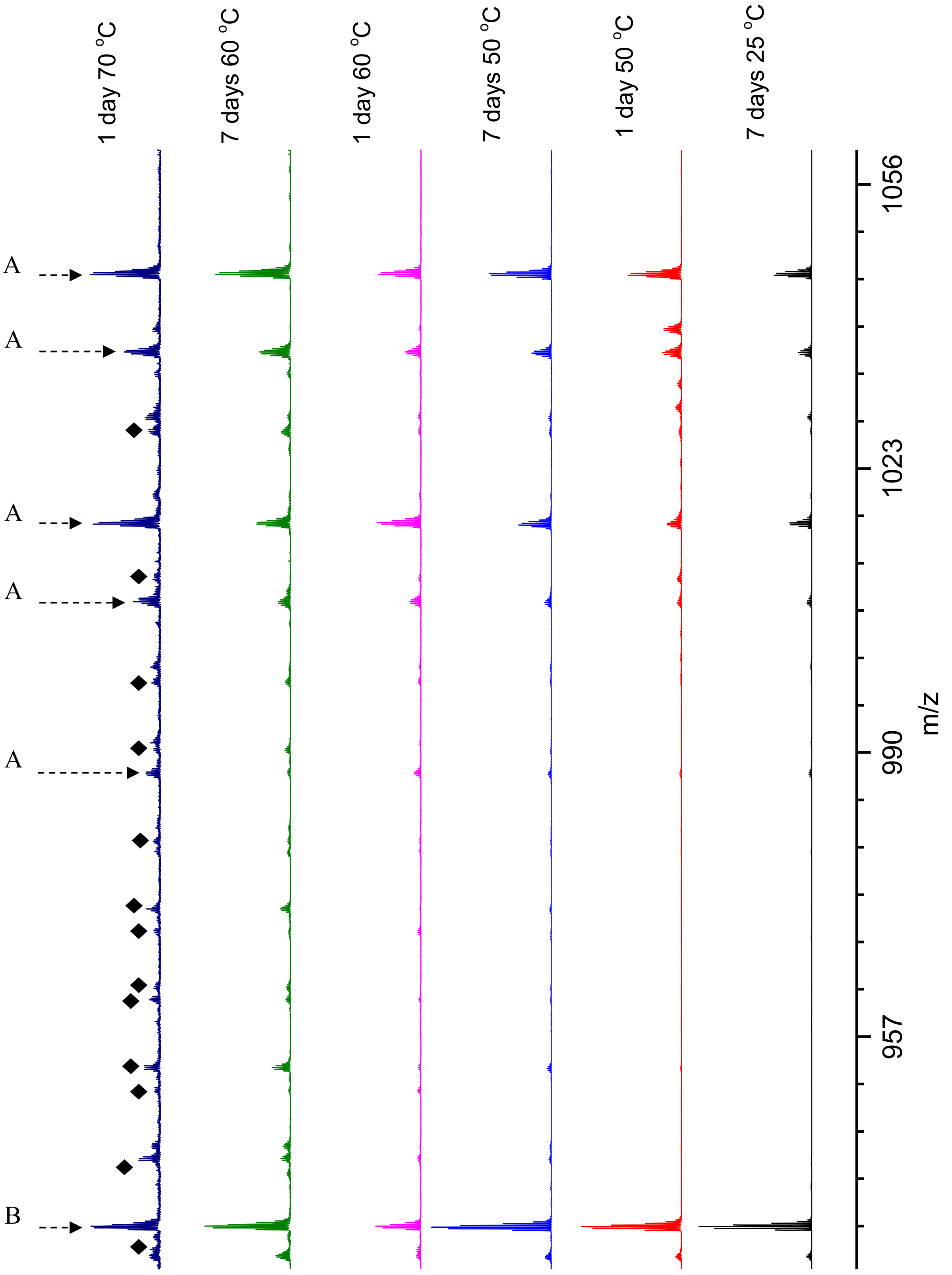

Figure S6: ESI-MS of a near equimolar mixture of **1**·12PF$_6$ (**A**) and **2**·12PF$_6$ (**B**) over time. 860-1060 m/z range shown to illustrate ligand scrambling between the two species. Mixed ligand species are marked as ◆.

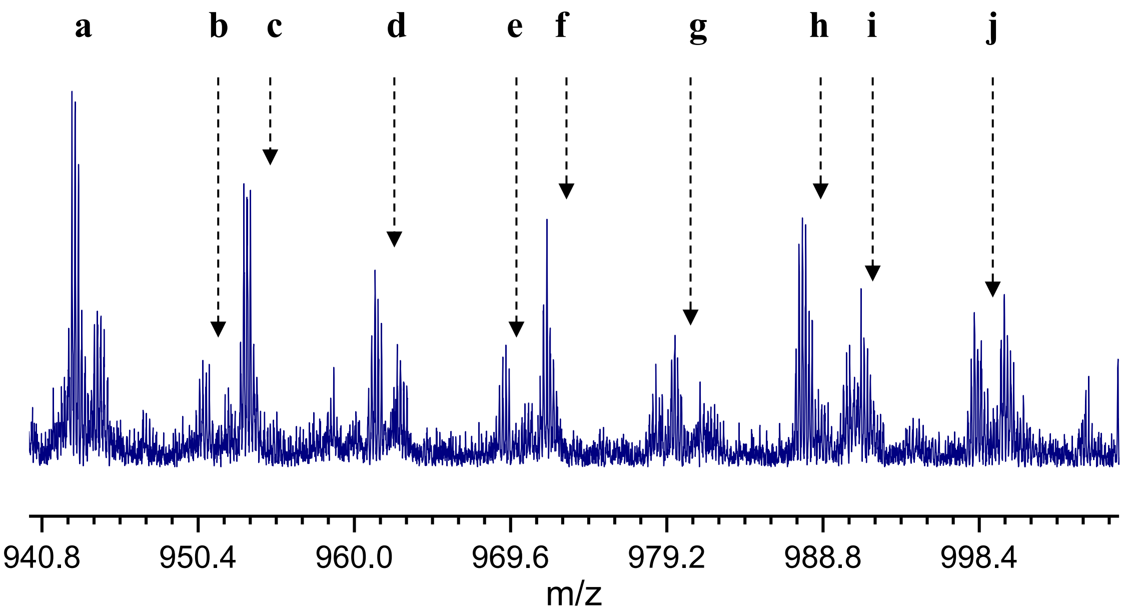

Species	Formula	m/z
**a**	$[Co_4(L^1)_2(L^2)_4]^{11+}$ $6[PF_6]^-$	942.264
**b**	$[Co_4(L^1)_4(L^2)_2]^{10+}$ $5[PF_6]^-$	950.116
**c**	$[Co_4(L^1)(L^2)_5]^{12+}$ $7[PF_6]^-$	952.834
**d**	$[Co_4(L^1)_3(L^2)_3]^{11+}$ $6[PF_6]^-$	960.686
**e**	$[Co_4(L^1)_5(L^2)]^{10+}$ $5[PF_6]^-$	968.538
**f**	$[Co_4(L^1)_2(L^2)_4]^{12+}$ $7[PF_6]^-$	971.256
**g**	$[Co_4(L^1)_4(L^2)_2]^{11+}$ $6[PF_6]^-$	979.108
**h**	$[Co_4(L^1)_6]^{10+}$ $5[PF_6]^-$	986.960
**i**	$[Co_4(L^1)_3(L^2)_3]^{12+}$ $7[PF_6]^-$	989.678
**j**	$[Co_4(L^1)_5(L^2)]^{11+}$ $6[PF_6]^-$	997.530

Figure S7: ESI-MS of a near equimolar mixture of **1**·12PF$_6$ and **2**·12PF$_6$ after successive heating at 25 °C, 50 °C, 60 °C and 70 °C. Magnified 940-1007 m/z range shown to illustrate mixed ligand species between $[Co_4(L^1)_6]^{12+}$ $7[PF_6]^-$ (m/z = 1044.944) and $[Co_4(L^2)_6]^{12+}$ $7[PF_6]^-$ (m/z = 934.412).

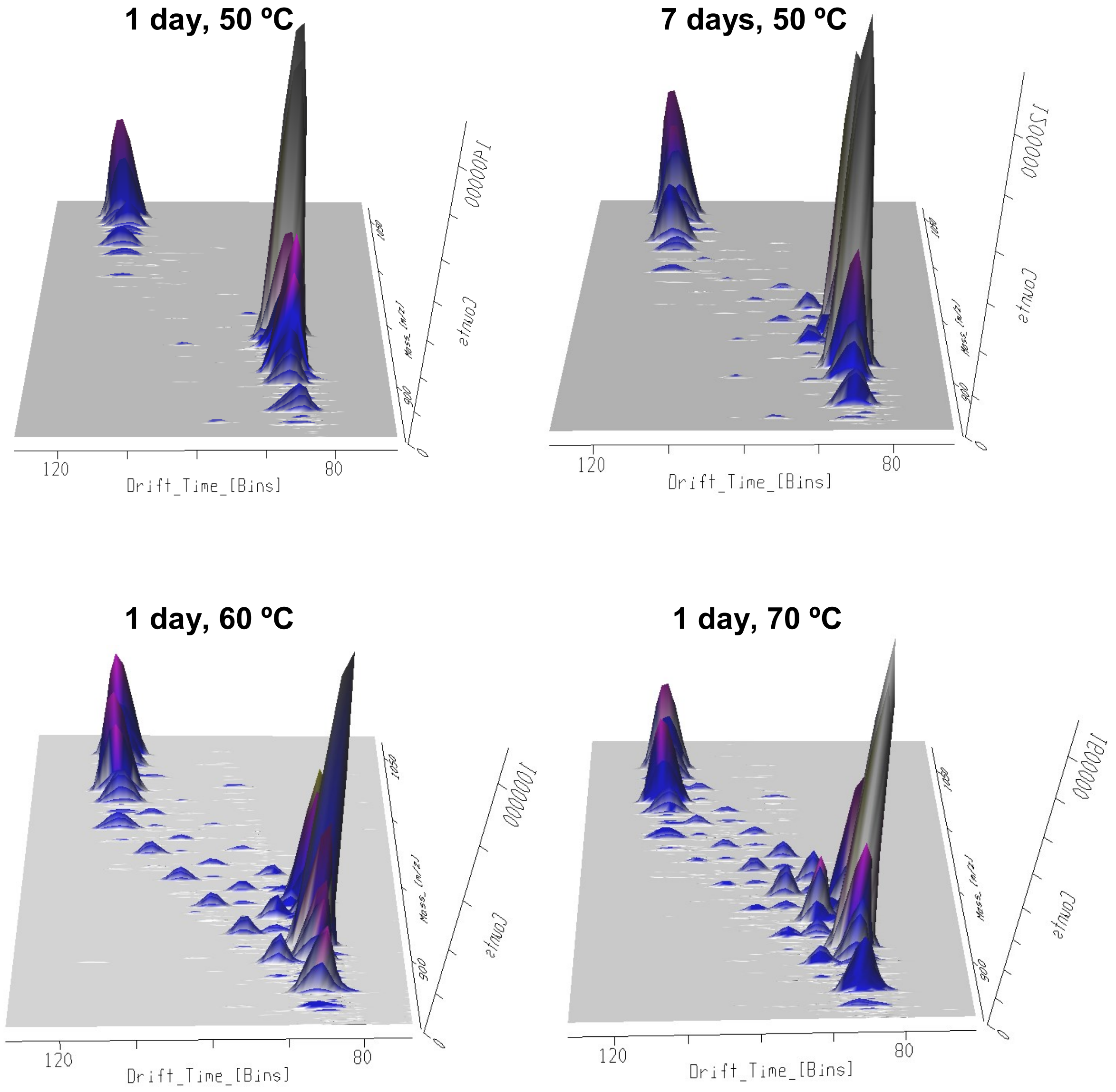

Figure S8: ESI-IM-MS of a near equimolar mixture of **1**·12PF_6 and **2**·12PF_6 over time. Three dimensional ion mobility plots show m/z peaks versus drift time. The larger $[Co_4(L^1)_6]7PF_6$ (DT = ~113 Bins) and smaller $[Co_4(L^2)_6]7PF_6$ (DT = ~86 Bins) are both shown as a series of m/z peaks of equal cross-section due to variation of the counter-ions and Cobalt charge states. After cumulative heating of the sample at increasing temperatures, minor peaks corresponding to the mixed ligand species are observed at intermediate drift times.

5. Cage cavity modelling

In order to help identify guests for these capsules, calculation of the cavity volume in the tetrahedral structures was carried out.[3] The crystal structure data of **1**·12PF_6 superimopsed with the calculated cavity is shown in Figure S9, the volume of which was calculated to be 358 $Å^3$ using a 3.5 Å outer probe and a 0.2 Å inner probe.

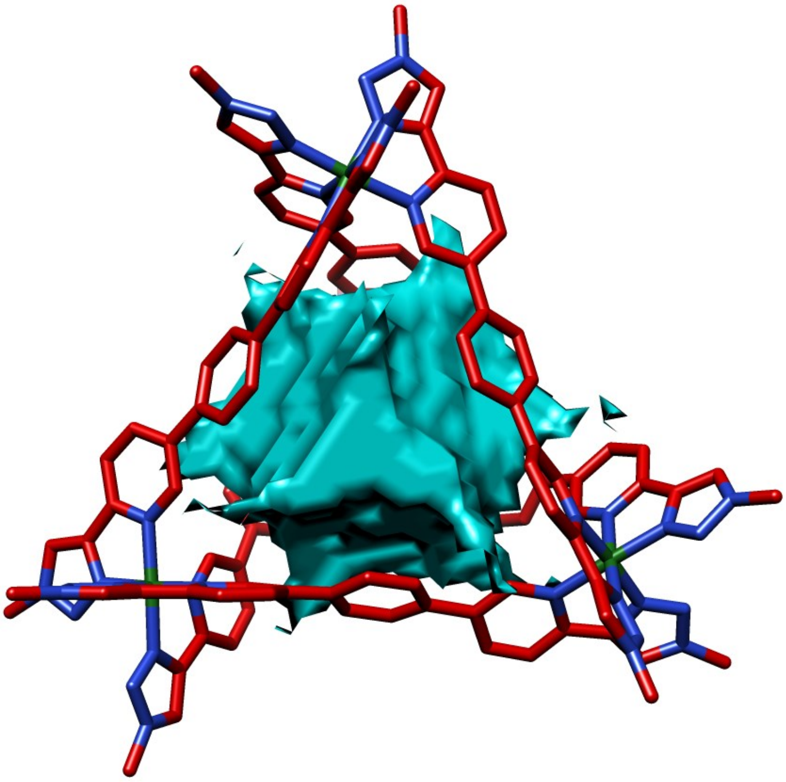

Figure S9 Structure of **1**·12PF_6 derived from crystal structure data with adamantane groups omitted for clarity superimposed with the free cavity space. The internal cavity volume (cyan) was calculated using an outer probe radius of 3.5 Å and an inner probe radius of 0.2Å.

Application of the guidelines for suitable guests[4] indicates that guest volumes of 165 – 229 $Å^3$ are likely to favour encapsulation.

6. Host-guest chemistry

6a. General procedure for identification of guest molecules

To a 2.5 mM D_2O solution of **2**·$12NO_3$ (0.5 mL) in an NMR tube was added *ca.* 15 eq. of prospective guest molecule. Following agitation, the NMR tube was sealed and a ^{1}H NMR spectrum recorded. Molecules were judged to act as a guest if both the signals for $\mathbf{2}^{12+}$ and the molecule in question were shifted in comparison to those species alone. These spectra are stacked in the subsequent section for illustrative purposes.

6b. List of binders for $\mathbf{2}^{12+}$

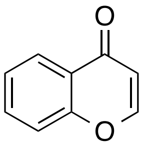

149 A^3

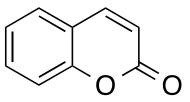

149 A^3

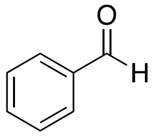

120 A^3

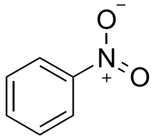

121 A^3

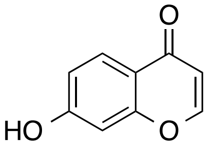

156 A^3

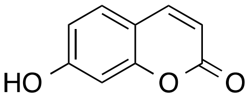

156 A^3

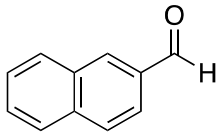

171 A^3

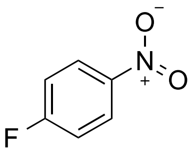

126 A^3

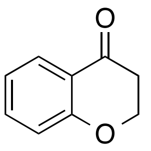

152 A^3

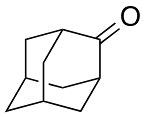

162 A^3

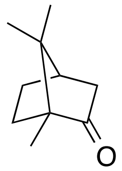

176 A^3

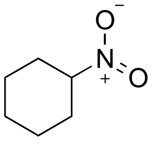

134 A^3

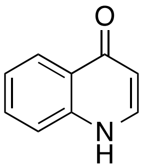

152 A^3

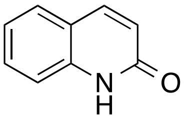

152 A^3

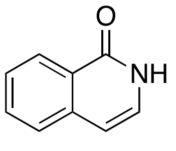

151 A^3

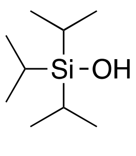

221 A^3

6c. List of non-binders for 2^{12+}

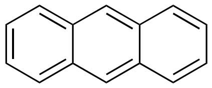

201 A^3

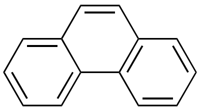

202 A^3

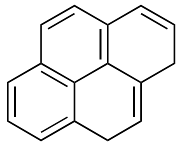

219 A^3

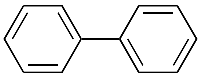

183 A^3

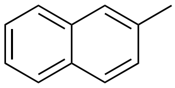

168 A^3

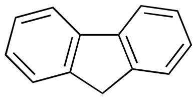

189 A^3

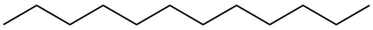

235 A^3

6d. NMR stackplots for host-guest system

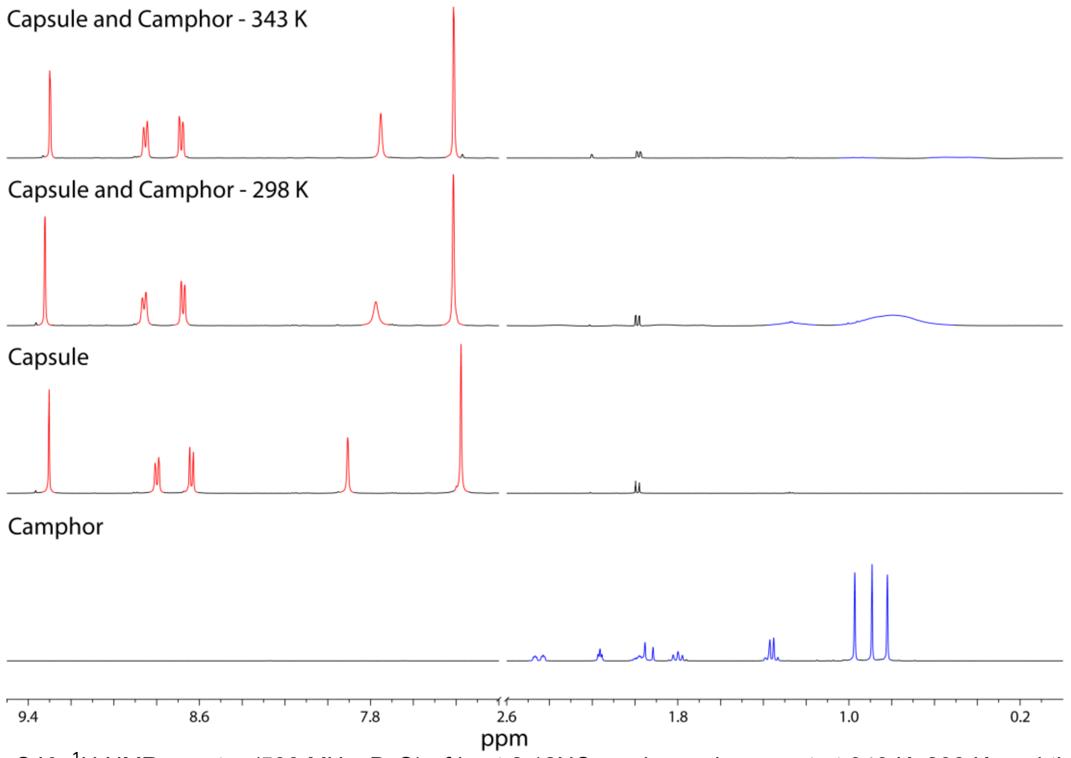

Figure S10: ^{1}H NMR spectra (500 MHz, D_2O) of host **2**·12NO_3 and camphor guest at 343 K, 298 K, and then the separate host and guest species.

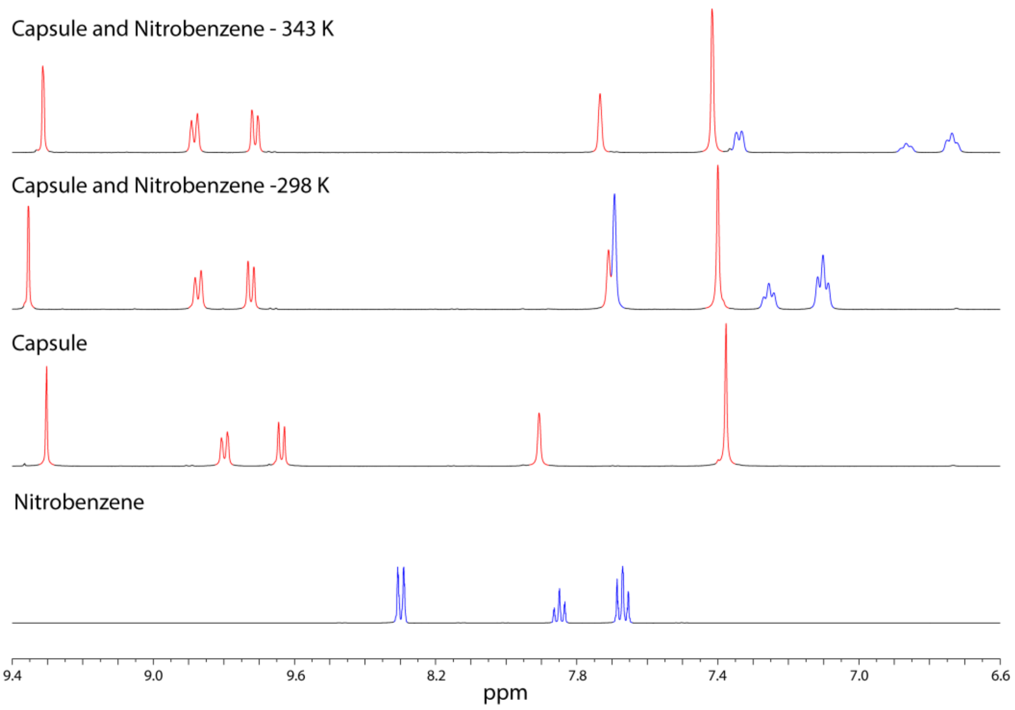

Figure S11: ^{1}H NMR spectra (500 MHz, D_2O) of host **2**·12NO_3 and nitrobenzene guest at 343 K, 298 K, and then the separate host and guest species.

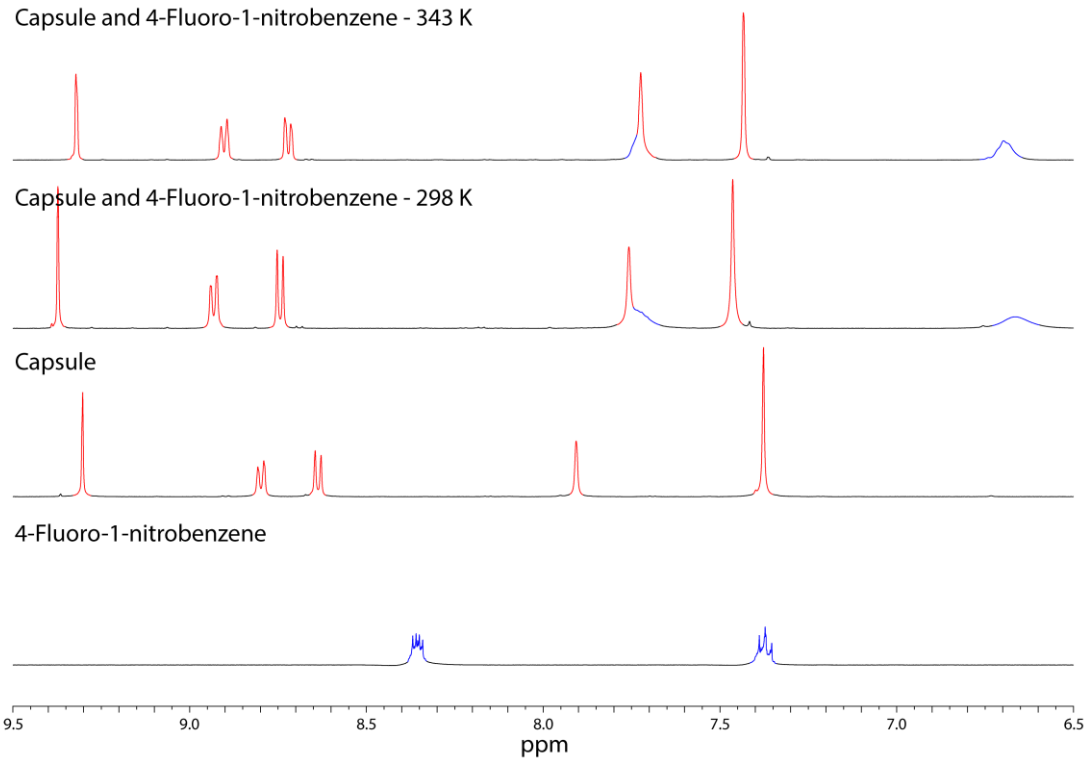

Figure S12: ^{1}H NMR spectra (500 MHz, D_2O) of host **2**·$12NO_3$ and 4-fluoro-1-nitrobenzene guest at 343 K, 298 K, and then the separate host and guest species.

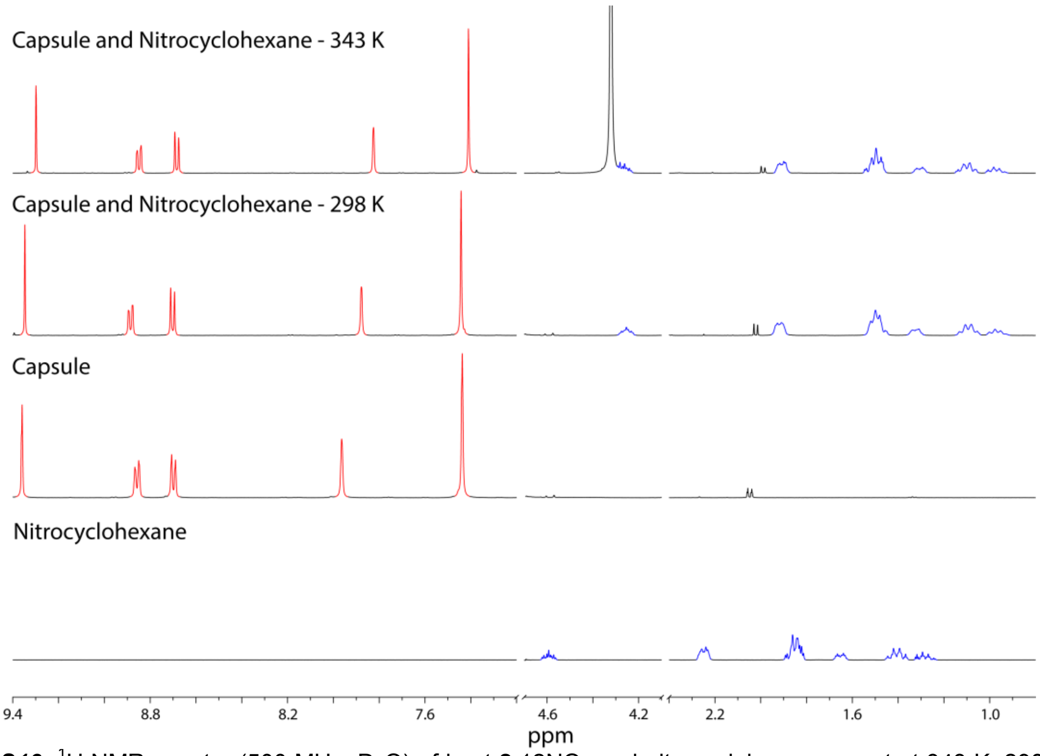

Figure S13: ^{1}H NMR spectra (500 MHz, D_2O) of host **2**·$12NO_3$ and nitrocyclohexane guest at 343 K, 298 K, and then the separate host and guest species.

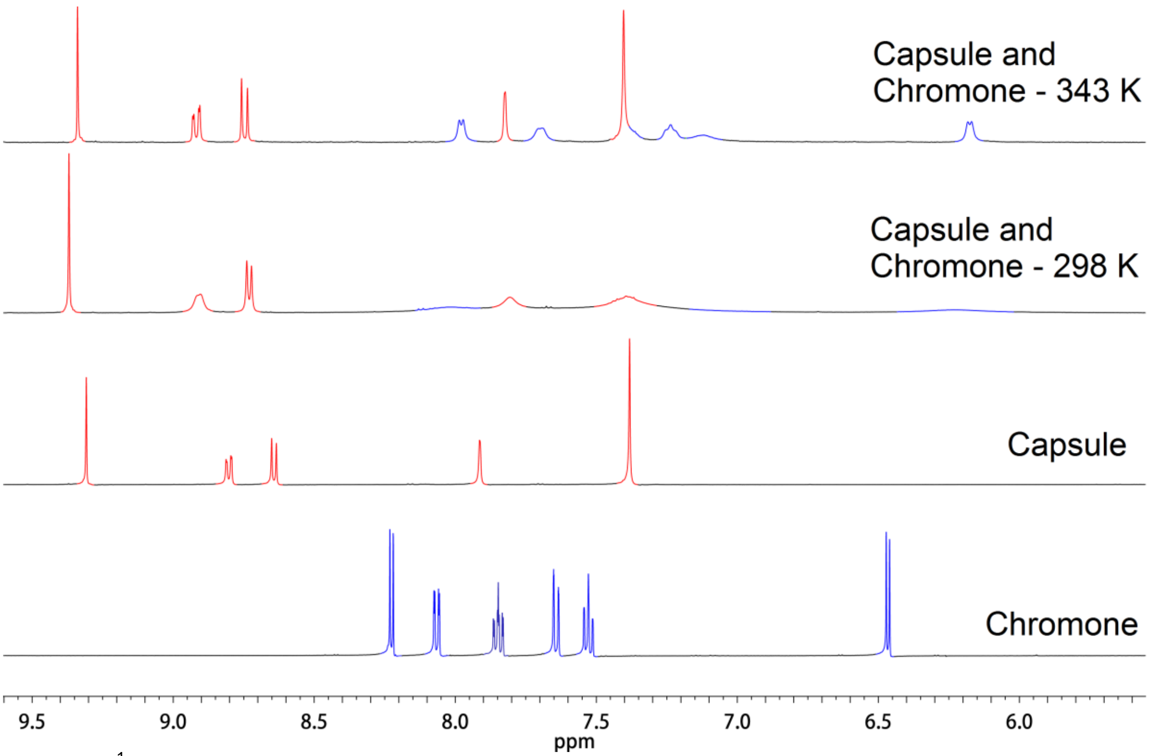

Figure S14: ^{1}H NMR spectra (500 MHz, D_2O) of host **2**·12NO_3 and chromone guest at 343 K, 298 K, and then the separate host and guest species.

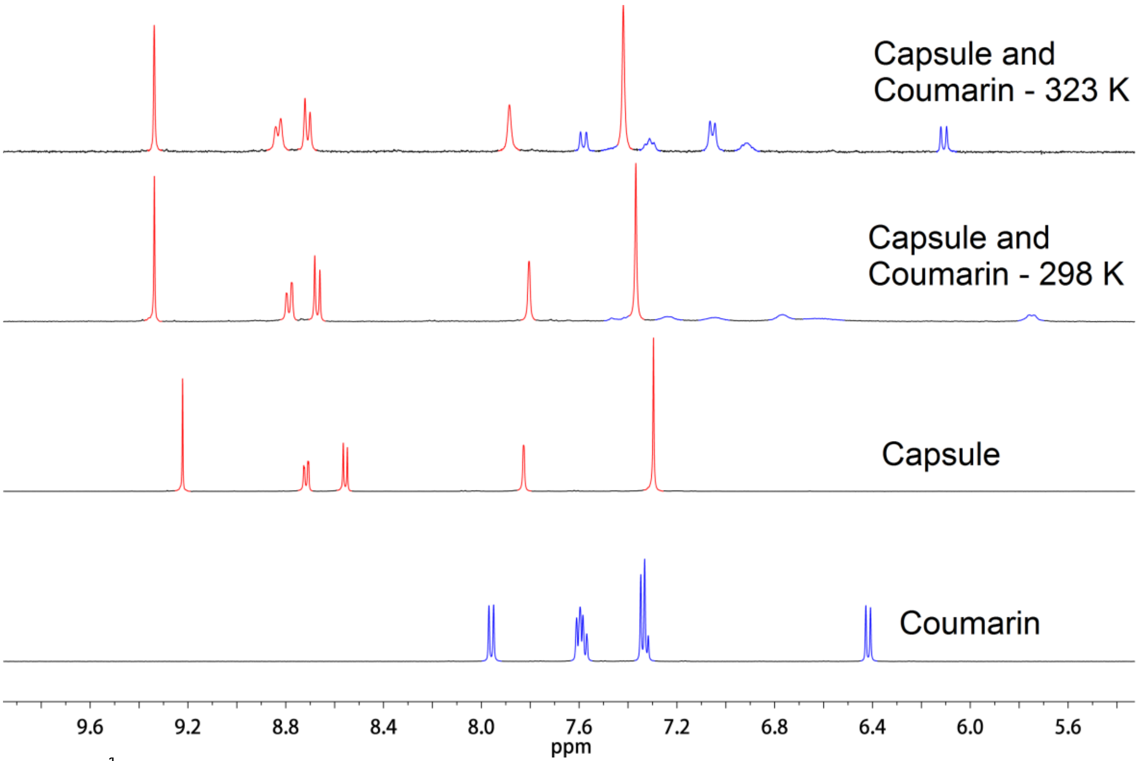

Figure S15: ^{1}H NMR spectra (500 MHz, D_2O) of host **2**·12NO_3 and coumarin guest at 343 K, 298 K, and then the separate host and guest species.

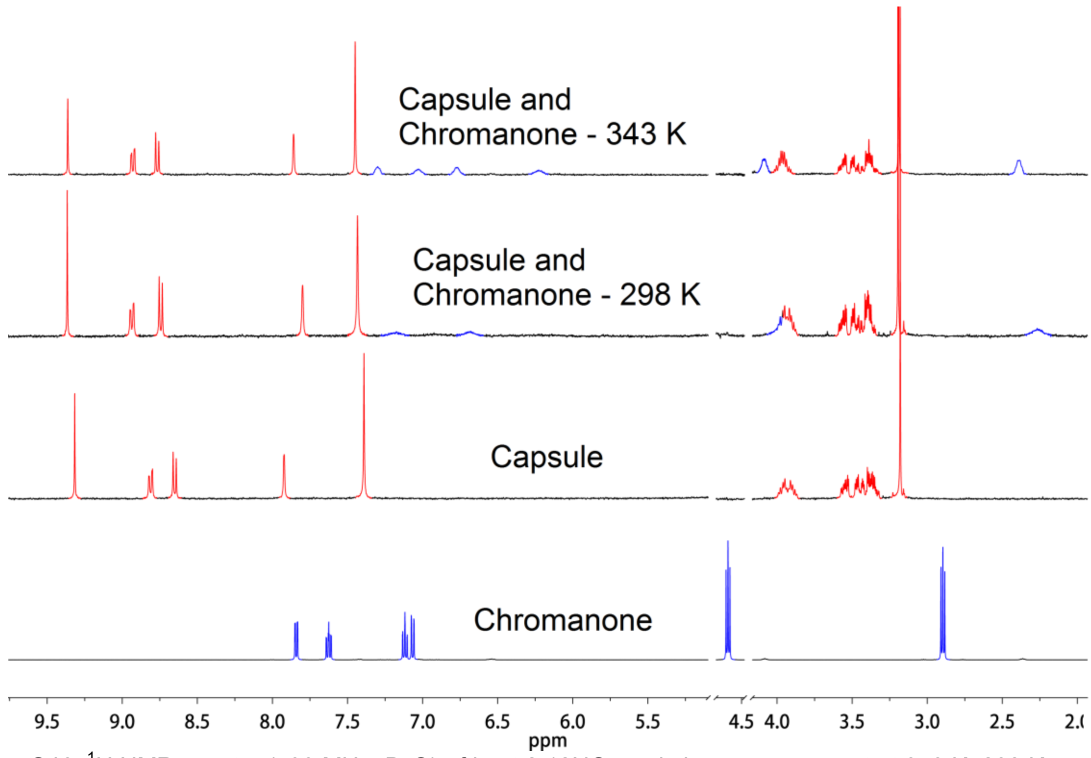

Figure S16: ^{1}H NMR spectra (500 MHz, D_2O) of host **2**·$12NO_3$ and chromanone guest at 343 K, 298 K, and then the separate host and guest species.

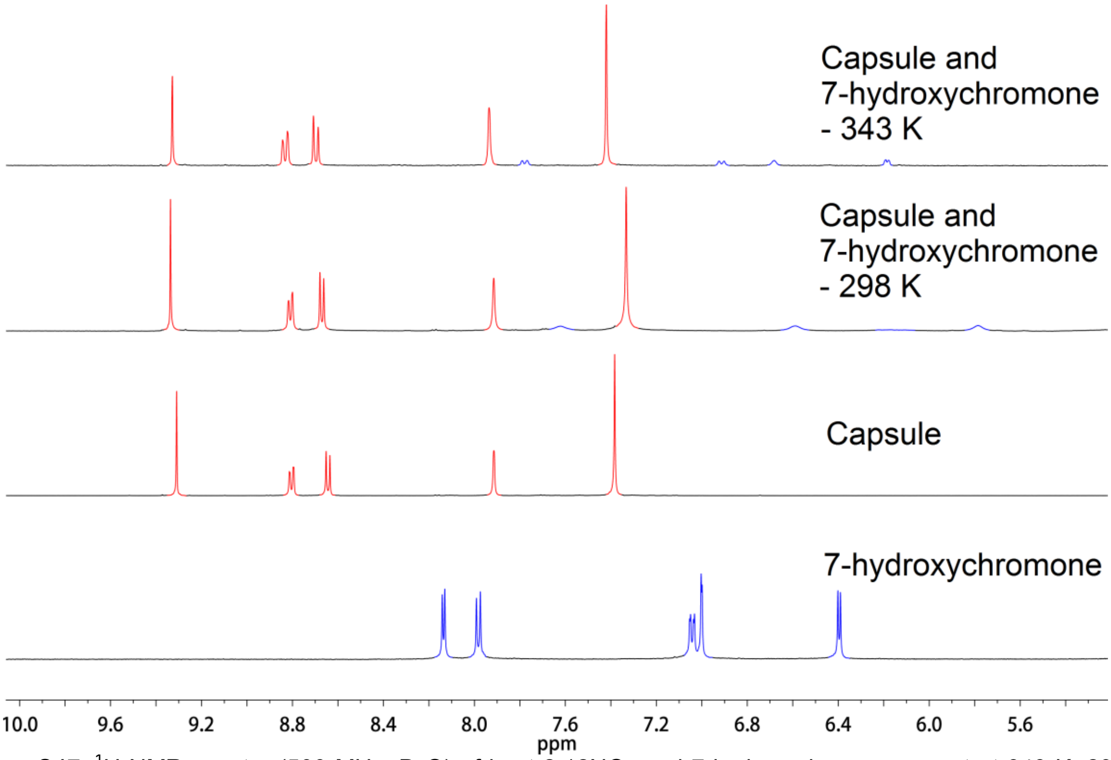

Figure S17: ^{1}H NMR spectra (500 MHz, D_2O) of host **2**·$12NO_3$ and 7-hydroxychromone guest at 343 K, 298 K, and then the separate host and guest species.

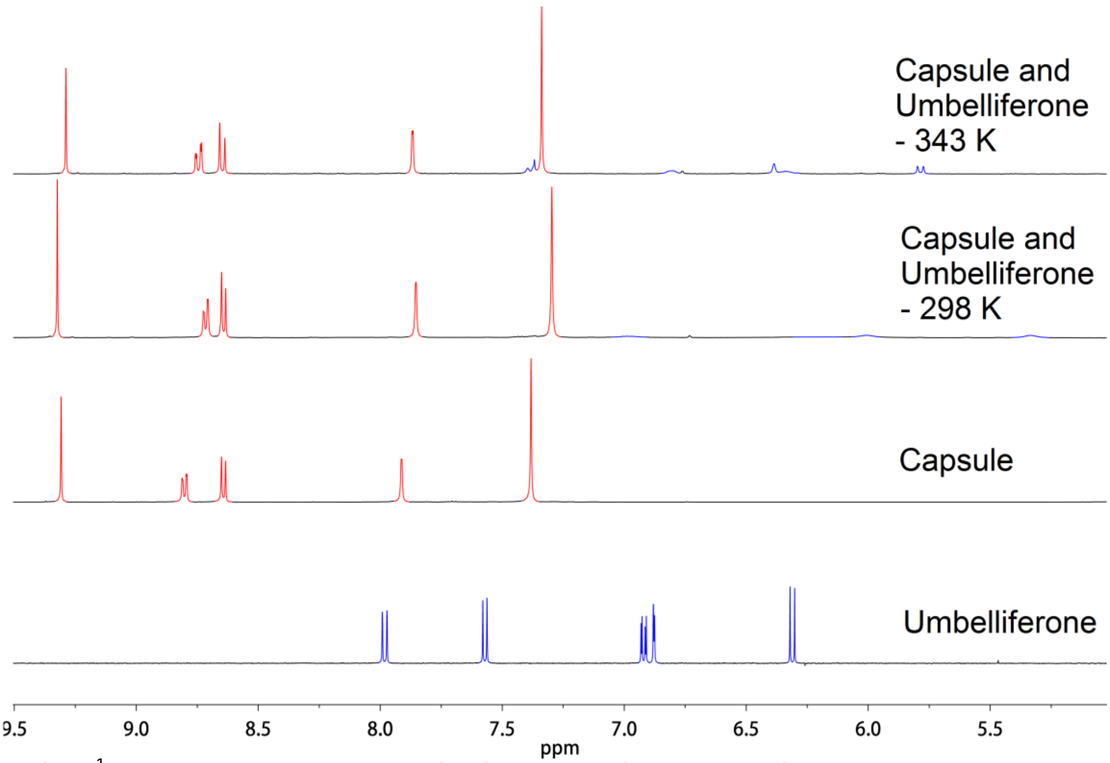

Figure S18: ^{1}H NMR spectra (500 MHz, D_2O) of host **2**·$12NO_3$ and umbelliferone guest at 343 K, 298 K, and then the separate host and guest species.

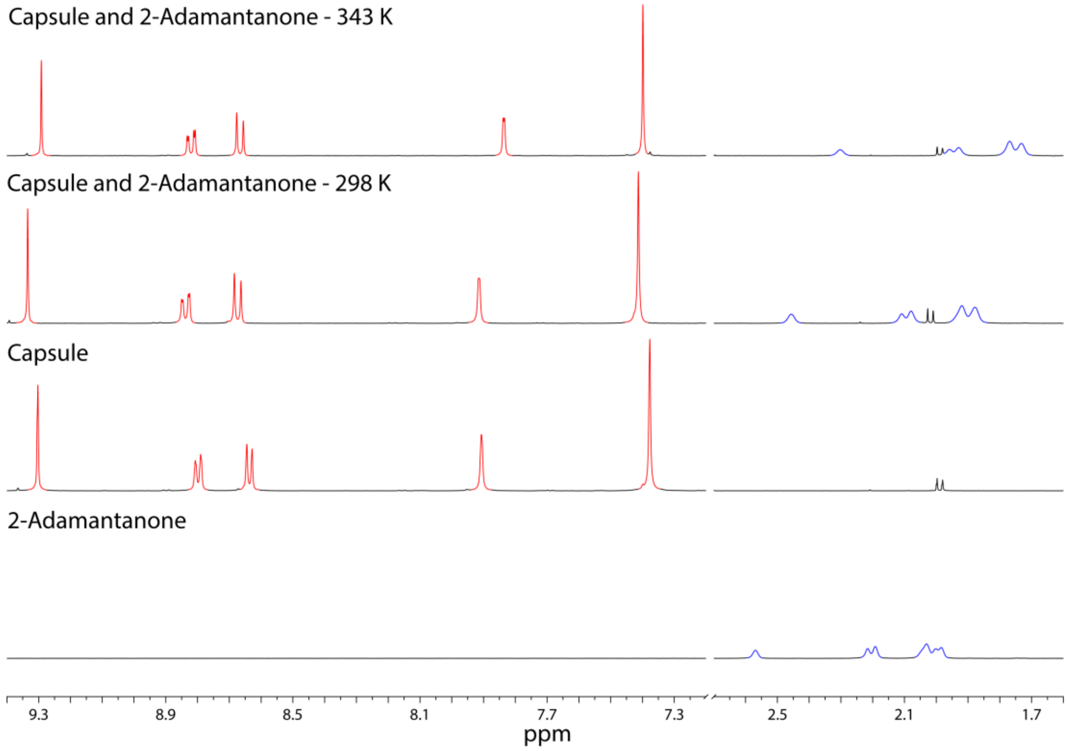

Figure S19: ^{1}H NMR spectra (500 MHz, D_2O) of host **2**·$12NO_3$ and 2-adamantanone guest at 343 K, 298 K, and then the separate host and guest species.

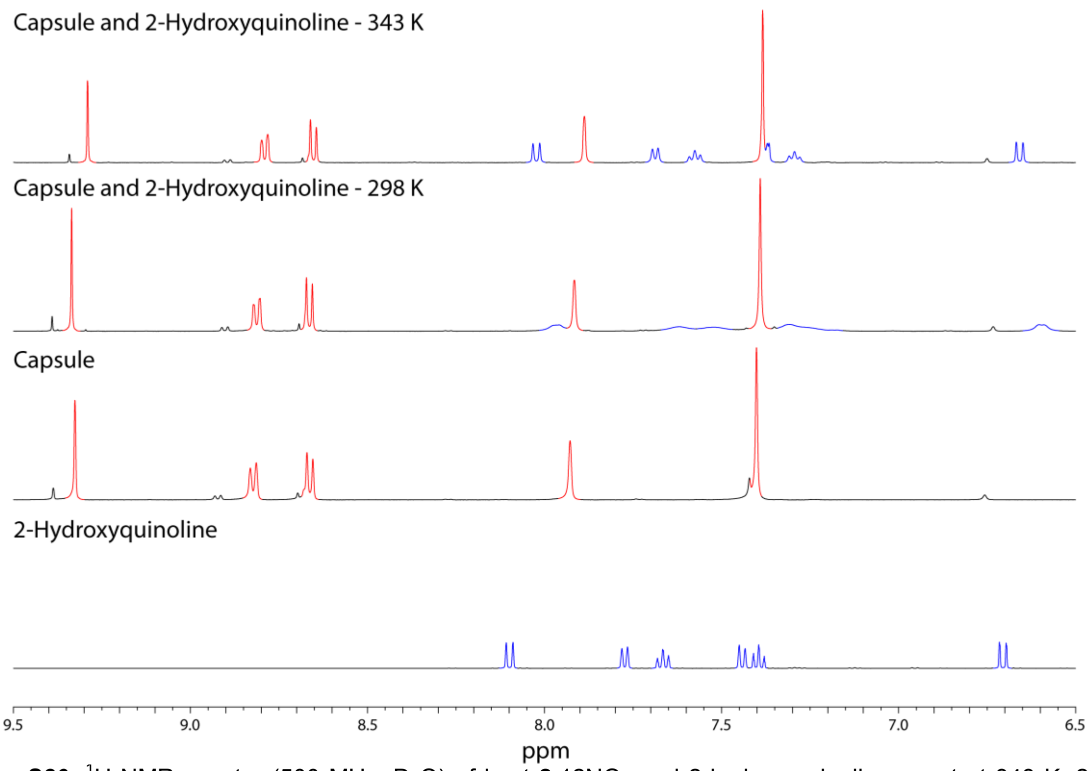

Figure S20: ^{1}H NMR spectra (500 MHz, D_2O) of host **2**·12NO_3 and 2-hydroxyquinoline guest at 343 K, 298 K, and then the separate host and guest species.

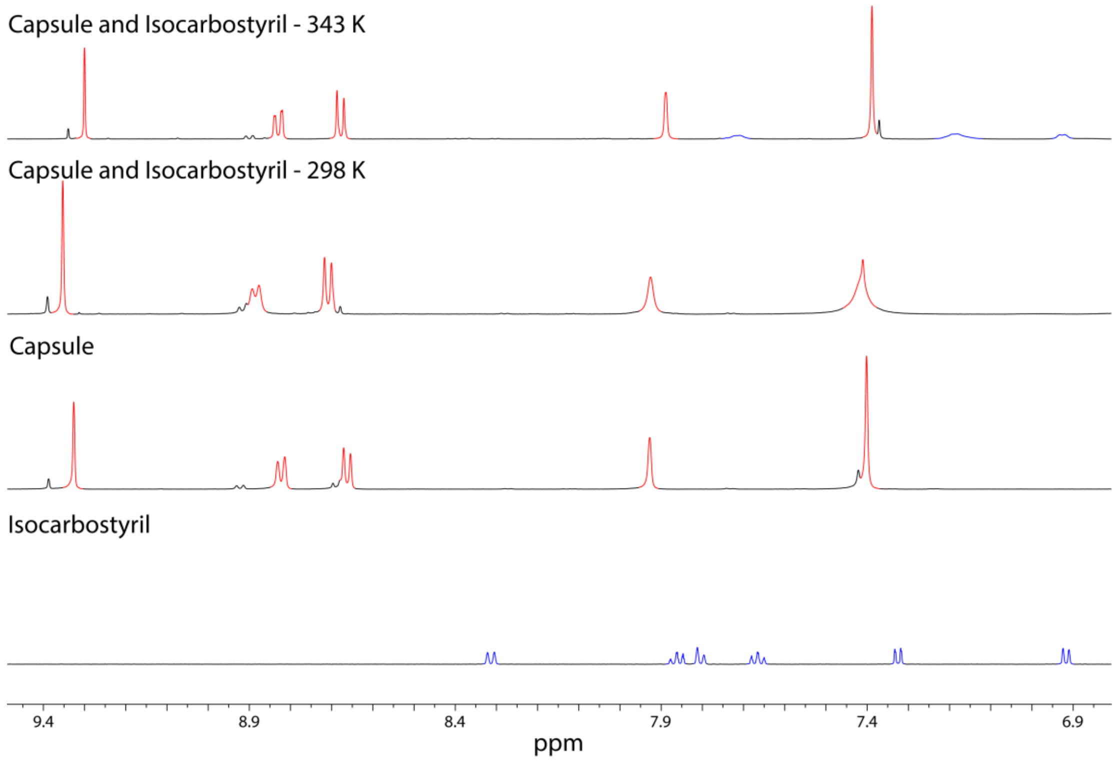

Figure S21: ^{1}H NMR spectra (500 MHz, D_2O) of host **2**·12NO_3 and isocarbostyril guest at 343 K, 298 K, and then the separate host and guest species.

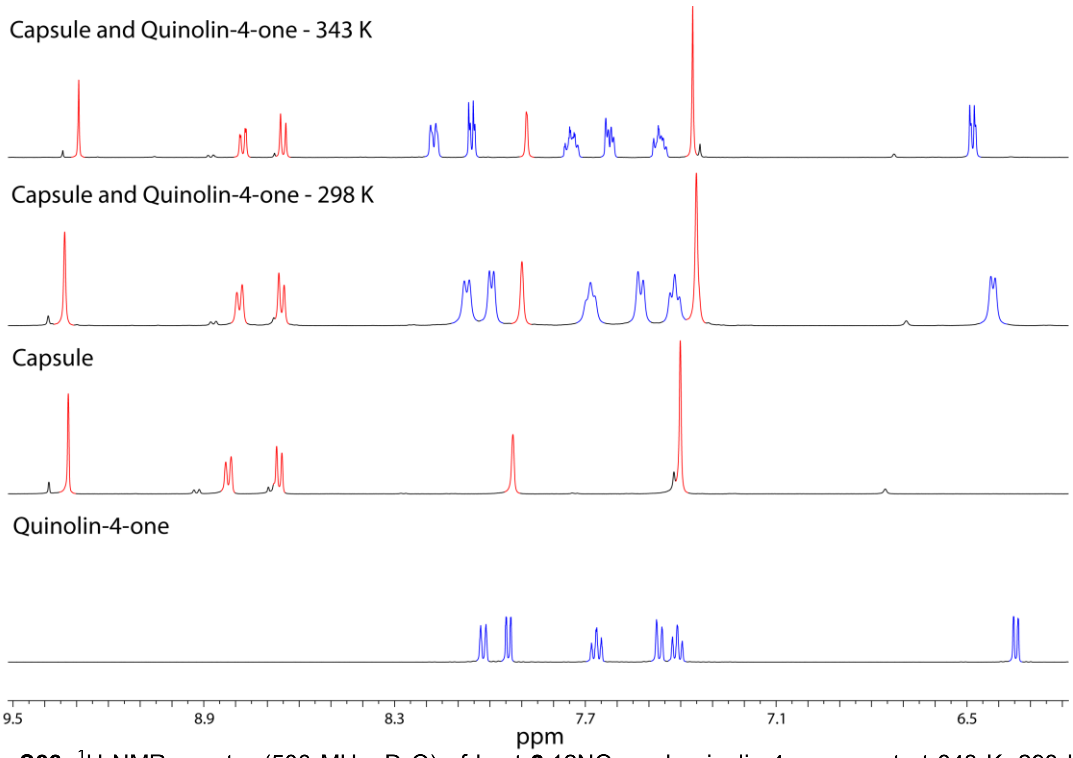

Figure S22: ^{1}H NMR spectra (500 MHz, D_2O) of host **2**·$12NO_3$ and quinolin-4-one guest at 343 K, 298 K, and then the separate host and guest species.

6e. NMR studies of TIPSOH encapsulation.

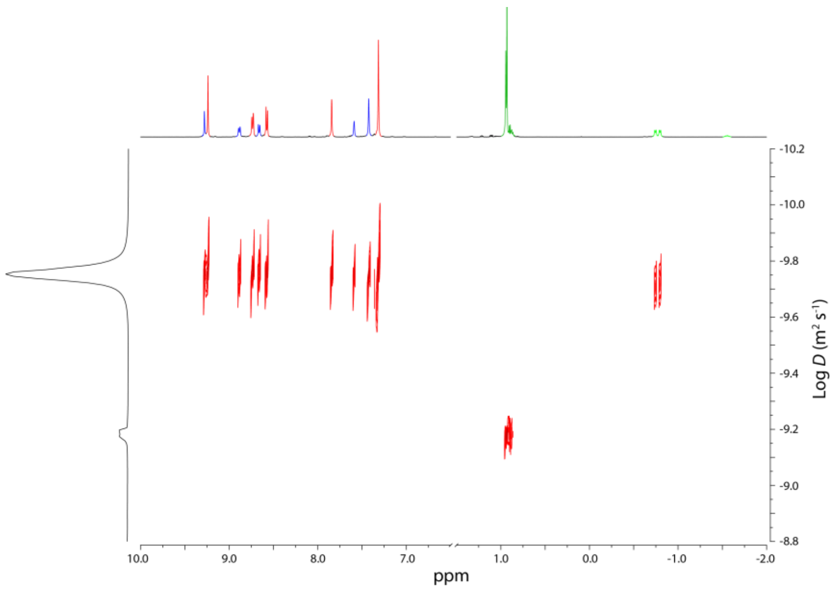

Figure S23: ^{1}H NMR DOSY (400 MHz, D_2O) spectrum of host **2**·$12NO_3$ and in the presence of excess TIPSOH.

6f. K_a determination for guests in NMR slow exchange

To a solution of **2**·$12NO_3$ in D_2O (2.5 mM, 0.5 mL) was added an excess of triisopropylsilanol (TIPSOH). This was thoroughly mixed and then sonicated for 0.25 h and then left overnight to equilibrate, after which the sample was centrifuged and the organic layer decanted. ^1H NMR spectra were recorded and integration used to calculate the molar fraction of free to occupied host and the relative concentration of TIPSOH guest. These measurements were repeated for varying concentrations of $NaNO_3$ (0, 0.1, 1 and 5 M).

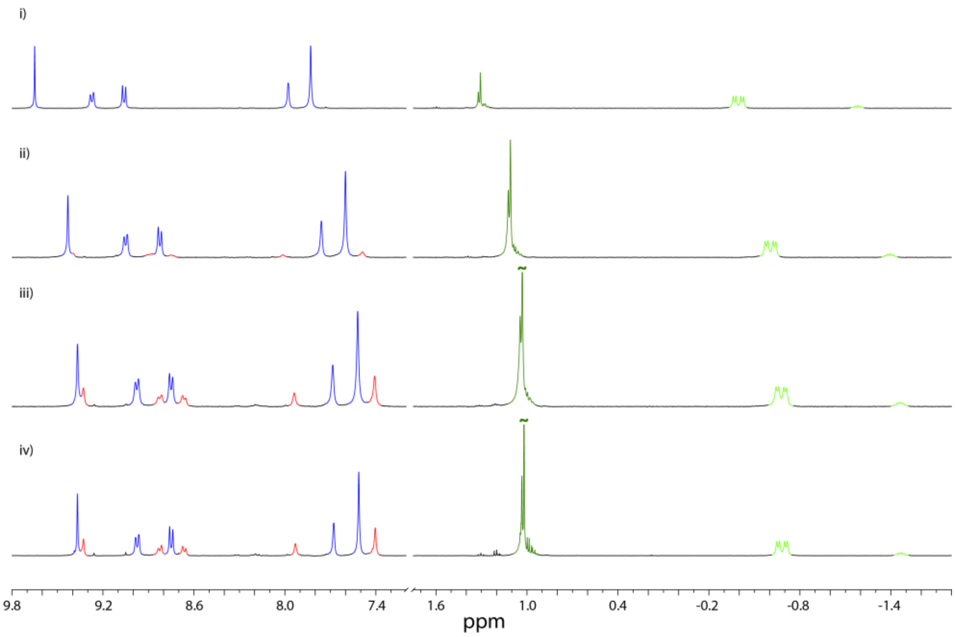

Figure S24: ^1H NMR spectra (400 MHz, D_2O) of host **2**·$12NO_3$ and TIPSOH at i) 5.0 M $NaNO_3$, ii) 1.0 M $NaNO_3$, iii) 0.1 M $NaNO_3$ and iv) with no added salt, all at 298 K. The free guest is shown in dark green and the bound in light green, with bound-cage complex in blue and free-cage in red.

K_a was calculated using the following equation.

$$K_a = \frac{(1-\chi_{eq})[H_0]}{\chi_{eq}[H_0][G_{eq}]}$$

$[NaNO_3]$ (M)	0	0.1	1.0	5.0
K_a (M^{-1})	1410	1770	3430	4970

Table S25: The K_a values derived from ^1H NMR spectra of host **2**·$12NO_3$ with saturated TIPSOH at varying salt concentrations. The value for 5.0 M $NaNO_3$ was achieved by dilution of **2**·$12NO_3$ and TIPSOH (but not salt concentration as the peaks for free **2**·$12NO_3$ were not detectable by NMR).

6g. EXSY NMR experiments

To a solution of **2**·12NO_3 in D_2O (2.5 mM, 0.5 mL) was added an excess of triisopropylsilanol (TIPSOH). This was thoroughly mixed and then sonicated for 0.25 h, after which the sample was centrifuged and the organic layer decanted. ^{1}H NMR NOESY spectra were recorded with different mixing times to obtain a series of EXSY spectra, from which the ratio of the cross-peaks by integration between the free and bound TIPSOH were analysed to give the observed rate of ingress/egress of guest into and out of the cavity. These measurements were repeated for varying concentrations of $NaNO_3$ (0, 0.1 and 1 M).

The change in ratio of the diagonal to cross-peaks as a function of time allows for the fitting of a linear regression, from which the $\Delta G^{\ddagger}$ can be ascertained by the following equations, reported by Macchioni *et al.*[5]

$$r = \frac{(I_{AA} + I_{BB})}{(I_{AB} + I_{BA})}$$

$$HG_n + G^* \underset{k_{-1}}{\overset{k_1}{\rightleftharpoons}} HG_{n-1}G^* + G$$

$$k = \frac{1}{\tau_m} \times \ln\frac{r+1}{r-1}$$

$$k = k_1 + k_{-1} = 2 \times k_{obs} \quad (k_1 = k_{-1} = k_{obs})$$

$$\Delta G^{\ddagger} = -RT\ln\frac{k_{obs}h}{k_B T}$$

$$I_{AA}\ \&\ I_{BB} = diagonal\ peak\ intensities$$

$$I_{AB}\ \&\ I_{BA} = cross - peak\ intensities$$

$$\tau_m = mixing\ time\ in\ s$$

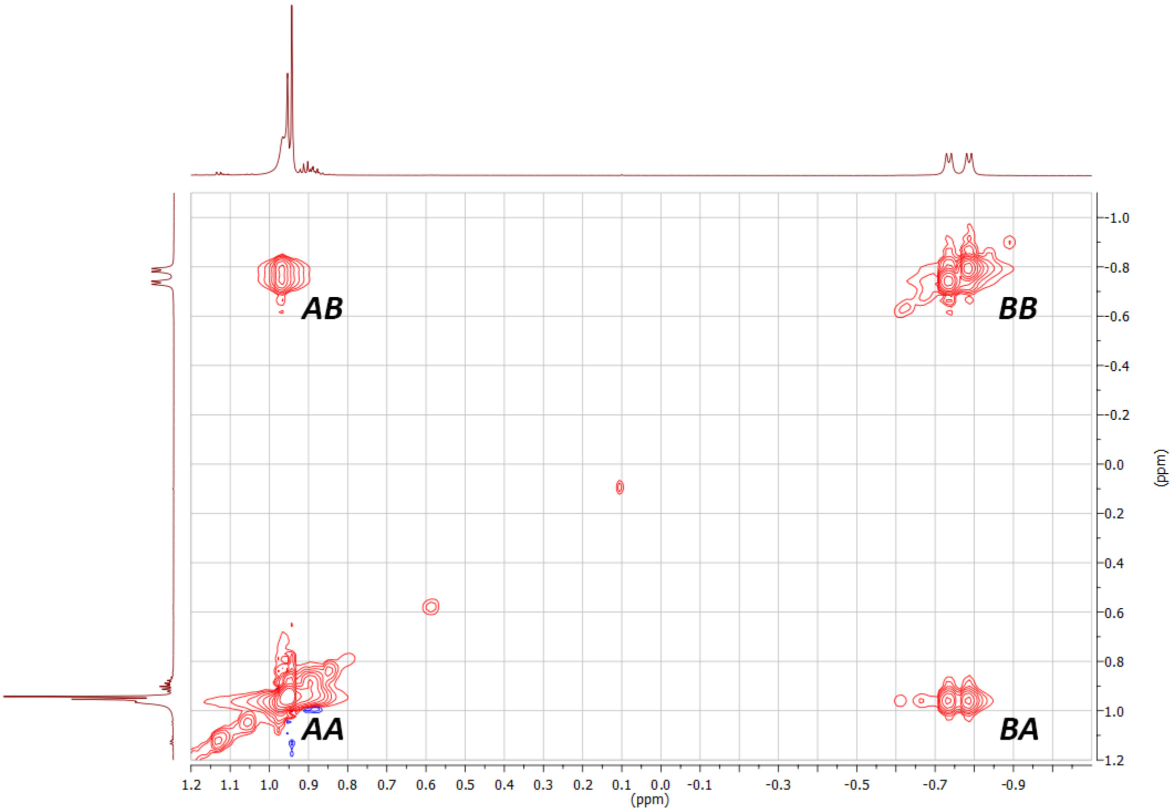

Figure S26: A representative ^{1}H NOESY NMR spectrum (600 MHz, D_2O) of **2**·12NO_3 with triisopropylsilanol guest used in the EXSY experiment showing the diagonal peaks (AA and BB) and cross-peaks (AB and BA).

I_{AA}	I_{BB}	I_{AB}	I_{BA}	r	ln(r+1/r-1)	τ_m (s)
24.0789	10.4179	1.0000	1.1172	16.2936	0.1229	0.026
13.0443	5.2406	1.0000	1.0581	8.8844	0.2261	0.056
9.7239	3.6541	1.0000	1.0432	6.5475	0.3079	0.078
7.9643	2.8069	1.0000	1.0236	5.3228	0.3803	0.104

Table S27: The ratio of bound to unbound guest peaks by integration of ^{1}H NOESY NMR spectrum (600 MHz, D_2O) of **2**·12NO_3 with TIPSOH as a function of mixing time.

k_{obs} (s^{-1})	1.673
$\Delta G^{\ddagger}$ (kcal mol^{-1})	17.26

Table S28: The kinetic parameters for the exchange of TIPSOH in and out of **2**·12NO_3.

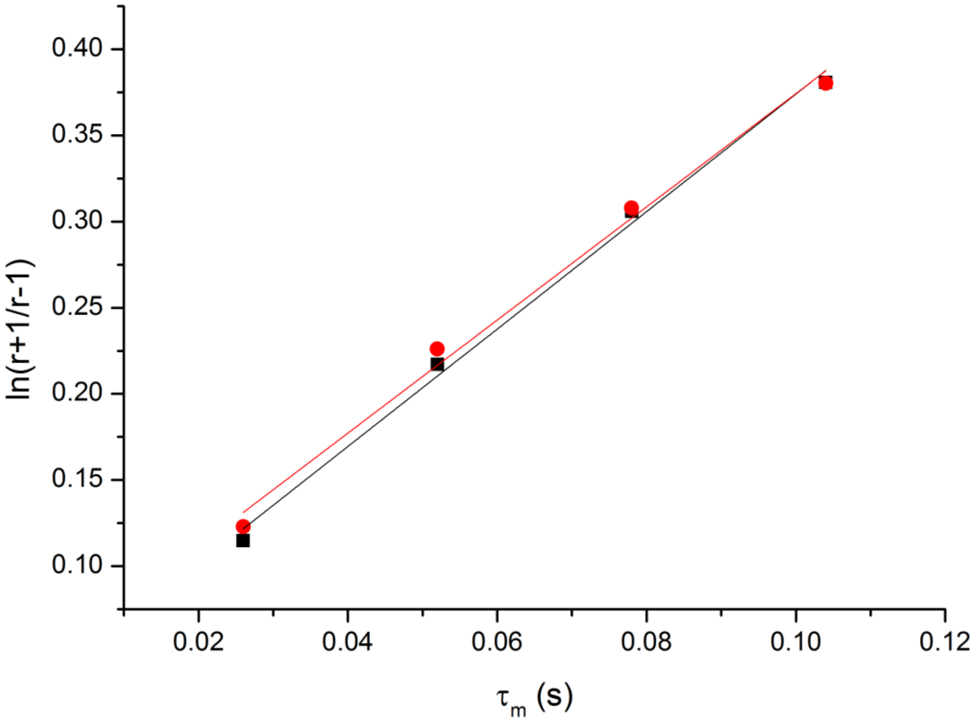

Figure S29: Fitted lines of integrated peak ratios vs mixing time from ^{1}H EXSY NMR spectra (600 MHz, D_2O) of **2**·12NO_3 with TIPSOH. Red and black lines are duplicate experiments.

6h. K_a determination for selected guests in NMR fast exchange

General procedure: A solution of **2**.12NO_3 in D_2O (2.5 mM, 0.5 mL) had excess guest added to it, and the mixture was sonicated for 0.25 h, then heated at 50 °C for ~18 h. The resulting saturated solution was filtered to remove the remaining undissolved guest. This was titrated in (typically) 10 μL portions to a sample of the original solution of **2**.12NO_3 without saturated guest. Between each addition, the sample was well mixed and the ^1H NMR spectrum (400 MHz) was re-recorded. After the addition of (typically) 300 μL of guest-saturated solution titration was stopped unless otherwise stated. All experiments were carried out at 298 K.

Titration in this manner allowed monitoring of the shifts of the host peaks as a function of guest concentration, to which a curve can then be fitted in the manner described extensively by Hristova *et al.*[6] This used non-linear regression with OriginPro 8 to fit the equation below, which allows determination of K_a as a function of the curve. During the regression, only the total host concentration value was constrained (at 2.5 mM).

$$y = y_0 + \Delta y \left(\frac{\left(1 + K_a(P + x)\right) - \sqrt{(K_a(P + x) + 1)^2 - 4K_a{}^2 Px}}{2K_a x} \right)$$

(where y = chemical shift; y0 = chemical shift with no guest present; Δy = maximum change in chemical shift; P = total host concentration; x = guest concentration; Ka = binding constant)

The fitted curves and values for all guests measured are shown below in Figures S30-37 and Table S38. The value for host concentration was fixed at 2.5 mM. The host signals monitored were chosen on the basis of clear shifts between the encapsulating and free states (to minimise error), with the proviso that the peaks did not exhibit noticeable broadening. Guest concentration was calculated by ^1H NMR integration of the saturated solution after filtering by comparing against the host resonances, for which the concentration was known (2.5 mM).

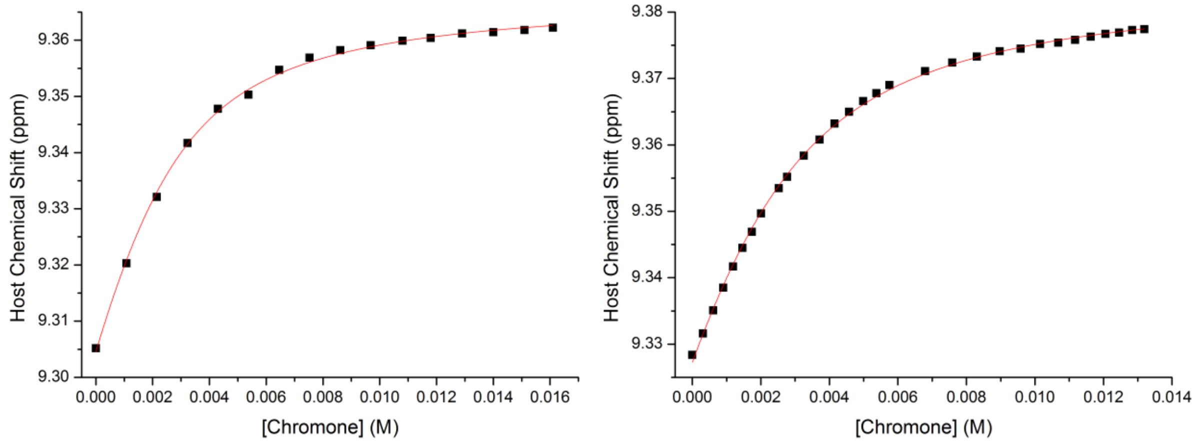

Figure S30: Fitted curves of host chemical shift of H_A against guest concentration for titration of chromone into a fixed concentration of **2**·12NO$_3$ (duplicate experiments).

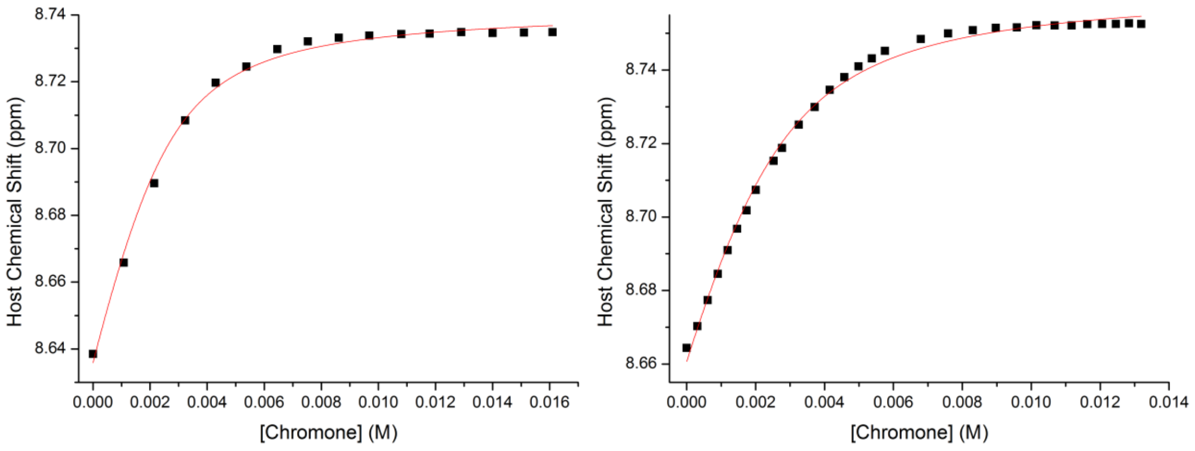

Figure S31: Fitted curves of host chemical shift of H_B against guest concentration for titration of chromone into a fixed concentration of **2**·12NO$_3$ (duplicate experiments).

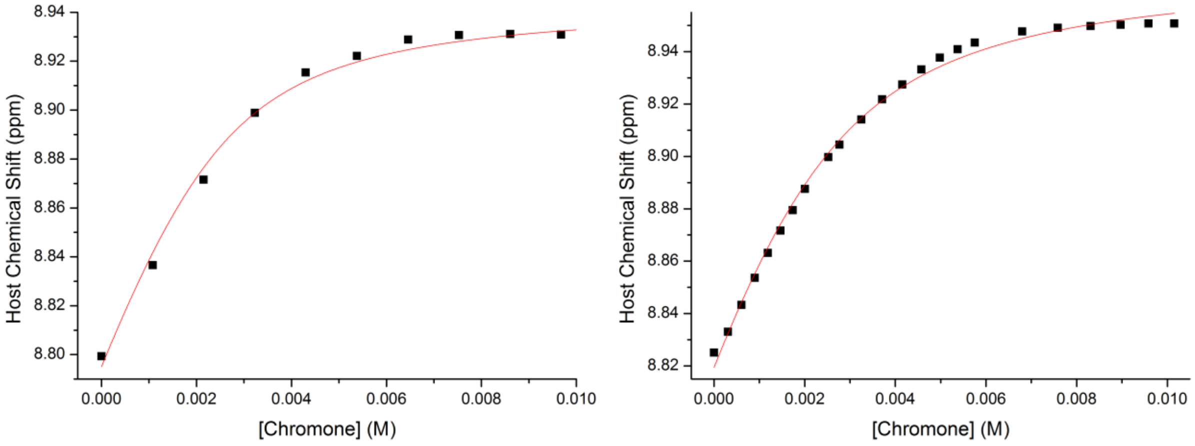

Figure S32: Fitted curves of host chemical shift of H_C against guest concentration for titration of chromone into a fixed concentration of **2**·12NO_3 (duplicate experiments).

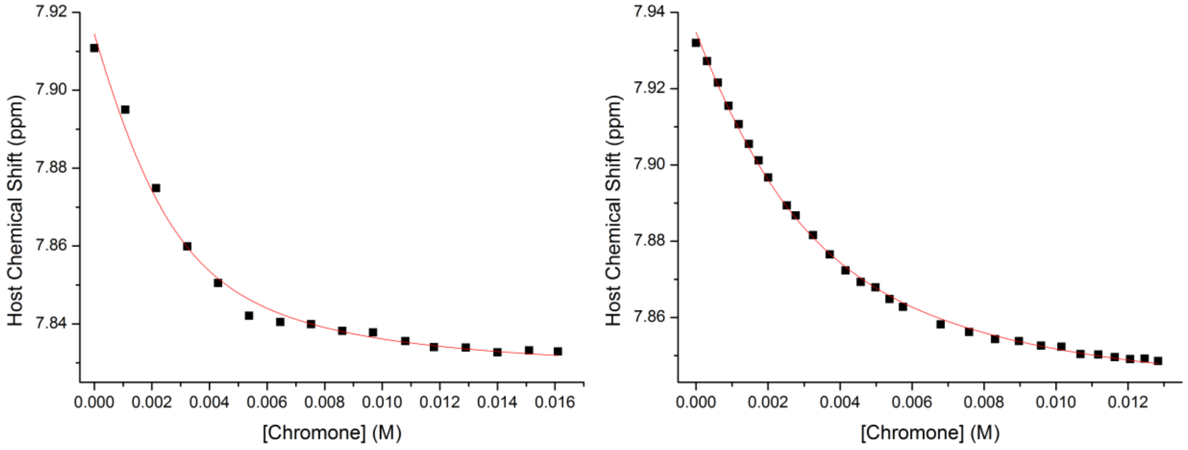

Figure S33: Fitted curves of host chemical shift of H_D against guest concentration for titration of chromone into a fixed concentration of **2**·12NO_3 (duplicate experiments).

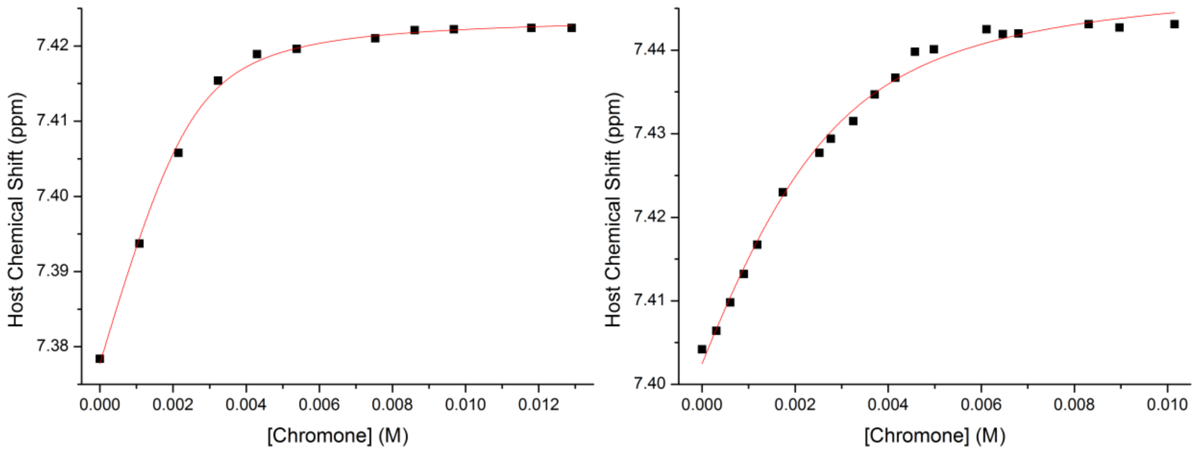

Figure S34: Fitted curves of host chemical shift of H_E against guest concentration for titration of chromone into a fixed concentration of **2**·12NO_3 (duplicate experiments).

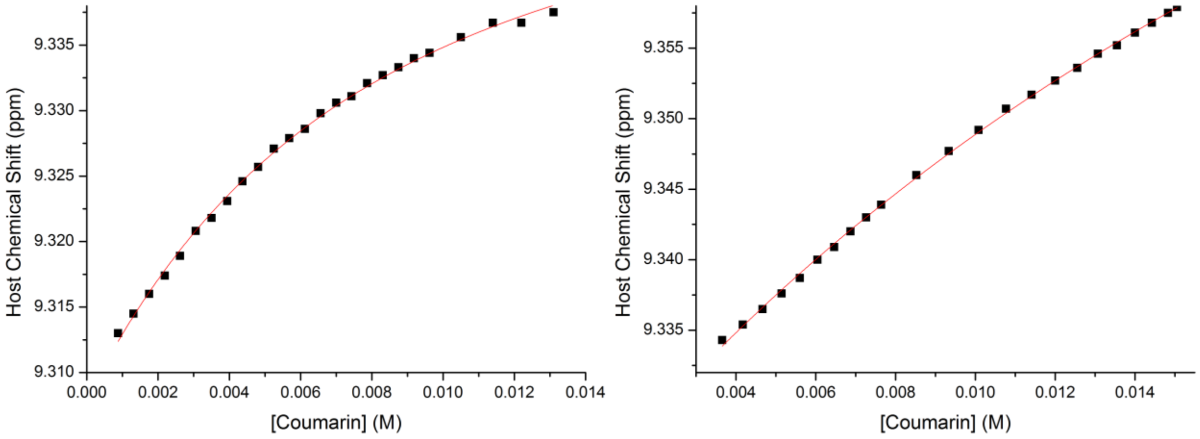

Figure S35: Fitted curves of host chemical shift of H_A against guest concentration for titration of coumarin into a fixed concentration of **2**·12NO_3 (duplicate experiments).

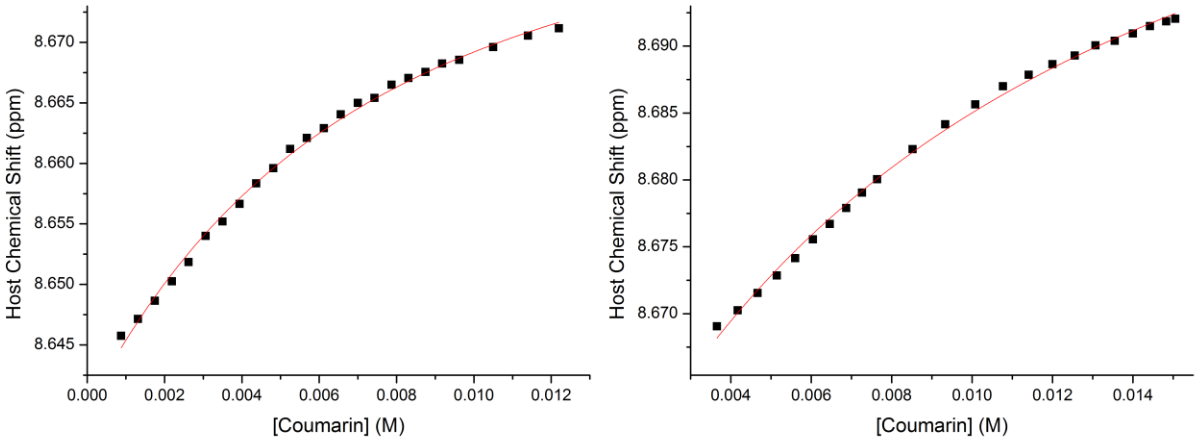

Figure S36: Fitted curves of host chemical shift of H_B against guest concentration for titration of coumarin into a fixed concentration of **2**·12NO_3 (duplicate experiments).

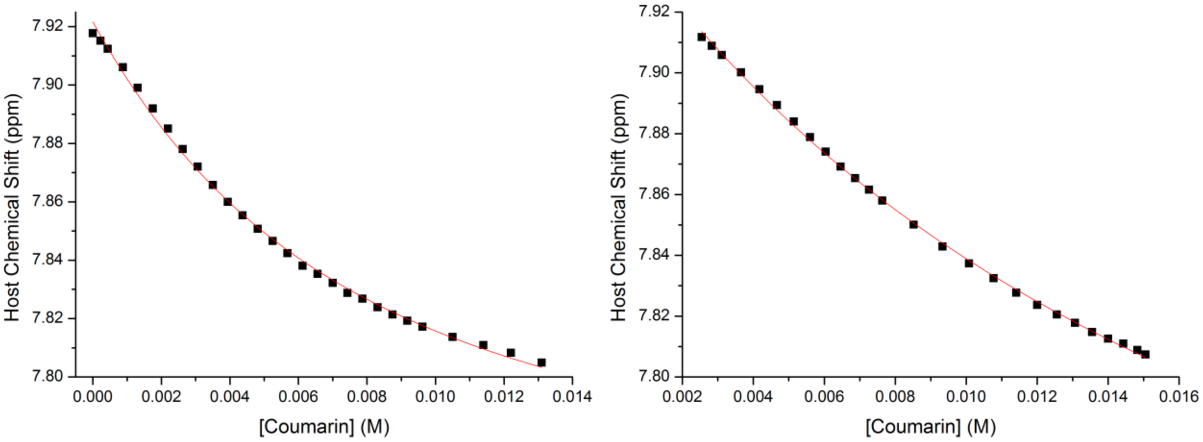

Figure S37: Fitted curves of host chemical shift of H_D against guest concentration for titration of coumarin into a fixed concentration of **2**·12NO_3 (duplicate experiments).

Guest	Cage Peak	Initial Host Shift y0 /ppm		Change in Host Shift DY /ppm		Association Constant K_a /M^{-1}		Adjusted R^2	
		1st Run	2nd Run	1st Run	2nd Run	1st Run	2nd Run	1st Run	2nd Run
Chromone	A	9.305	9.327	0.063	0.057	792 ± 42	632 ± 19	0.999	0.999
	B	8.635	8.661	0.106	0.001	1466 ± 171	1082 ± 80	0.995	0.996
	C	8.795	8.819	0.15	0.153	1511 ± 287	982 ± 100	0.992	0.996
	D	7.914	7.935	-0.089	-0.1	951 ± 144	605 ± 27	0.99	0.999
	E	7.377	7.402	0.046	0.047	3047 ± 427	1152 ± 180	0.997	0.993
Coumarin	A	9.308	9.323	0.046	0.104	162 ± 11	36 ± 4	0.998	0.999
	B	8.64	8.651	0.048	0.069	183 ± 18	114 ± 15	0.997	0.997
	D	7.922	7.951	-0.18	-0.321	167 ± 11	59 ± 4	0.998	0.999

Table S38: Fast exchange guest fitting data for the calculation of K_a for chromone and coumarin binding in the cavity of **2**·$12NO_3$. Cage peaks H_C and H_E for the coumarin titration were too broad under ambient conditions to resolve the peak shift and fit reliably. Discrepancies in y0 values are due to the wetness of the deuterium oxide solvent.

7. NMR spectra of all compounds

Some spectra in Section 6 have been truncated to enhance the clarity of the spectra. None of the regions removed contained any additional signals, from the products or otherwise, with the exception of Figure S53, where only the residual NMR solvent resonance has been removed.

7a. ^{1}H NMR Spectra

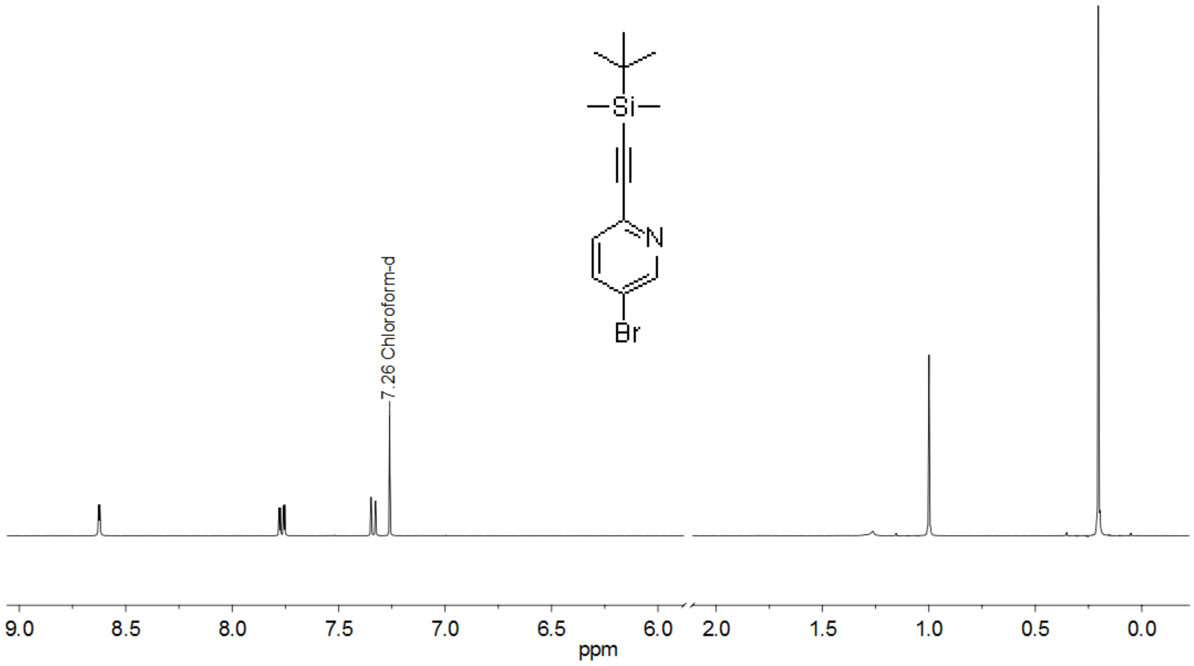

Figure S39: ^{1}H NMR spectrum (400 MHz, $CDCl_3$) of 2-tert-butyldimethylsilylethynyl-5-bromopyridine.

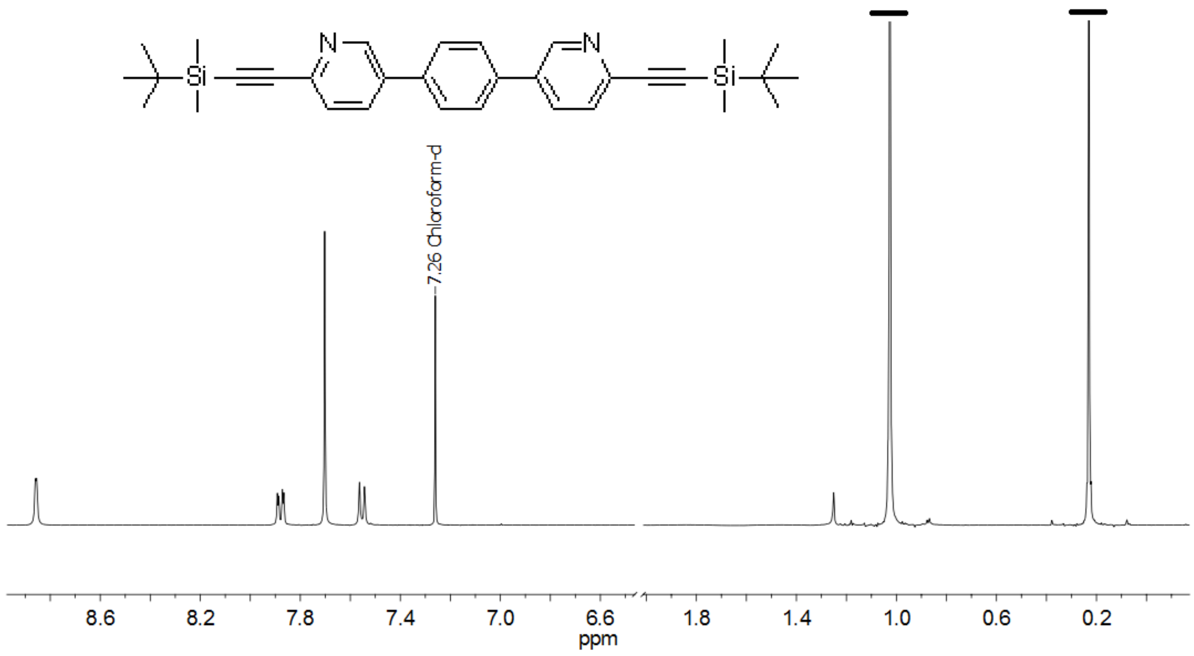

Figure S40: ^{1}H NMR spectrum (400 MHz, $CDCl_3$) of 1,4-bis(6-((tert-butyldimethylsilyl)ethynyl)pyridin-3-yl)benzene.

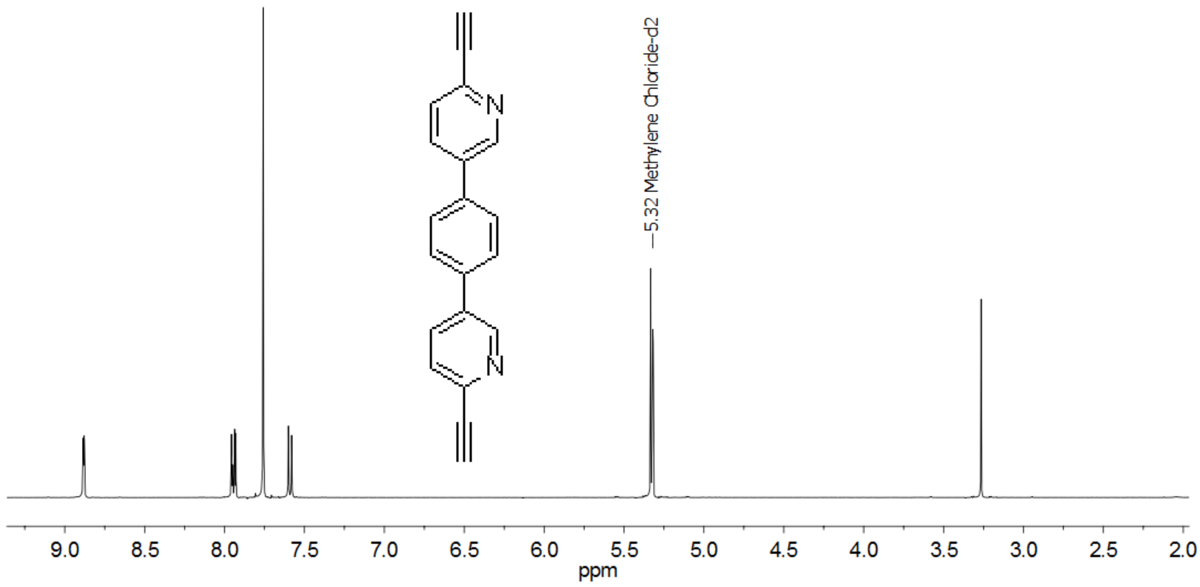

Figure S41: ^{1}H NMR spectrum (400 MHz, CD_2Cl_2) of 1,4-bis(6-ethynylpyridin-3-yl)benzene.

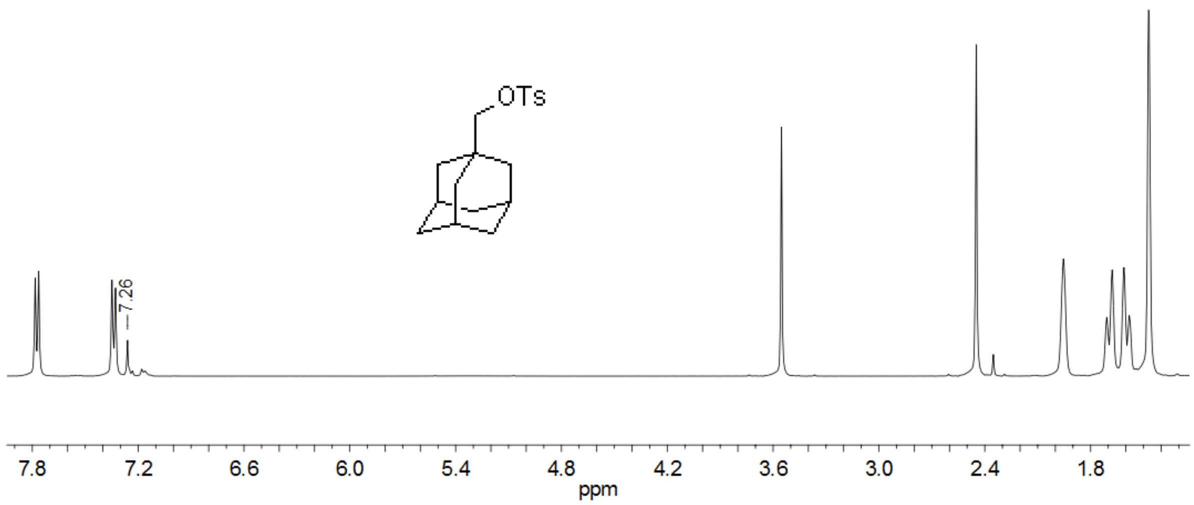

Figure S42: ^{1}H NMR spectrum (400 MHz, $CDCl_3$) of 1-adamantanemethyltosylate.

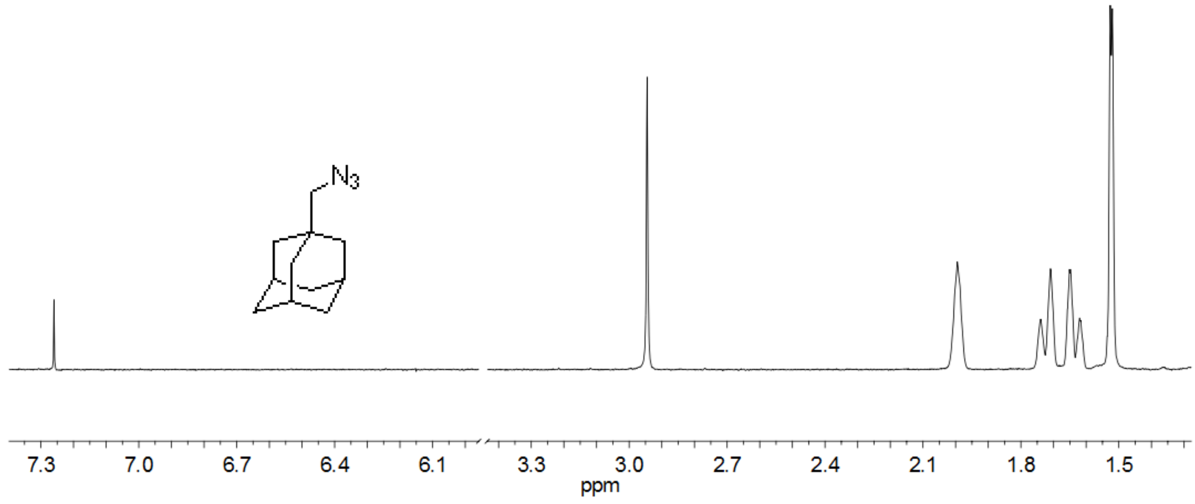

Figure S43: ^{1}H NMR spectrum (400 MHz, $CDCl_3$) of 1-adamantanemethylazide.

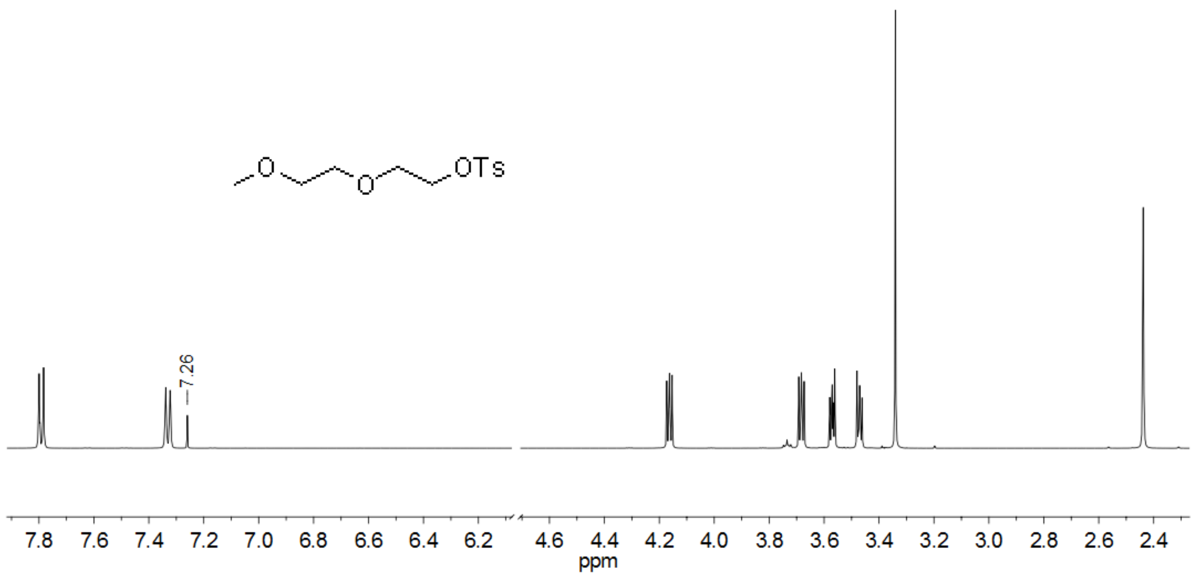

Figure S44: ^{1}H NMR spectrum (500 MHz, $CDCl_3$) of 2-(2-methoxyethoxy)ethyl 4-methylbenzenesulfonate.

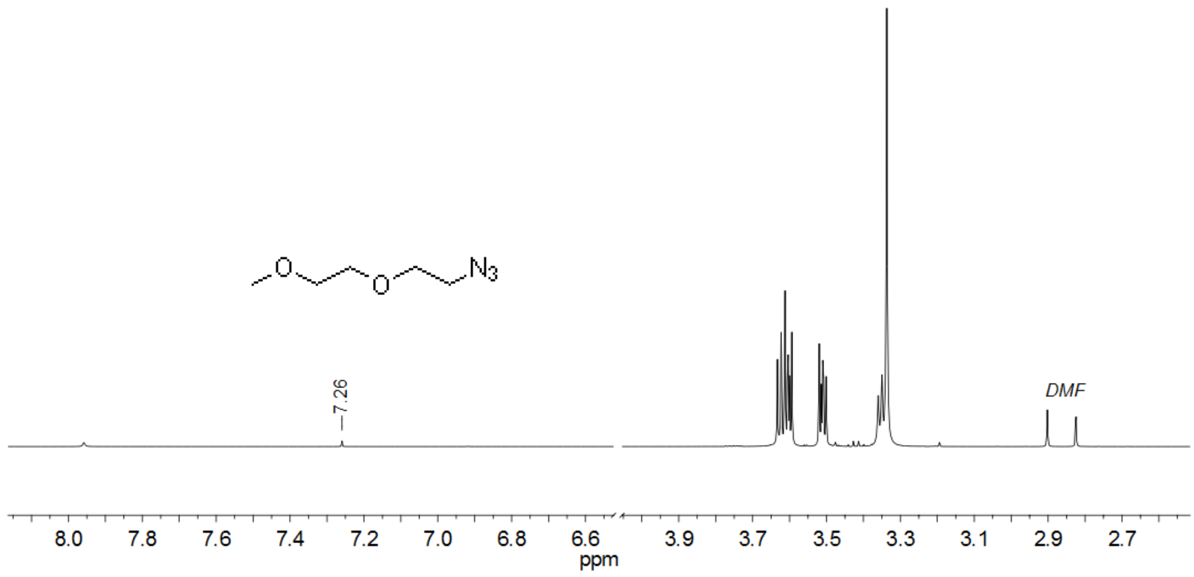

Figure S45: ^{1}H NMR spectrum (500 MHz, $CDCl_3$) of 1-azido-2-(2-methoxyethoxy)ethane.

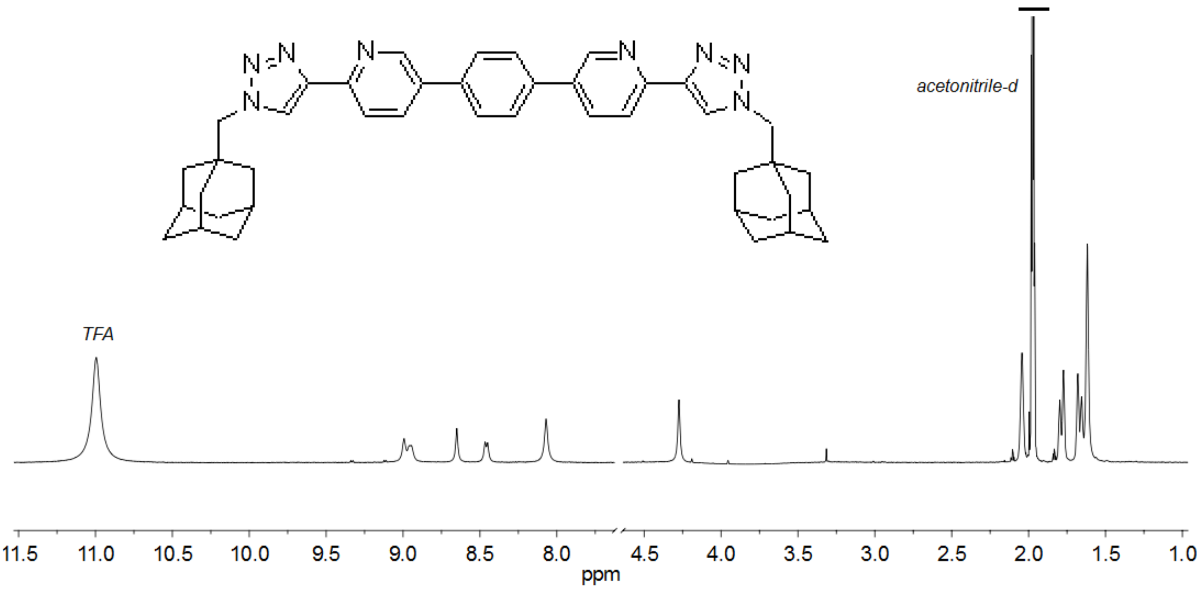

Figure S46: ^{1}H NMR spectrum (400 MHz, CD_3CN-TFA-*d*) of **L**[1].

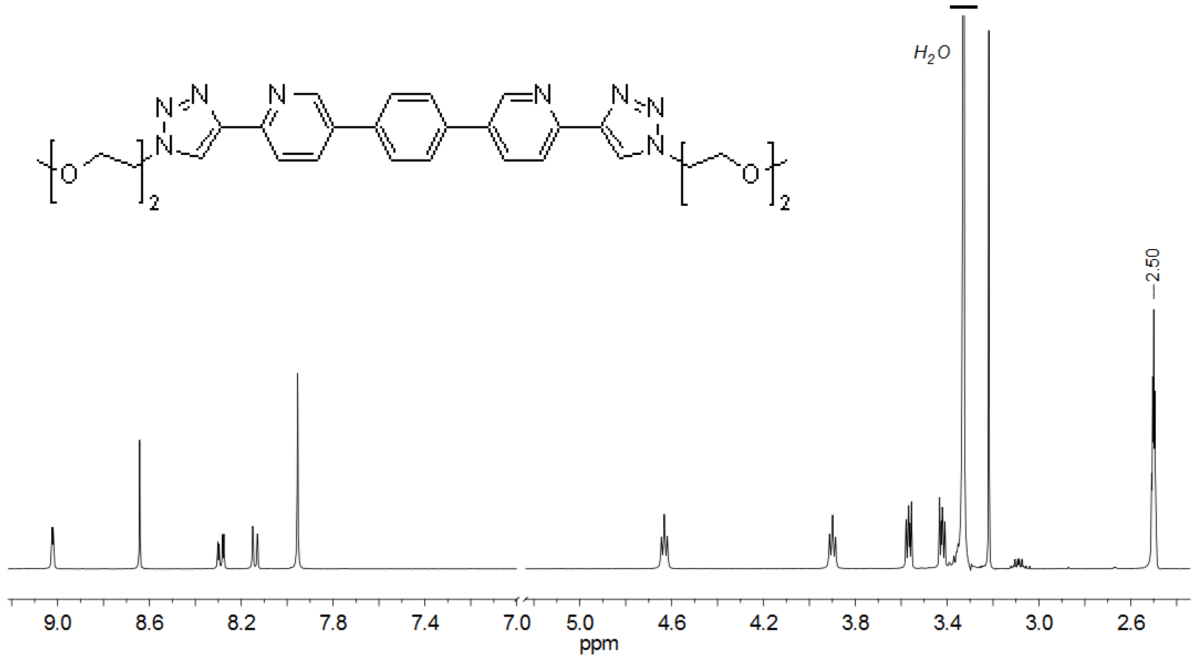

Figure S47: ^{1}H NMR spectrum (400 MHz, DMSO-*d*) of **L**[2]**.**

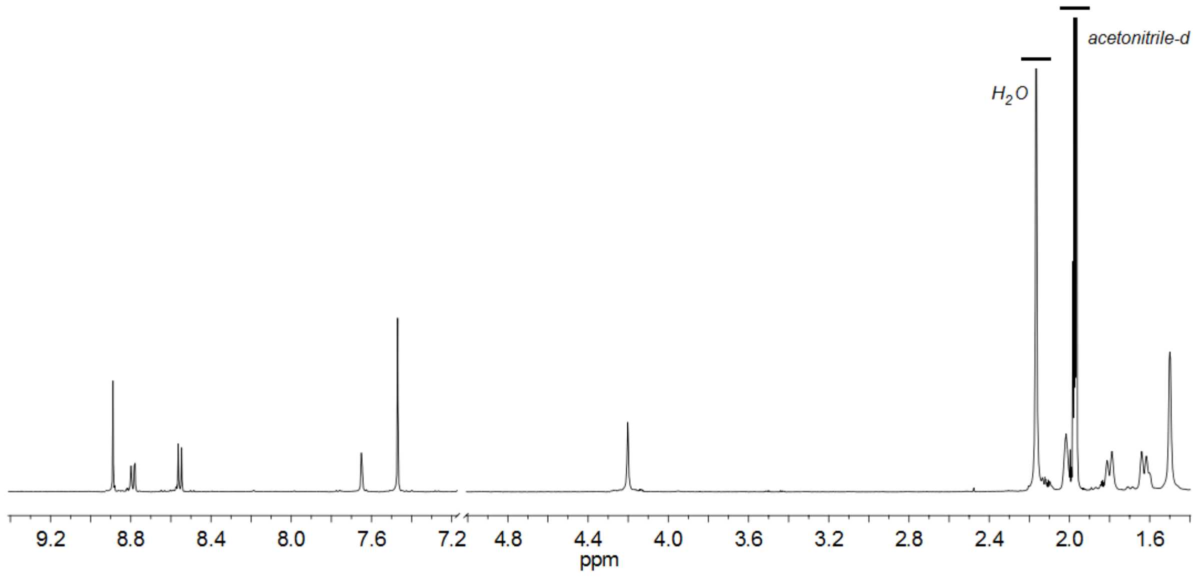

Figure S48: ^{1}H NMR spectrum (500 MHz, CD_3CN) of **1**·12PF_6.

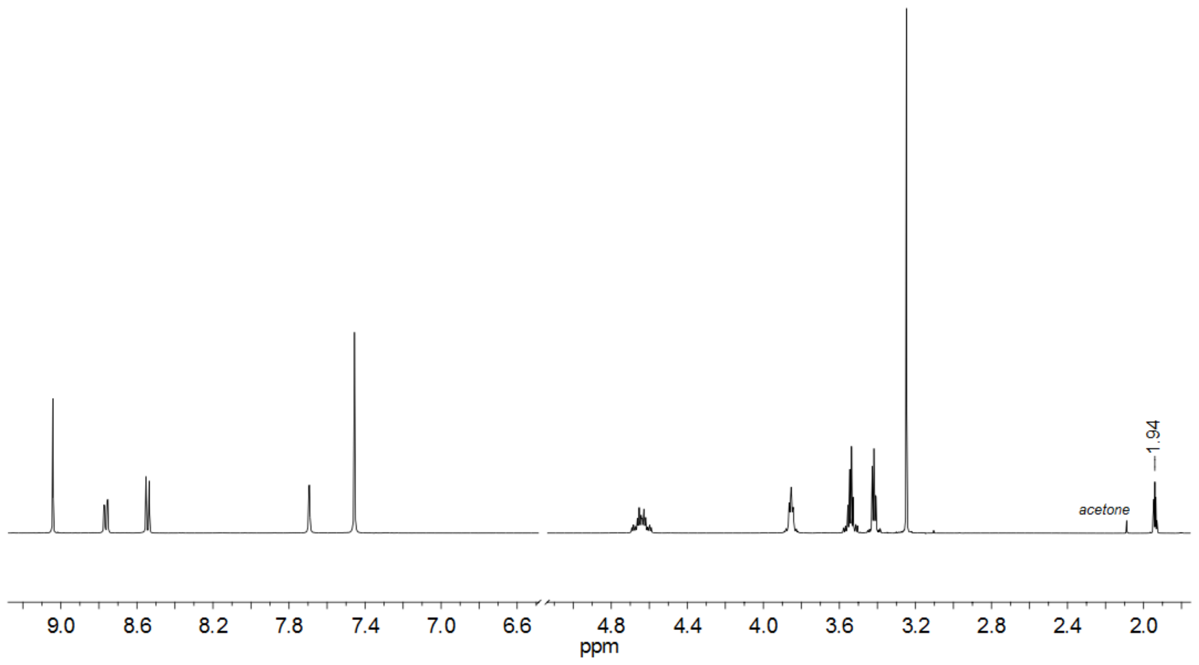

Figure S49: ^{1}H NMR spectrum (500 MHz, CD_3CN) of **2**·12PF_6.

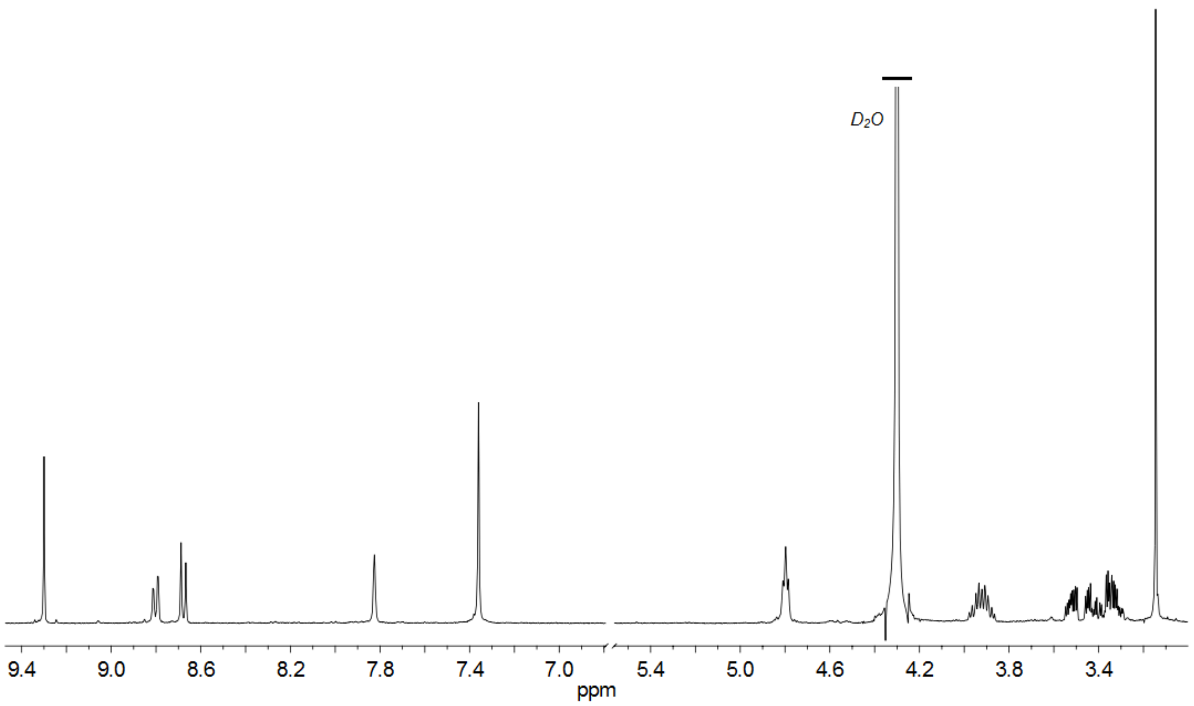

Figure S50: ^{1}H NMR spectrum (400 MHz, D_2O, 343 K) of **2**·12NO_3.

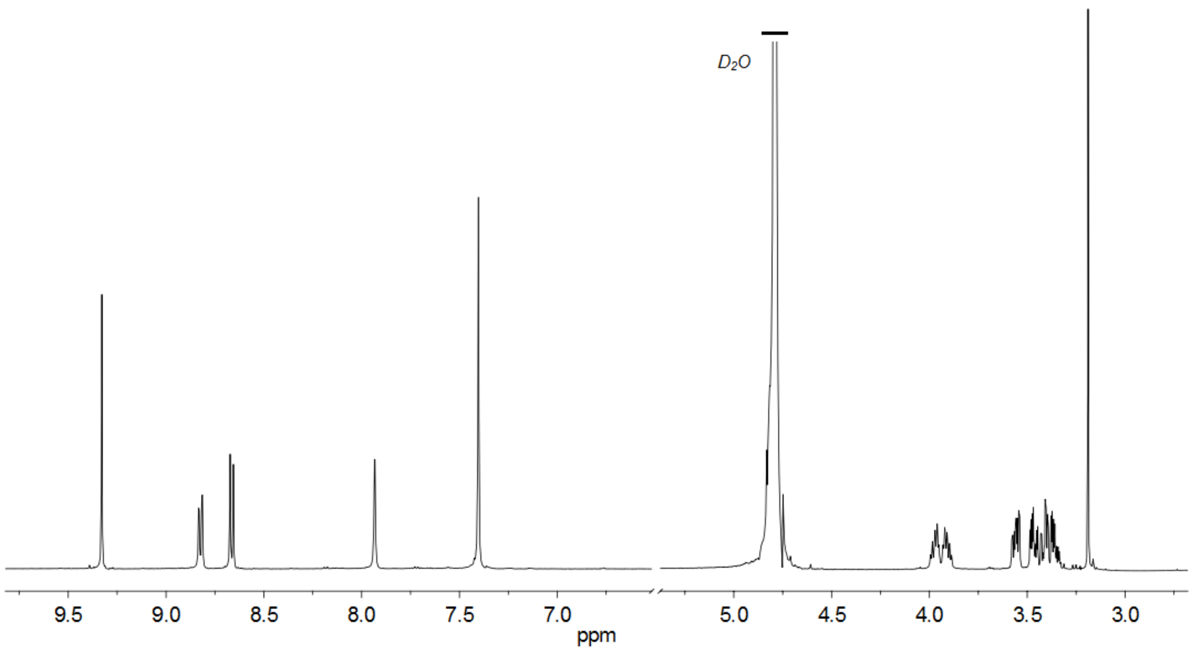

Figure S51: ^{1}H NMR spectrum (500 MHz, D_2O, 298 K) of **2**.12NO_3.

7b. ^{13}C NMR Spectra

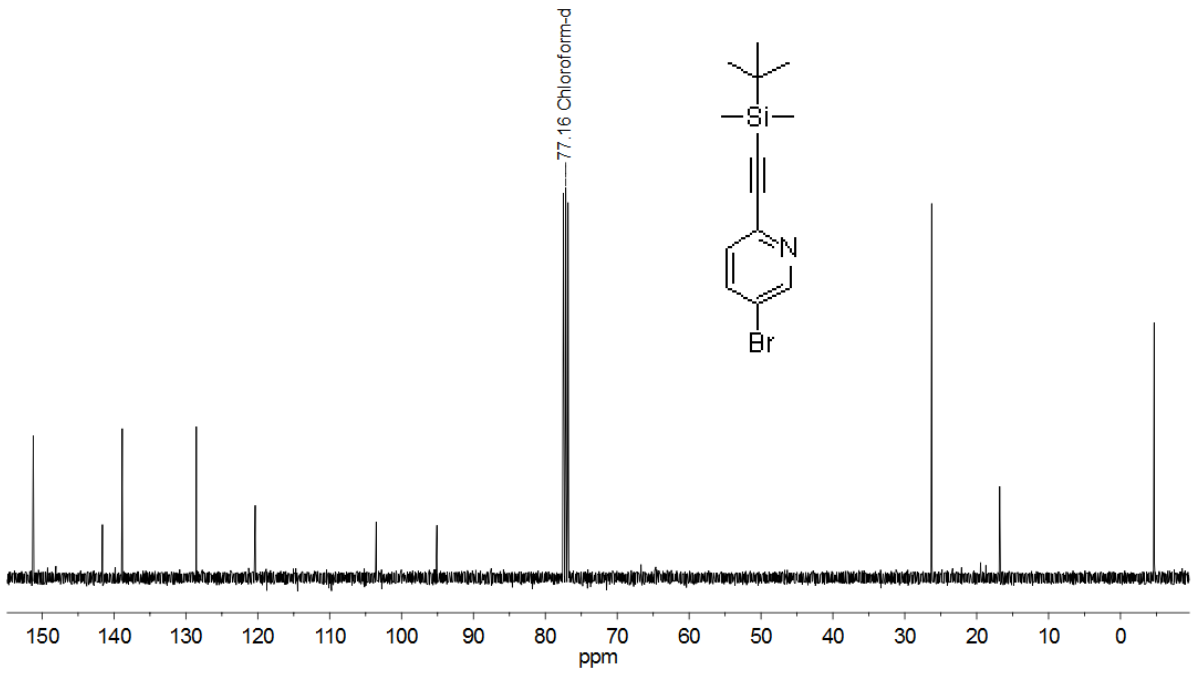

Figure S52: ^{13}C NMR spectrum (126 MHz, $CDCl_3$) of 2-tert-butyldimethylsilylethynyl-5-bromopyridine.

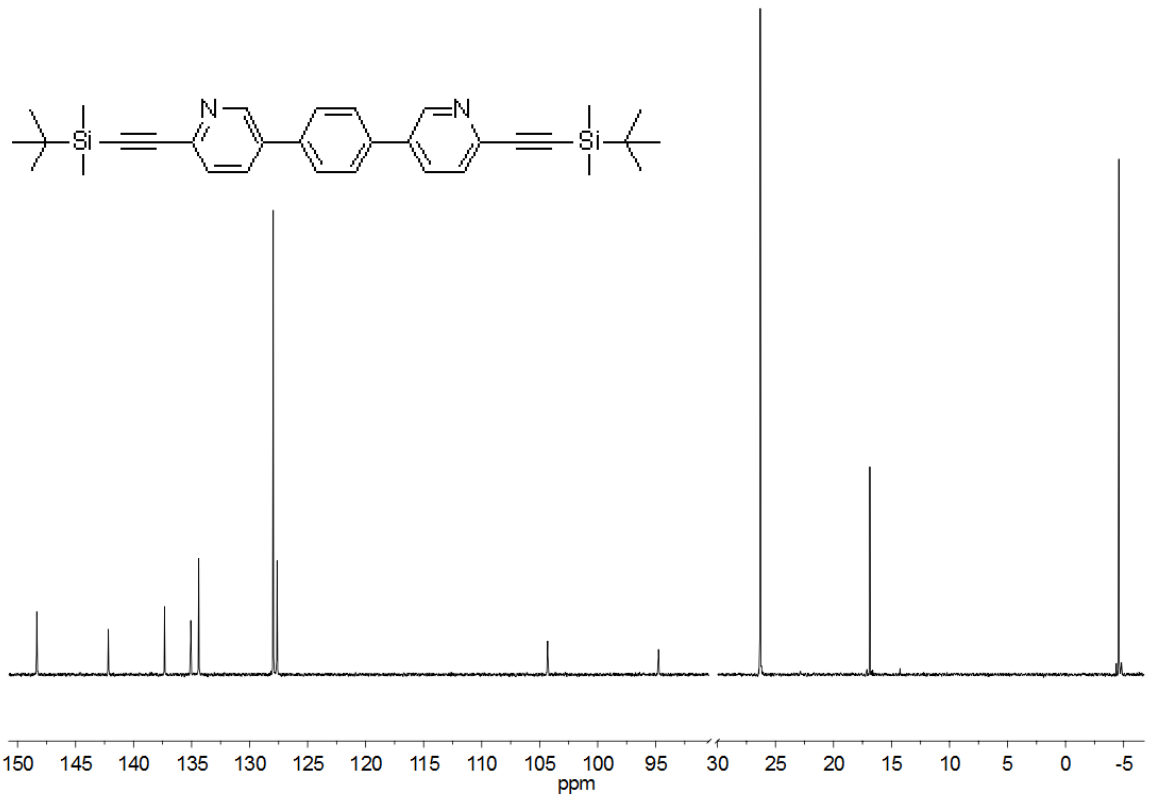

Figure S53: ^{13}C NMR spectrum (126 MHz, $CDCl_3$) of 1,4-bis(6-((tert-butyldimethylsilyl)ethynyl)pyridin-3-yl)benzene.

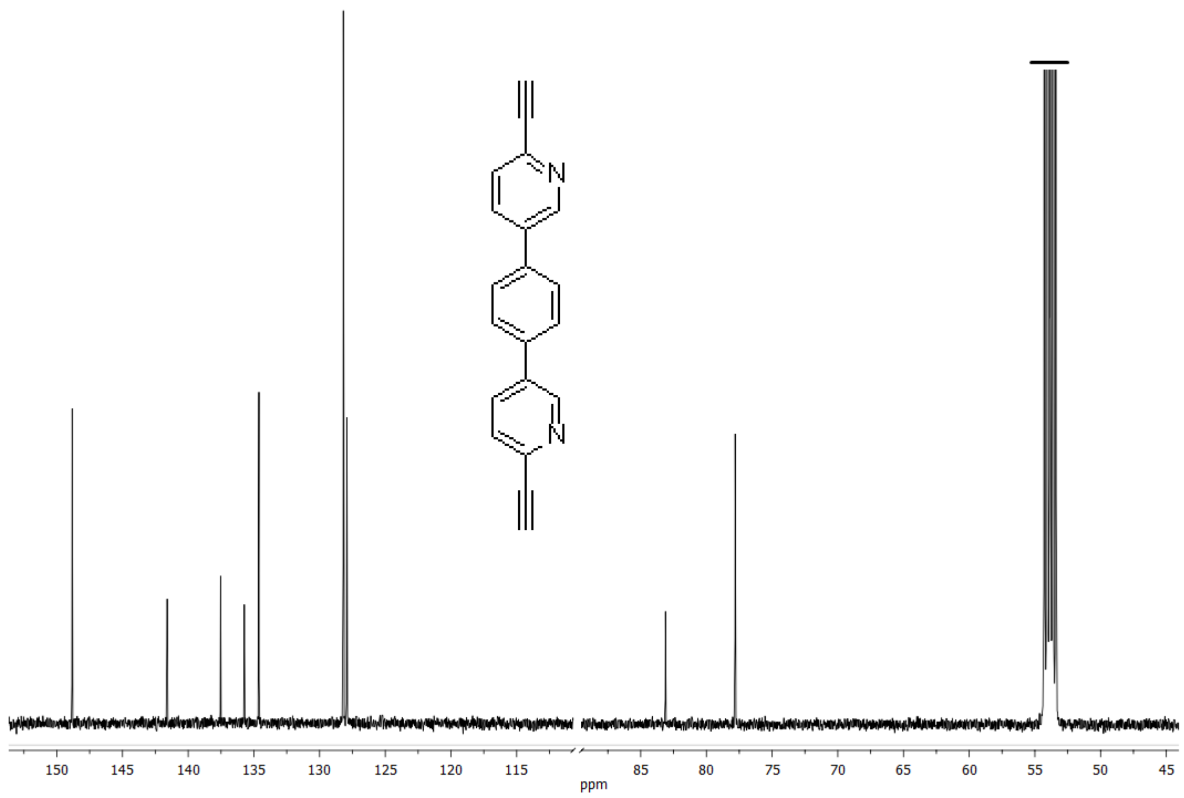

Figure S54: ^{13}C NMR spectrum (126 MHz, CD_2Cl_2) of 1,4-bis(6-ethynylpyridin-3-yl)benzene.

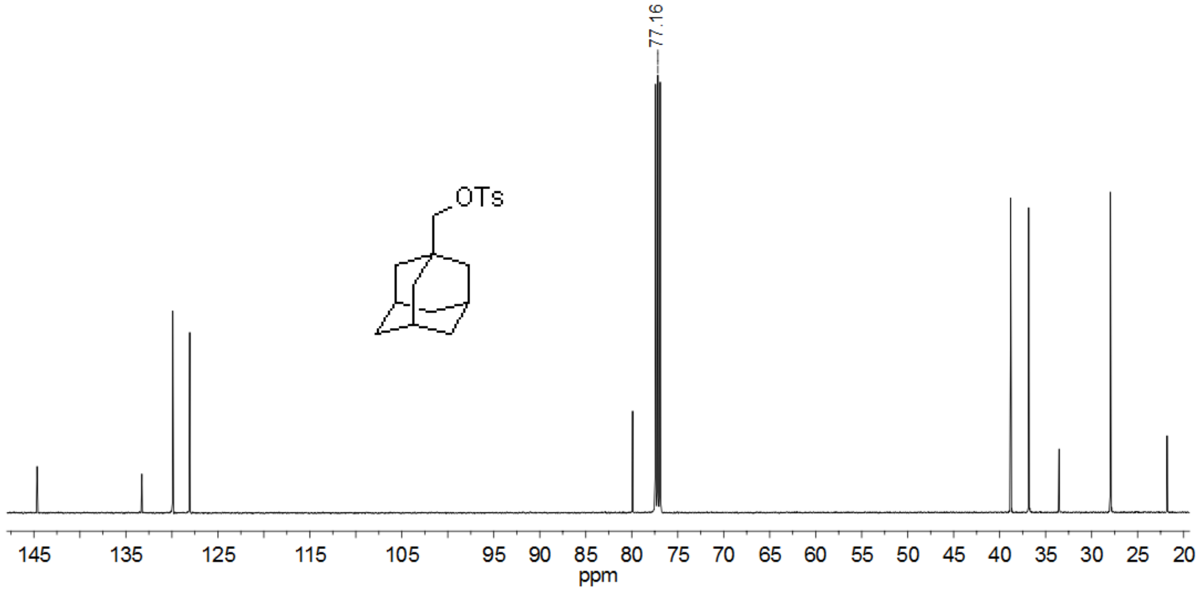

Figure S55: ^{13}C NMR spectrum (126 MHz, $CDCl_3$) of 1-adamantanemethyltosylate.

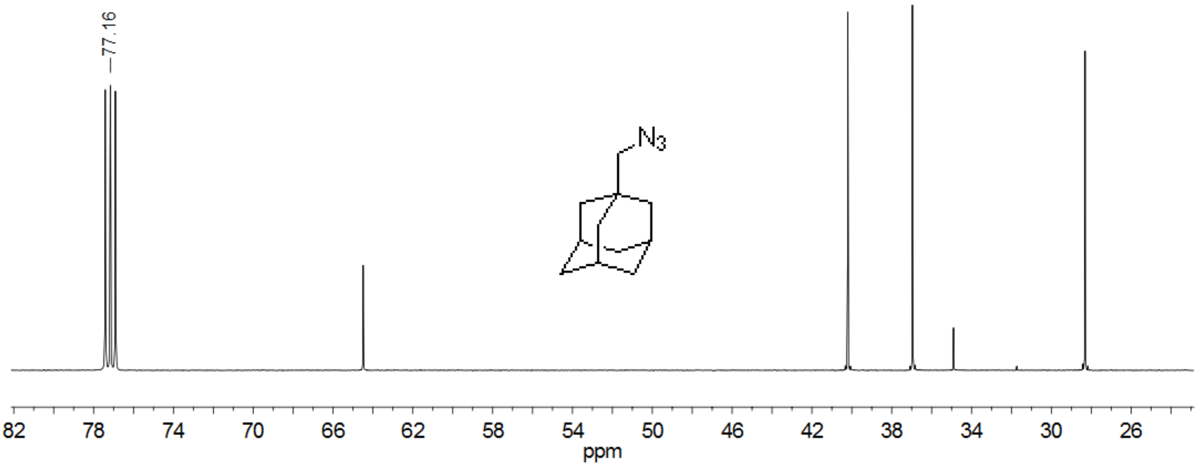

Figure S56: ^{13}C NMR spectrum (126 MHz, $CDCl_3$) of 1-adamantanemethylazide.

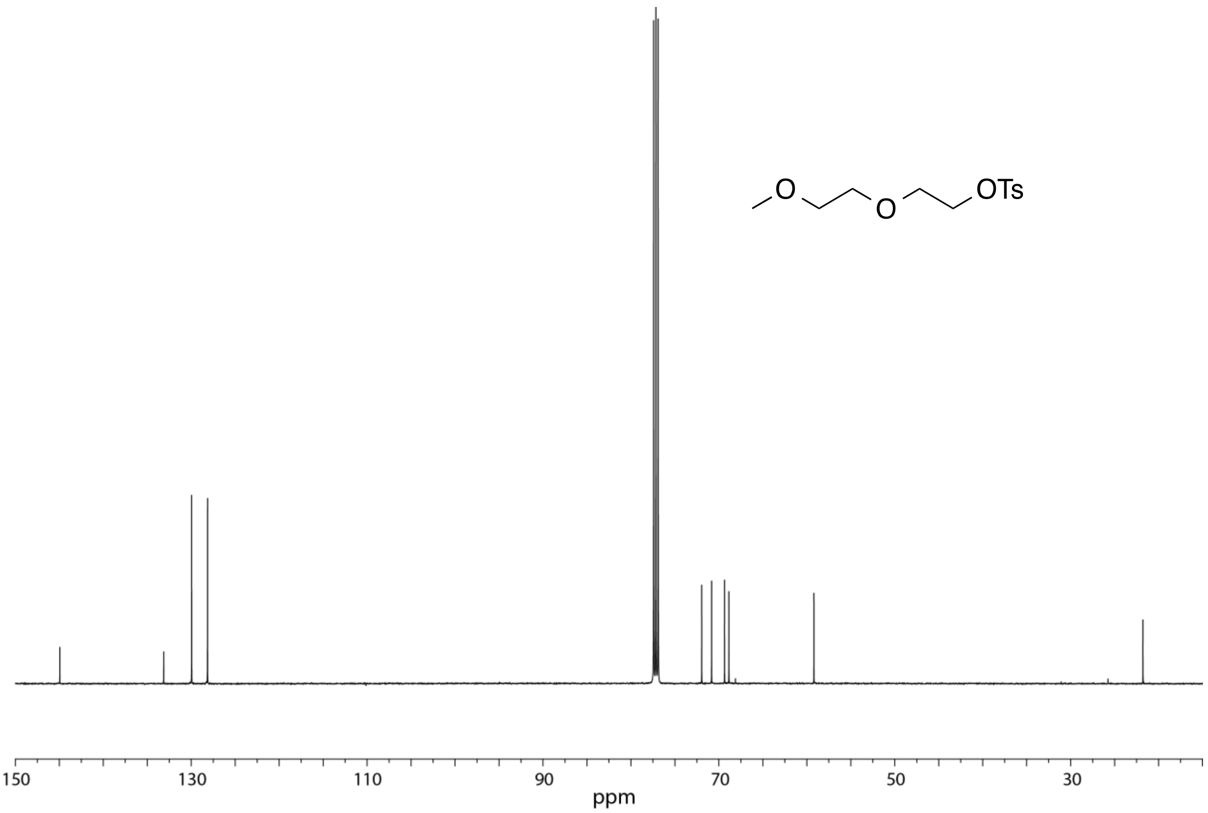

Figure S57: ^{13}C NMR spectrum (126 MHz, $CDCl_3$) of 2-(2-methoxyethoxy)ethyl 4-methylbenzenesulfonate.

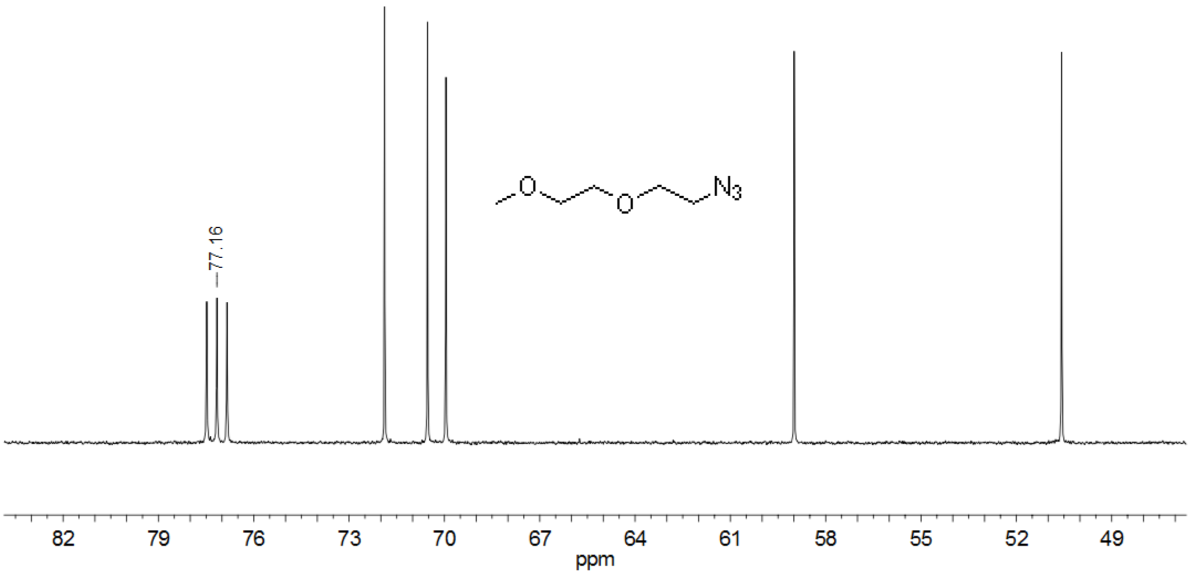

Figure S58: ^{13}C NMR spectrum (126 MHz, $CDCl_3$) of 1-azido-2-(2-methoxyethoxy)ethane.

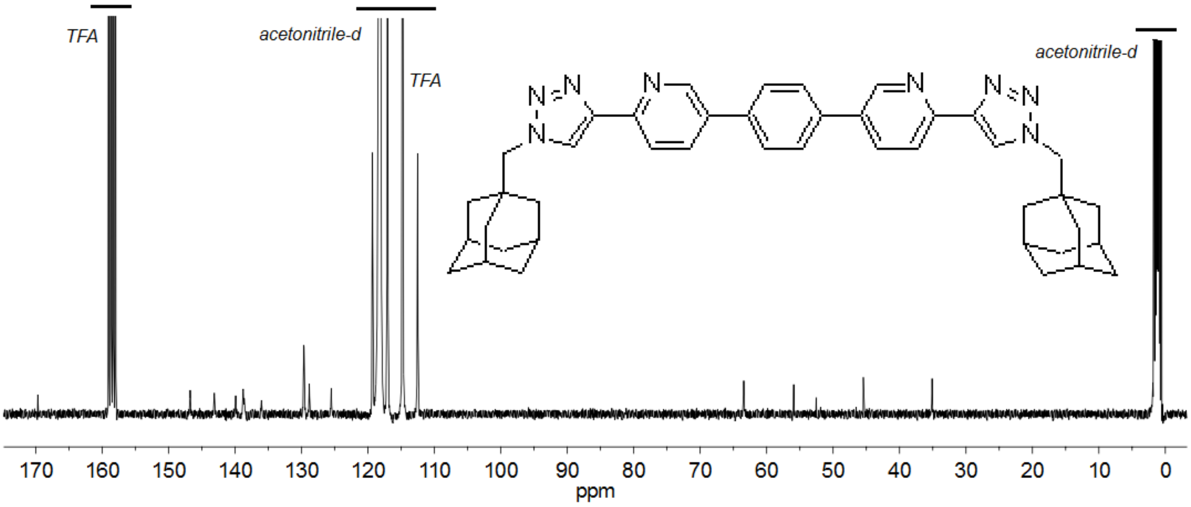

Figure S59: ^{13}C NMR spectrum (126 MHz, CD_3CN-TFA-*d*) of **L**[1].

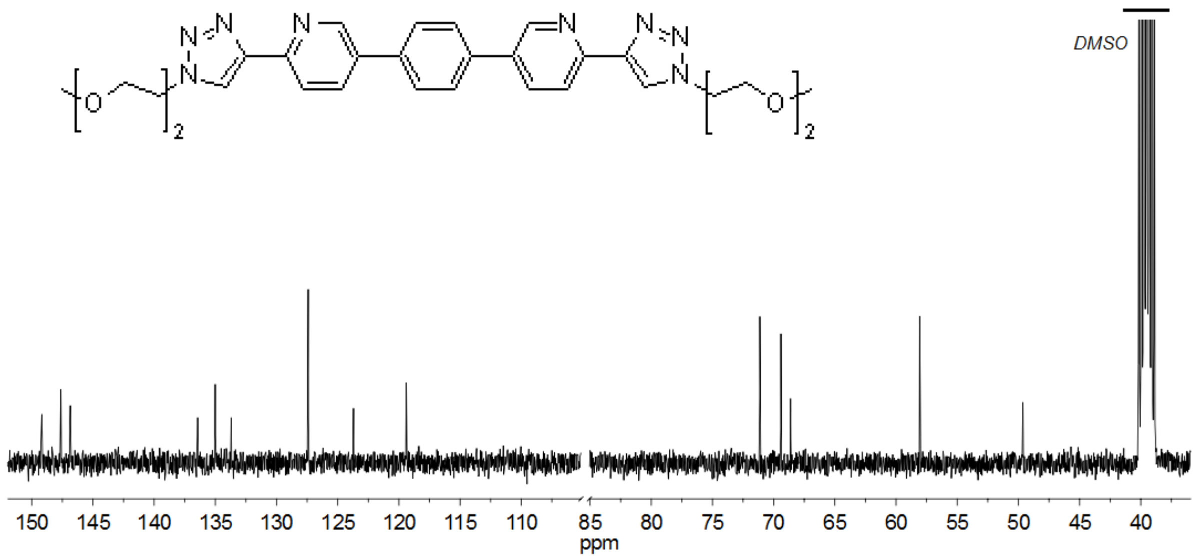

Figure S60: ^{13}C NMR spectrum (126 MHz, DMSO-*d*) of $\mathbf{L}^2$.

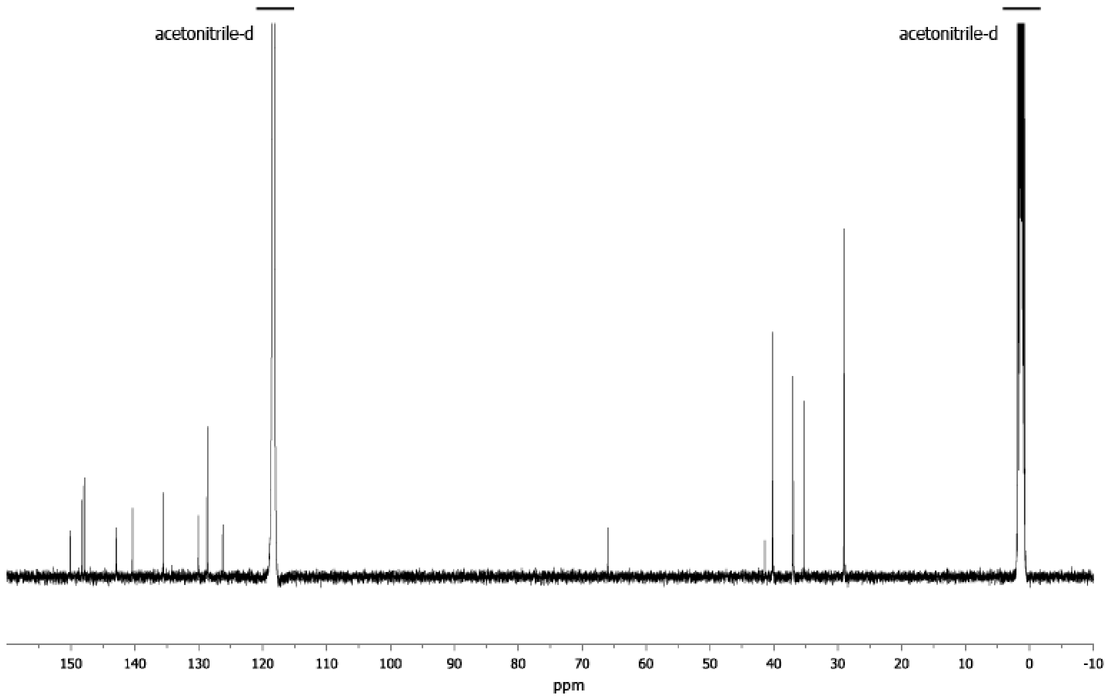

Figure S61: ^{13}C NMR spectrum (126 MHz, CD_3CN) of **1**·$12PF_6$.

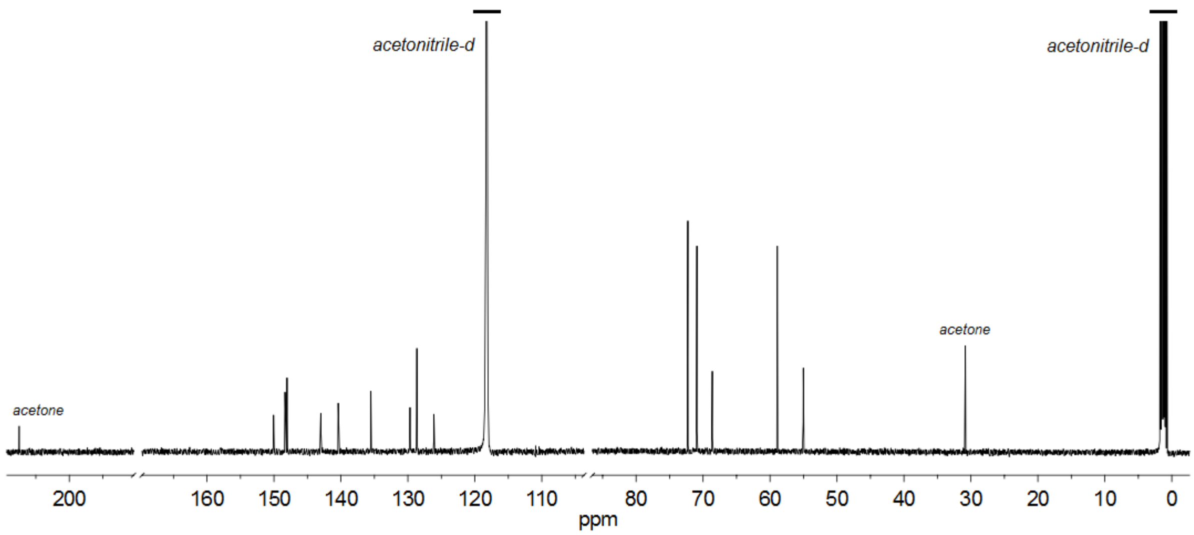

Figure S62: ^{13}C NMR spectrum (126 MHz, CD_3CN) of **2**·12PF_6.

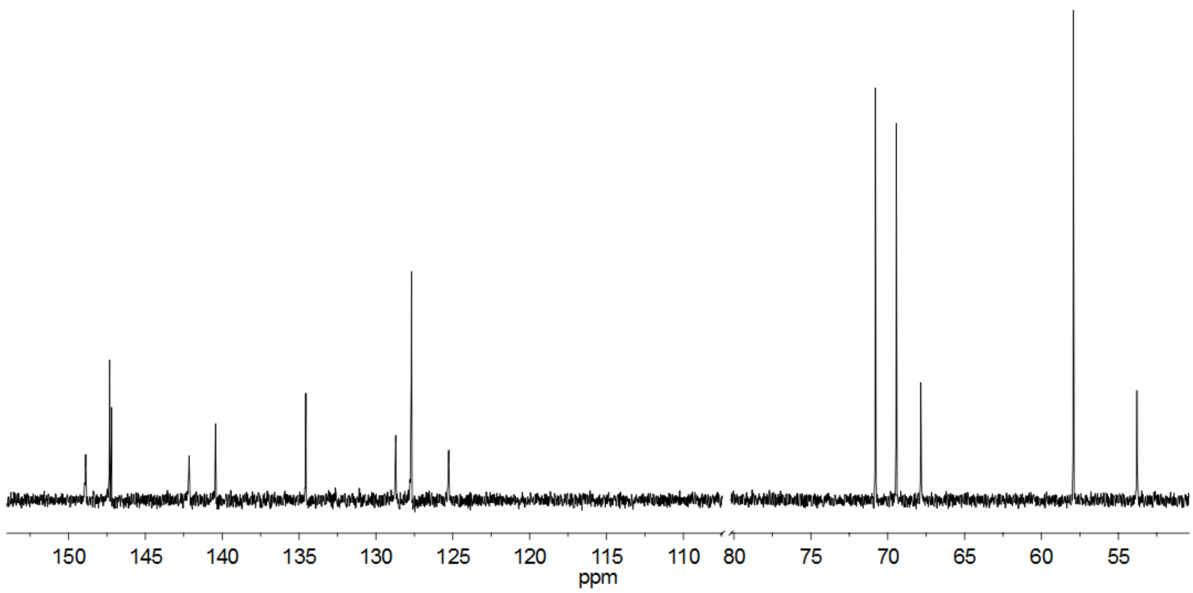

Figure S63: ^{13}C NMR spectrum (126 MHz, D_2O, 298 K) of **2**·12NO_3.

7c. ^{1}H DOSY Spectra of Complexes

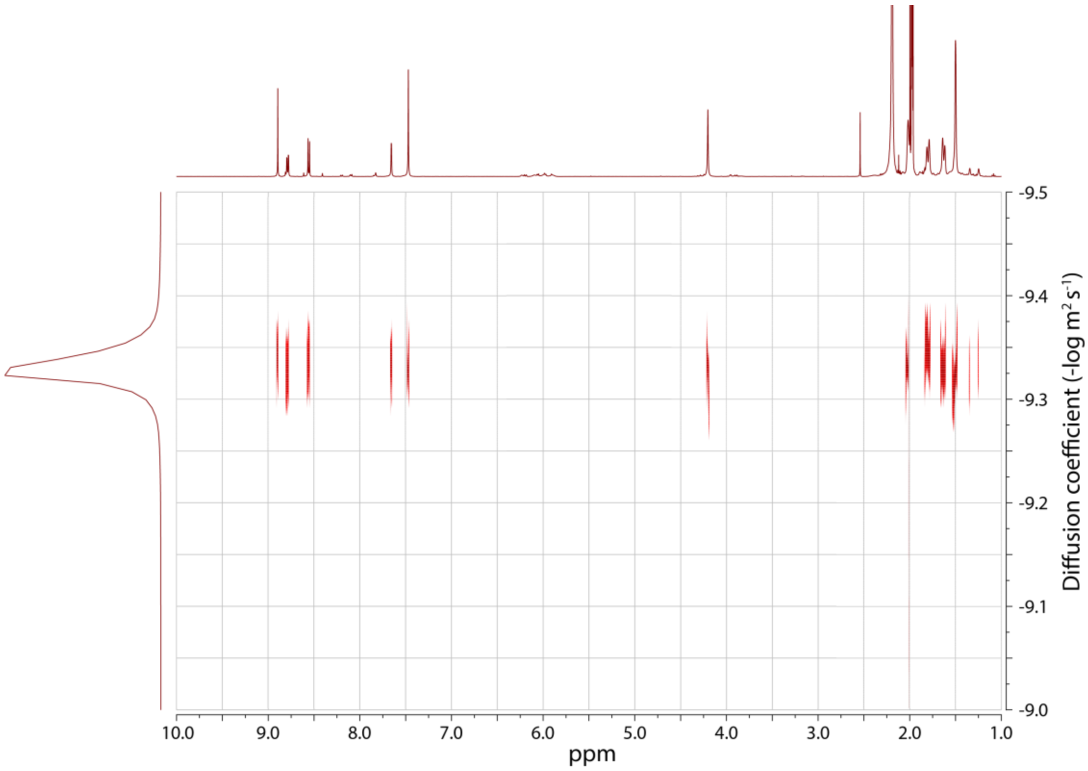

Figure S64: ^{1}H DOSY NMR spectrum (500 MHz, CD_3CN) of **1**·$12PF_6$.

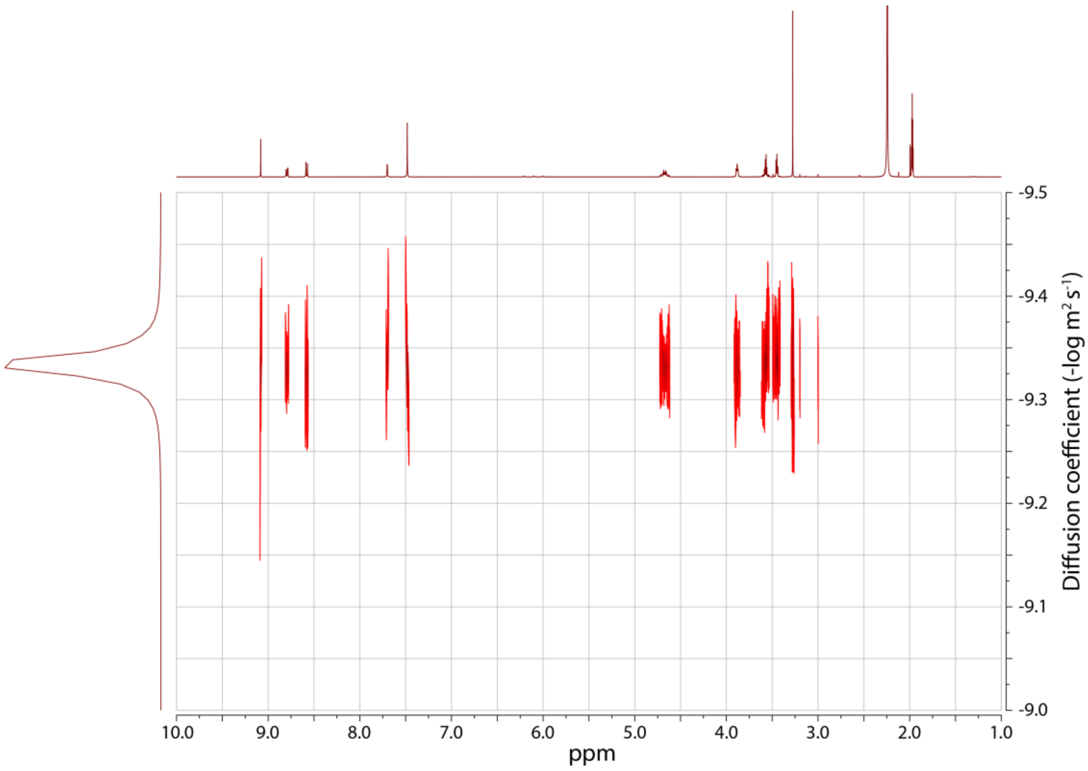

Figure S65: ^{1}H DOSY NMR spectrum (500 MHz, CD_3CN) of **2**·$12PF_6$.

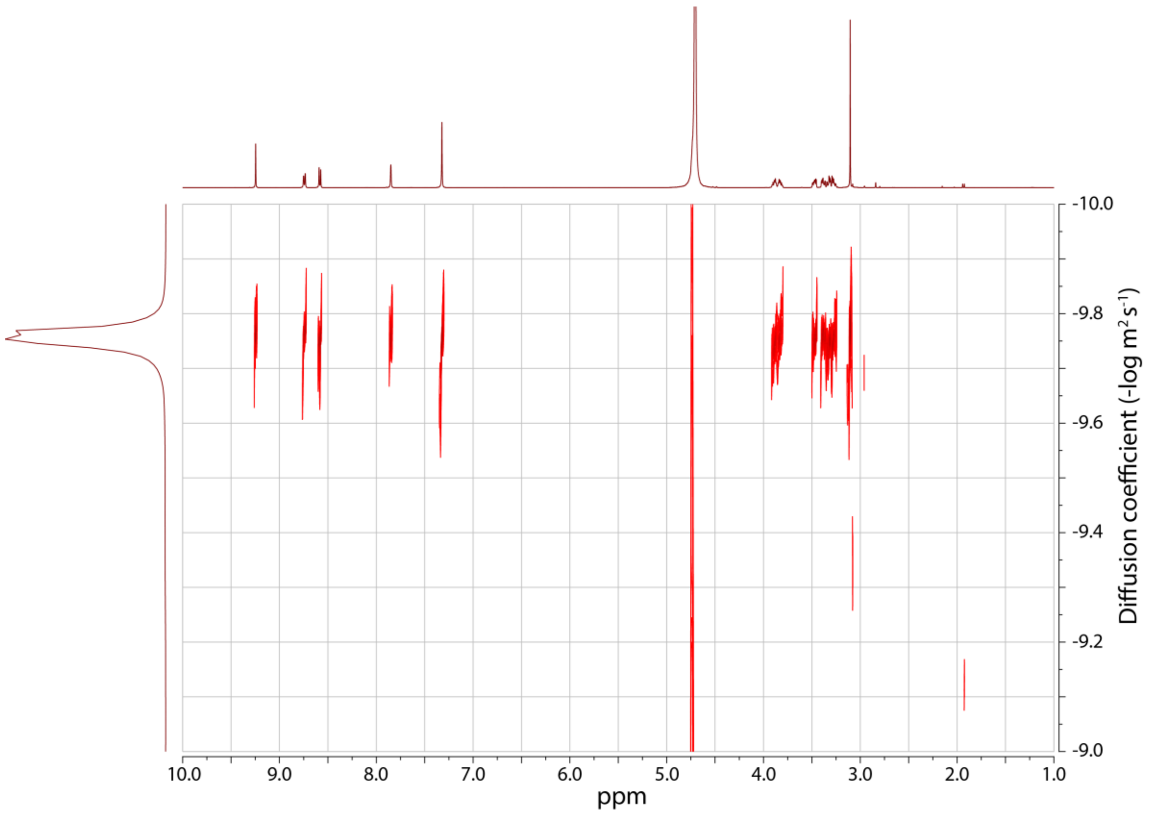

Figure S66: ^{1}H DOSY NMR spectrum (500 MHz, D_2O) of **2**·12NO_3.

8. Mass Spectrometry of Complexes

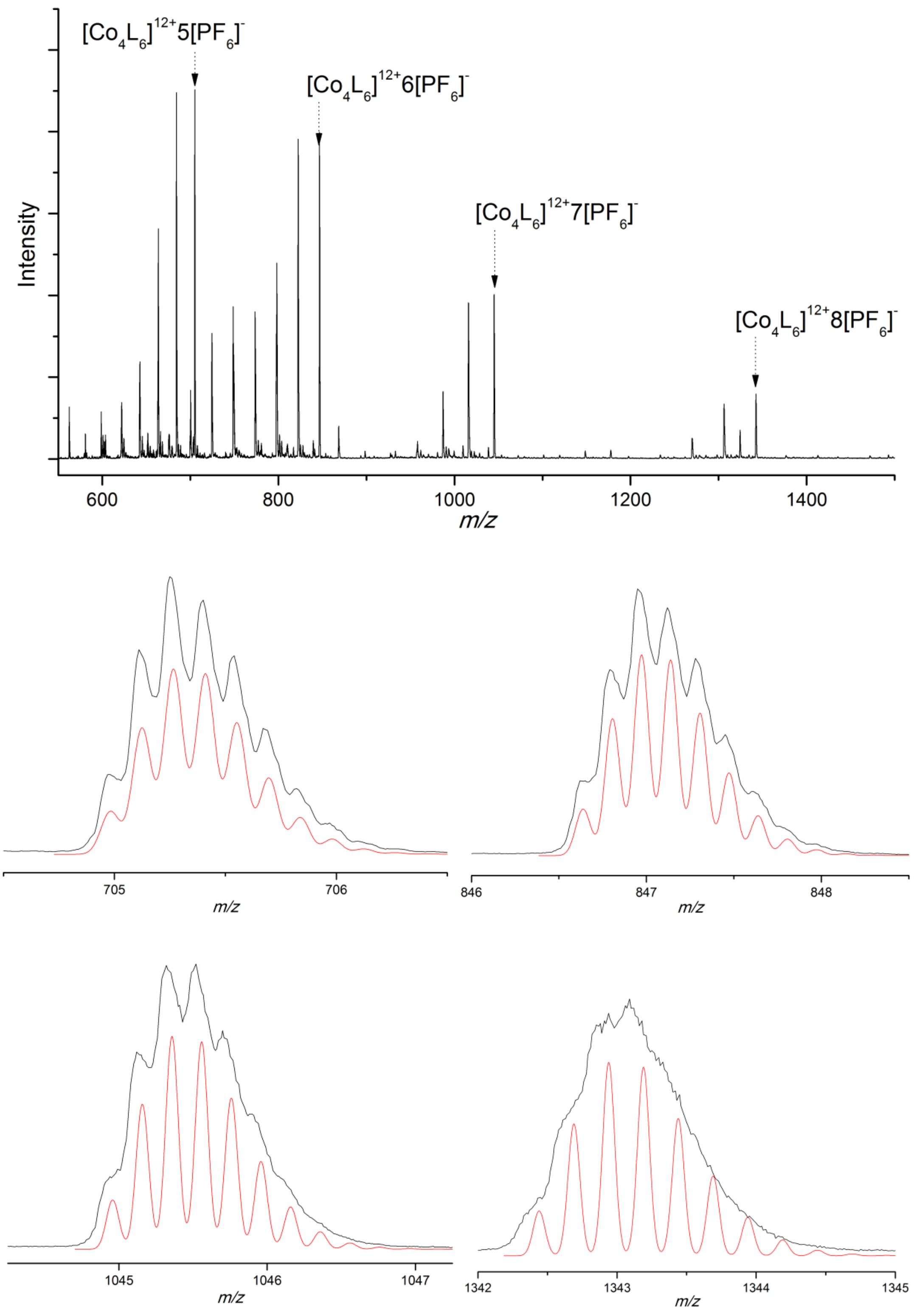

Figure S67: Mass Spectrum of **1**·$12PF_6$. Full spectrum (top) is shown with expansions of the labelled peaks (bottom) to demonstrate isotopic distribution. Recorded data (black) is shown against modelled data for the tetrahedron (red).

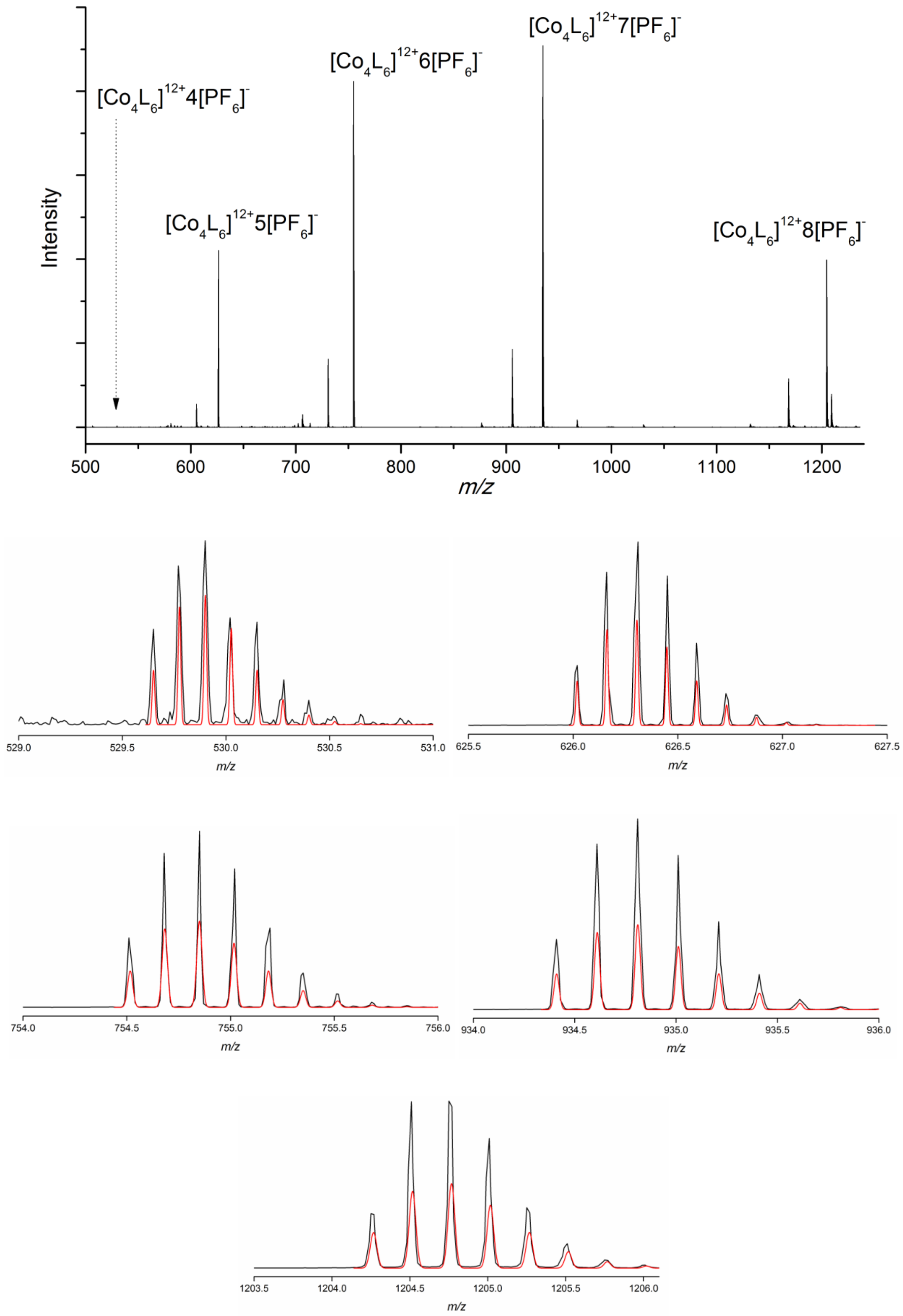

Figure S68: Mass Spectrum of **2**·12PF_6. Full spectrum (top) is shown with expansions of the labelled peaks (bottom) to demonstrate isotopic distribution. Recorded data (black) is shown against modelled data for the tetrahedron (red).

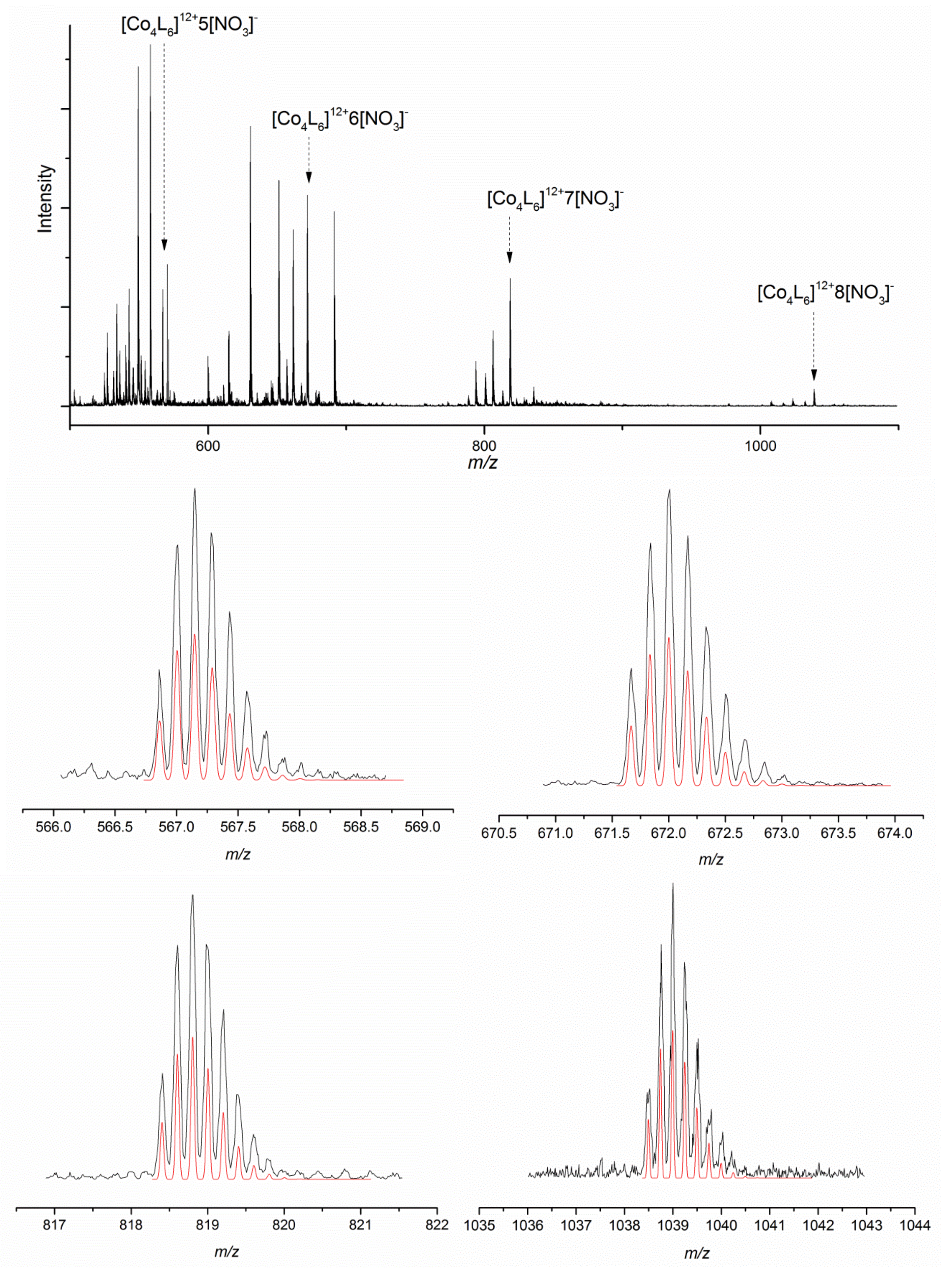

Figure S69: Mass Spectrum of **2**·$12NO_3$. Full spectrum (top) is shown with expansions of the labelled peaks (bottom) to demonstrate isotopic distribution. Recorded data (black) is shown against modelled data for the tetrahedron - corrected for calibration (red).

9. X-ray Crystallography

Capillary-mounted single-crystal X-ray diffraction data of **1**·$12PF_6$ were collected on beamline I19 at the DIAMOND Light Source (λ = 0.6889 Å). Data were collected on a Rigaku 720+ detector in ω-scans with an exposure time and scan width of one second and 1° respectively with a detector distance of 60 mm.

Suitable crystals were grown *via* slow diffusion of diisopropyl ether into an acetonitrile solution of complex **1**·$12PF_6$ producing well-formed dark orange crystals. The crystals were observed to desolvate immediately when removed from the mother liquor; the rate of desolvation was such that the crystal integrity was quickly destroyed in a shorter timeframe than it takes to select and mount a suitable crystal on the diffractometer. To aid with data collection a small sample was sent to Dr Mark Warren at Diamond Light Source. The crystal was mounted inside a capillary along with some mother liquor to prevent desolvation. As a consequence the orientation of the crystal was not fixed with respect to the goniometer during data collection (i.e. the crystal unavoidably moved about) and so the complete data set consists of, effectively, multiple overlapping diffraction patterns with no fixed orientation relationship between them. This was partly corrected for by integrating using the "follow sudden discontinuous sample orientation changes" option in the *CrysAlisPro* software.[7] The software does a good job of modelling the crystal motion during data collection, but is not perfect. Attempts at deconvoluting the overlapping diffraction patterns using routines used for twinned crystals were not successful.

Despite the experimental challenges the structure solved with relative ease using direct methods, and the asymmetric unit is shown in Figure S64.[8] All non-H atoms were easily observed in a difference Fourier map; H atoms were placed geometrically. The complete cluster is shown in Figure S65.

A couple of minor issues with regard refinement were encountered, these were:

1. Two PF_6^- ions per asymmetric unit (x3 = 6 per complete cluster) were easily identified and refined without any sort of geometric or displacement ellipsoid restraints. However, this is not enough to balance the expected charge since each Co site has been assigned oxidation state +3. Whilst the rest of the characterisation data are consistent with Co(III) centres, likewise the Co-N bond lengths, which range from 1.881(8)-2.037(8) Å, also points to trivalent metal ions (c.f. the numerous cage structures from Michael Ward's group, which contain distorted octahedral Co(II) pyridyl-pyrazole coordination motifs,[9] possess Co-N

bonds which are typically in the range 2.1-2.2 Å). The largest residual peak in a difference Fourier map is 2.42 electrons high; it is almost impossible to identify the remaining counterions (which could be either hexafluorophosphate, nitrate or perchlorate based on the synthetic procedure) from the residual difference map.

2. Charge balance issues aside, the structure contains a lot of residual electron density which is smeared over a very large region of space and simply cannot be modelled using discrete atoms to form chemically sensible, reasonable fragments. The solvent masking procedure of the program OLEX2[10] was used to identify electron density consisting of 6518 electrons in a volume of 27355 $Å^3$ in the unit cell. Standard crystallographic practice is to assign this missing density to a proportion of missing solvent or (less commonly) counterions and include this solvent in the chemical formula and all derived values (molecular mass, crystal density etc.). In this instance to do so would be complete guesswork and has not been attempted.

CCDC 1014311 contains the supplementary crystallographic data for this paper. This data can be obtained free of charge from The Cambridge Crystallographic Data Centre via www.ccdc.cam.ac.uk/data_request/cif.

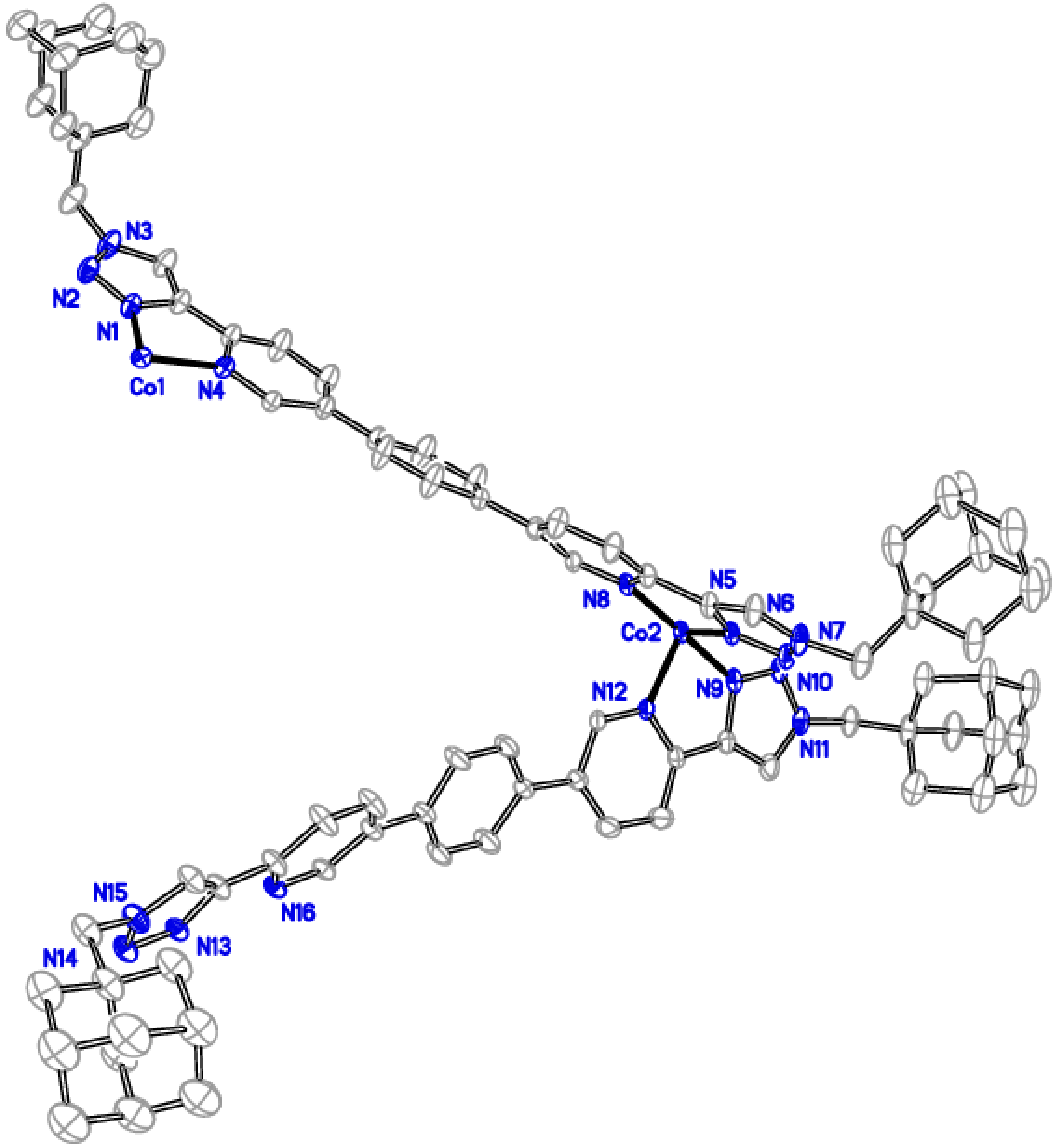

Figure S70: The asymmetric portion of structure **1**·12PF_6, with displacement ellipsoids at the 10% probability level. H atoms and counteranions are omitted.

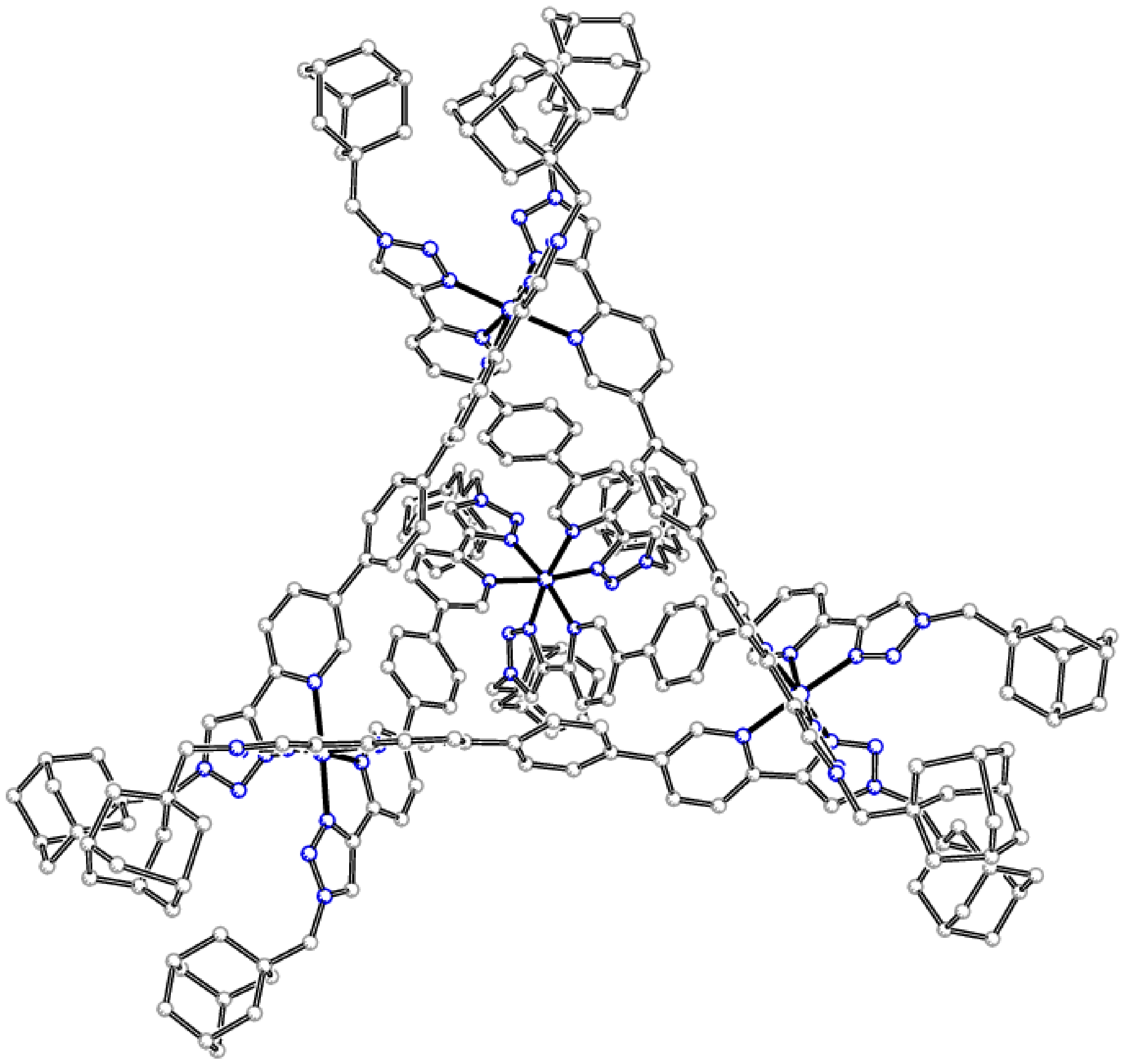

Figure S71: The complete cluster of **1**·12PF_6. H atoms and counteranions are omitted.

Table S72: Crystal data and structure refinement for **1**·12PF_6.

Identification code	pl4001	
Empirical formula	C252 H276 Co4 F36 N48 P6	
Formula weight	5082.73	
Temperature	298 K	
Wavelength	0.6889 Å	
Crystal system	Trigonal	
Space group	R -3	
Unit cell dimensions	a = 30.5162(6) Å	α = 90°.
	b = 30.5162(6) Å	β = 90°.
	c = 71.022(2) Å	γ = 120°.
Volume	57278(3) Å^3	
Z	6	
Density (calculated)	0.884 Mg/m^3	
Absorption coefficient	0.236 mm^{-1}	
F(000)	15876	
Crystal size	0.07 x 0.07 x 0.03 mm^3	
Theta range for data collection	1.519 to 16.681°.	
Index ranges	-25<=h<=25, -25<=k<=25, -59<=l<=59	
Reflections collected	54444	
Independent reflections	7730 [R(int) = 0.1625]	
Completeness to theta = 24.415°	33.5 %	
Absorption correction	Semi-empirical from equivalents	
Max. and min. transmission	1.00000 and 0.33798	
Refinement method	Full-matrix least-squares on F^2	

Data / restraints / parameters	7730 / 1113 / 1039
Goodness-of-fit on F^2	1.553
Final R indices [I>2sigma(I)]	R1 = 0.1218, wR2 = 0.3268
R indices (all data)	R1 = 0.1352, wR2 = 0.3455
Extinction coefficient	n/a
Largest diff. peak and hole	0.431 and -0.762 e.Å^{-3}

Table S73: Atomic coordinates (x10^4) and equivalent isotropic displacement parameters ($Å^2$x 10^3) for **1**·12PF_6. U(eq) is defined as one third of the trace of the orthogonalized U^{ij} tensor.

	x	y	z	U(eq)
Co(1)	6667	3333	5849(1)	84(1)
Co(2)	6267(1)	5627(1)	7384(1)	72(1)
N(1)	7274(3)	3754(3)	5705(1)	96(2)
N(2)	7458(4)	3694(4)	5547(1)	109(3)
N(3)	7867(4)	4128(4)	5512(1)	124(3)
N(4)	6973(3)	3983(3)	6004(1)	86(2)
N(5)	5617(3)	5555(2)	7415(1)	73(2)
N(6)	5425(3)	5714(3)	7546(1)	91(2)
N(7)	4951(4)	5556(3)	7482(2)	113(3)
N(8)	5954(3)	5251(2)	7149(1)	71(2)
N(9)	6486(3)	5939(3)	7620(1)	82(2)
N(10)	6704(3)	6409(3)	7675(2)	96(2)
N(11)	6752(3)	6400(4)	7861(2)	103(3)
N(12)	6058(3)	5020(3)	7530(1)	74(2)
N(13)	3749(3)	273(3)	7256(1)	79(2)
N(14)	3462(3)	-209(3)	7212(1)	93(2)
N(15)	3066(3)	-230(3)	7122(1)	105(3)
N(16)	4266(3)	1214(3)	7323(1)	77(2)
C(1)	8159(5)	4202(6)	5331(2)	154(4)
C(2)	8028(5)	4396(6)	5170(2)	133(4)

C(3)	8356(5)	4439(6)	5007(2)	175(5)
C(4)	8235(7)	4614(8)	4832(3)	181(5)
C(5)	8311(8)	5073(8)	4871(3)	223(7)
C(6)	7955(8)	5103(7)	5041(3)	205(6)
C(7)	8138(7)	4898(6)	5214(3)	187(5)
C(8)	7488(4)	4050(5)	5114(2)	133(4)
C(9)	7717(6)	4248(6)	4776(2)	173(5)
C(10)	7380(8)	4713(8)	4967(3)	207(6)
C(11)	7336(6)	4202(7)	4929(2)	171(5)
C(12)	7954(4)	4475(5)	5646(2)	129(4)
C(13)	7578(4)	4240(4)	5770(2)	101(3)
C(14)	7409(4)	4364(4)	5938(2)	95(3)
C(15)	7672(5)	4791(5)	6044(2)	149(5)
C(16)	7459(5)	4866(5)	6204(2)	140(5)
C(17)	6991(4)	4502(4)	6262(2)	98(3)
C(18)	6761(4)	4058(4)	6160(1)	85(3)
C(19)	6729(4)	4583(4)	6416(2)	100(3)
C(20)	6973(5)	4974(5)	6535(2)	159(6)
C(21)	6695(5)	5053(5)	6681(2)	158(6)
C(22)	6209(4)	4751(4)	6711(1)	87(3)
C(23)	5968(5)	4370(5)	6580(2)	141(5)
C(24)	6207(5)	4288(5)	6442(2)	145(5)
C(25)	5924(4)	4844(4)	6859(2)	89(3)
C(26)	5415(5)	4701(5)	6847(2)	124(4)
C(27)	5192(4)	4835(4)	6980(2)	108(4)

C(28)	5461(4)	5118(3)	7122(2)	82(3)
C(29)	6179(3)	5118(3)	7020(1)	74(2)
C(30)	5281(4)	5305(4)	7280(2)	89(3)
C(31)	4850(4)	5305(4)	7329(2)	117(4)
C(32)	4628(4)	5676(5)	7603(2)	151(4)
C(33)	4631(5)	6157(6)	7540(3)	155(4)
C(34)	4286(6)	6216(6)	7683(3)	208(6)
C(35)	4263(8)	6718(9)	7630(4)	229(7)
C(36)	4077(9)	6655(10)	7434(4)	262(8)
C(37)	4480(8)	6636(8)	7284(4)	238(7)
C(38)	4459(6)	6133(7)	7331(3)	193(5)
C(39)	5140(5)	6613(5)	7558(3)	174(5)
C(40)	4763(7)	7137(7)	7660(3)	215(7)
C(41)	4990(9)	7086(9)	7329(4)	238(7)
C(42)	5126(8)	7099(7)	7525(4)	212(6)
C(43)	6989(4)	6874(4)	7961(2)	126(4)
C(44)	6653(5)	7026(5)	8048(2)	134(4)
C(45)	6972(6)	7527(5)	8152(3)	191(6)
C(46)	6645(9)	7735(8)	8236(4)	215(7)
C(47)	6340(10)	7406(10)	8357(4)	255(8)
C(48)	5845(9)	6793(9)	8264(4)	231(7)
C(49)	6271(7)	6630(6)	8185(3)	194(6)
C(50)	6348(5)	7107(5)	7899(2)	156(4)
C(51)	6339(7)	7768(7)	8093(3)	213(6)
C(52)	5675(9)	6943(9)	8108(4)	226(7)

C(53)	6018(7)	7321(7)	7964(3)	195(6)
C(54)	6570(5)	5926(5)	7926(2)	120(4)
C(55)	6388(4)	5621(4)	7773(2)	96(3)
C(56)	6137(4)	5096(4)	7717(2)	89(3)
C(57)	5991(6)	4709(5)	7849(2)	144(5)
C(58)	5759(6)	4214(4)	7775(2)	137(5)
C(59)	5660(4)	4121(4)	7590(2)	91(3)
C(60)	5822(3)	4537(3)	7471(1)	74(3)
C(61)	5391(4)	3611(4)	7511(2)	89(3)
C(62)	5404(5)	3212(4)	7601(2)	136(5)
C(63)	5104(5)	2722(4)	7536(2)	119(4)
C(64)	4800(4)	2606(4)	7385(2)	91(3)
C(65)	4813(5)	2990(4)	7290(2)	122(4)
C(66)	5121(5)	3493(4)	7353(2)	120(4)
C(67)	4463(4)	2068(4)	7326(2)	89(3)
C(68)	4570(3)	1705(3)	7370(1)	78(3)
C(69)	4018(4)	1923(4)	7226(2)	123(4)
C(70)	3710(4)	1436(4)	7180(2)	123(4)
C(71)	3836(4)	1081(4)	7229(2)	93(3)
C(72)	3547(4)	547(4)	7190(2)	89(3)
C(73)	3108(4)	220(4)	7104(2)	104(3)
C(74)	2652(4)	-719(4)	7056(2)	128(4)
C(75)	2203(5)	-961(4)	7179(2)	134(4)
C(76)	2352(5)	-1051(5)	7377(2)	157(4)
C(77)	1869(6)	-1350(6)	7494(3)	192(5)

C(78)	1523(6)	-1861(6)	7399(3)	210(6)
C(79)	1370(6)	-1768(7)	7206(4)	206(6)
C(80)	1837(5)	-1473(5)	7087(3)	180(5)
C(81)	1950(5)	-666(5)	7201(2)	162(5)
C(82)	1617(7)	-1062(7)	7518(3)	208(6)
C(83)	1087(6)	-1479(7)	7237(3)	218(6)
C(84)	1462(6)	-941(7)	7336(3)	194(5)
P(2)	8224(3)	7339(3)	7630(1)	245(3)
F(7)	8171(6)	7277(7)	7846(3)	338(7)
F(8)	7928(5)	7620(5)	7641(2)	280(5)
F(9)	8737(4)	7871(5)	7645(2)	288(6)
F(10)	8215(6)	7386(6)	7398(3)	316(6)
F(11)	8508(6)	7069(6)	7585(3)	319(6)
F(12)	7780(5)	6800(5)	7616(2)	283(6)
P(1)	8064(3)	7823(2)	6647(1)	203(2)
F(1)	8086(6)	7516(6)	6825(2)	301(6)
F(2)	7628(6)	7868(6)	6732(2)	296(6)
F(3)	8443(6)	8307(5)	6762(2)	288(5)
F(4)	8087(5)	8208(4)	6494(2)	246(4)
F(5)	8527(5)	7850(5)	6566(2)	265(5)
F(6)	7816(7)	7395(6)	6500(3)	319(6)

Table S74: Bond lengths [Å] and angles [°] for **1**·12PF_6.

Co(1)-N(1)#1	1.940(9)
Co(1)-N(1)#2	1.940(9)
Co(1)-N(1)	1.940(9)
Co(1)-N(4)#2	2.037(8)
Co(1)-N(4)	2.037(8)
Co(1)-N(4)#1	2.037(8)
Co(2)-N(5)	1.895(7)
Co(2)-N(8)	1.978(8)
Co(2)-N(9)	1.881(8)
Co(2)-N(12)	1.934(8)
Co(2)-N(13)#1	1.893(8)
Co(2)-N(16)#1	1.983(7)
N(1)-N(2)	1.305(11)
N(1)-C(13)	1.378(12)
N(2)-N(3)	1.312(12)
N(3)-C(1)	1.518(16)
N(3)-C(12)	1.347(14)
N(4)-C(14)	1.339(12)
N(4)-C(18)	1.362(11)
N(5)-N(6)	1.314(9)
N(5)-C(30)	1.328(11)
N(6)-N(7)	1.352(11)
N(7)-C(31)	1.279(13)

N(7)-C(32)	1.485(14)
N(8)-C(28)	1.362(11)
N(8)-C(29)	1.326(10)
N(9)-N(10)	1.305(10)
N(9)-C(55)	1.383(12)
N(10)-N(11)	1.328(12)
N(11)-C(43)	1.439(13)
N(11)-C(54)	1.346(14)
N(12)-C(56)	1.345(12)
N(12)-C(60)	1.344(10)
N(13)-Co(2)#2	1.893(8)
N(13)-N(14)	1.317(9)
N(13)-C(72)	1.346(11)
N(14)-N(15)	1.340(11)
N(15)-C(73)	1.321(12)
N(15)-C(74)	1.470(13)
N(16)-Co(2)#2	1.983(7)
N(16)-C(68)	1.352(11)
N(16)-C(71)	1.343(11)
C(1)-C(2)	1.428(18)
C(2)-C(3)	1.496(17)
C(2)-C(7)	1.428(19)
C(2)-C(8)	1.500(16)
C(3)-C(4)	1.47(2)
C(4)-C(5)	1.33(2)

C(4)-C(9)	1.46(2)
C(5)-C(6)	1.65(2)
C(6)-C(7)	1.60(2)
C(6)-C(10)	1.64(2)
C(8)-C(11)	1.542(19)
C(9)-C(11)	1.544(19)
C(10)-C(11)	1.52(2)
C(12)-C(13)	1.336(14)
C(13)-C(14)	1.423(14)
C(14)-C(15)	1.363(14)
C(15)-C(16)	1.388(15)
C(16)-C(17)	1.364(14)
C(17)-C(18)	1.381(13)
C(17)-C(19)	1.445(14)
C(19)-C(20)	1.341(14)
C(19)-C(24)	1.397(15)
C(20)-C(21)	1.436(16)
C(21)-C(22)	1.315(15)
C(22)-C(23)	1.377(14)
C(22)-C(25)	1.477(14)
C(23)-C(24)	1.321(15)
C(25)-C(26)	1.392(14)
C(25)-C(29)	1.401(13)
C(26)-C(27)	1.338(14)
C(27)-C(28)	1.316(13)

C(28)-C(30)	1.485(14)
C(30)-C(31)	1.359(13)
C(32)-C(33)	1.53(2)
C(33)-C(34)	1.537(18)
C(33)-C(38)	1.57(2)
C(33)-C(39)	1.485(17)
C(34)-C(35)	1.61(3)
C(35)-C(36)	1.48(3)
C(35)-C(40)	1.43(2)
C(36)-C(37)	1.65(3)
C(37)-C(38)	1.54(2)
C(37)-C(41)	1.51(3)
C(39)-C(42)	1.52(2)
C(40)-C(42)	1.51(2)
C(41)-C(42)	1.45(3)
C(43)-C(44)	1.457(15)
C(44)-C(45)	1.532(16)
C(44)-C(49)	1.53(2)
C(44)-C(50)	1.504(18)
C(45)-C(46)	1.55(2)
C(46)-C(47)	1.30(3)
C(46)-C(51)	1.42(3)
C(47)-C(48)	1.84(3)
C(48)-C(49)	1.70(2)
C(48)-C(52)	1.39(3)

C(50)-C(53)	1.520(18)
C(51)-C(53)	1.53(2)
C(52)-C(53)	1.51(3)
C(54)-C(55)	1.361(14)
C(55)-C(56)	1.443(14)
C(56)-C(57)	1.395(14)
C(57)-C(58)	1.410(15)
C(58)-C(59)	1.349(15)
C(59)-C(60)	1.395(12)
C(59)-C(61)	1.459(14)
C(61)-C(62)	1.393(14)
C(61)-C(66)	1.330(14)
C(62)-C(63)	1.385(15)
C(63)-C(64)	1.342(14)
C(64)-C(65)	1.339(13)
C(64)-C(67)	1.498(14)
C(65)-C(66)	1.411(14)
C(67)-C(68)	1.339(12)
C(67)-C(69)	1.394(14)
C(69)-C(70)	1.343(14)
C(70)-C(71)	1.365(13)
C(71)-C(72)	1.439(13)
C(72)-C(73)	1.352(13)
C(74)-C(75)	1.472(17)
C(75)-C(76)	1.548(19)

C(75)-C(80)	1.538(18)
C(75)-C(81)	1.459(17)
C(76)-C(77)	1.53(2)
C(77)-C(78)	1.54(2)
C(77)-C(82)	1.44(2)
C(78)-C(79)	1.52(2)
C(79)-C(80)	1.51(2)
C(79)-C(83)	1.53(2)
C(81)-C(84)	1.61(2)
C(82)-C(84)	1.48(2)
C(83)-C(84)	1.62(2)
P(2)-F(7)	1.54(2)
P(2)-F(8)	1.529(15)
P(2)-F(9)	1.599(14)
P(2)-F(10)	1.652(19)
P(2)-F(11)	1.496(17)
P(2)-F(12)	1.524(13)
P(1)-F(1)	1.591(16)
P(1)-F(2)	1.526(15)
P(1)-F(3)	1.575(16)
P(1)-F(4)	1.580(13)
P(1)-F(5)	1.492(13)
P(1)-F(6)	1.543(18)
N(1)#1-Co(1)-N(1)#2	94.5(3)

N(1)#1-Co(1)-N(1)	94.5(3)
N(1)#2-Co(1)-N(1)	94.5(3)
N(1)#2-Co(1)-N(4)	92.7(3)
N(1)#1-Co(1)-N(4)#2	92.7(3)
N(1)#2-Co(1)-N(4)#1	171.0(3)
N(1)#2-Co(1)-N(4)#2	79.6(4)
N(1)#1-Co(1)-N(4)	171.0(3)
N(1)-Co(1)-N(4)#1	92.7(3)
N(1)-Co(1)-N(4)#2	171.0(3)
N(1)-Co(1)-N(4)	79.6(4)
N(1)#1-Co(1)-N(4)#1	79.6(3)
N(4)#1-Co(1)-N(4)#2	93.8(3)
N(4)#1-Co(1)-N(4)	93.8(3)
N(4)-Co(1)-N(4)#2	93.8(3)
N(5)-Co(2)-N(8)	81.8(3)
N(5)-Co(2)-N(12)	90.2(3)
N(5)-Co(2)-N(16)#1	173.6(3)
N(8)-Co(2)-N(16)#1	94.3(3)
N(9)-Co(2)-N(5)	92.4(3)
N(9)-Co(2)-N(8)	173.0(3)
N(9)-Co(2)-N(12)	82.4(3)
N(9)-Co(2)-N(13)#1	93.3(3)
N(9)-Co(2)-N(16)#1	91.8(3)
N(12)-Co(2)-N(8)	93.8(3)
N(12)-Co(2)-N(16)#1	95.2(3)

N(13)#1-Co(2)-N(5)	93.2(3)
N(13)#1-Co(2)-N(8)	90.8(3)
N(13)#1-Co(2)-N(12)	174.6(3)
N(13)#1-Co(2)-N(16)#1	81.7(3)
N(2)-N(1)-Co(1)	134.2(8)
N(2)-N(1)-C(13)	109.6(9)
C(13)-N(1)-Co(1)	116.0(7)
N(1)-N(2)-N(3)	106.2(9)
N(2)-N(3)-C(1)	120.1(10)
N(2)-N(3)-C(12)	111.9(10)
C(12)-N(3)-C(1)	127.8(12)
C(14)-N(4)-Co(1)	115.6(7)
C(14)-N(4)-C(18)	119.2(8)
C(18)-N(4)-Co(1)	125.2(7)
N(6)-N(5)-Co(2)	132.4(7)
N(6)-N(5)-C(30)	111.1(8)
C(30)-N(5)-Co(2)	116.5(7)
N(5)-N(6)-N(7)	103.2(8)
N(6)-N(7)-C(32)	116.3(10)
C(31)-N(7)-N(6)	113.1(10)
C(31)-N(7)-C(32)	130.5(11)
C(28)-N(8)-Co(2)	115.6(6)
C(29)-N(8)-Co(2)	126.0(6)
C(29)-N(8)-C(28)	118.4(8)
N(10)-N(9)-Co(2)	133.0(8)

N(10)-N(9)-C(55)	110.5(9)
C(55)-N(9)-Co(2)	116.5(7)
N(9)-N(10)-N(11)	106.0(9)
N(10)-N(11)-C(43)	118.4(10)
N(10)-N(11)-C(54)	111.9(10)
C(54)-N(11)-C(43)	129.7(11)
C(56)-N(12)-Co(2)	114.8(6)
C(60)-N(12)-Co(2)	128.2(7)
C(60)-N(12)-C(56)	116.9(8)
N(14)-N(13)-Co(2)#2	133.1(7)
N(14)-N(13)-C(72)	110.8(8)
C(72)-N(13)-Co(2)#2	116.0(7)
N(13)-N(14)-N(15)	104.3(8)
N(14)-N(15)-C(74)	119.9(9)
C(73)-N(15)-N(14)	112.4(9)
C(73)-N(15)-C(74)	127.7(9)
C(68)-N(16)-Co(2)#2	127.0(6)
C(71)-N(16)-Co(2)#2	114.1(6)
C(71)-N(16)-C(68)	118.9(8)
C(2)-C(1)-N(3)	118.2(11)
C(1)-C(2)-C(3)	110.0(13)
C(1)-C(2)-C(7)	107.8(14)
C(1)-C(2)-C(8)	110.9(12)
C(3)-C(2)-C(8)	107.8(11)
C(7)-C(2)-C(3)	106.7(13)

C(7)-C(2)-C(8)	113.6(14)
C(4)-C(3)-C(2)	114.2(15)
C(5)-C(4)-C(3)	104.9(17)
C(5)-C(4)-C(9)	114.4(19)
C(9)-C(4)-C(3)	108.9(16)
C(4)-C(5)-C(6)	116.0(17)
C(7)-C(6)-C(5)	99.8(16)
C(7)-C(6)-C(10)	114.7(16)
C(10)-C(6)-C(5)	102.7(15)
C(2)-C(7)-C(6)	108.1(15)
C(2)-C(8)-C(11)	113.9(12)
C(4)-C(9)-C(11)	111.0(13)
C(11)-C(10)-C(6)	108.4(16)
C(8)-C(11)-C(9)	107.4(13)
C(10)-C(11)-C(8)	105.7(14)
C(10)-C(11)-C(9)	110.8(14)
C(13)-C(12)-N(3)	105.4(12)
N(1)-C(13)-C(14)	115.5(10)
C(12)-C(13)-N(1)	106.8(10)
C(12)-C(13)-C(14)	137.5(11)
N(4)-C(14)-C(13)	113.3(9)
N(4)-C(14)-C(15)	119.6(10)
C(15)-C(14)-C(13)	126.6(10)
C(14)-C(15)-C(16)	120.6(11)
C(17)-C(16)-C(15)	120.1(11)

C(16)-C(17)-C(18)	116.9(10)
C(16)-C(17)-C(19)	122.2(10)
C(18)-C(17)-C(19)	120.8(10)
N(4)-C(18)-C(17)	122.9(9)
C(20)-C(19)-C(17)	121.2(11)
C(20)-C(19)-C(24)	116.0(11)
C(24)-C(19)-C(17)	122.6(10)
C(19)-C(20)-C(21)	119.5(12)
C(22)-C(21)-C(20)	123.6(12)
C(21)-C(22)-C(23)	115.0(11)
C(21)-C(22)-C(25)	123.1(10)
C(23)-C(22)-C(25)	121.5(10)
C(24)-C(23)-C(22)	123.3(11)
C(23)-C(24)-C(19)	122.4(11)
C(26)-C(25)-C(22)	124.7(10)
C(26)-C(25)-C(29)	115.7(10)
C(29)-C(25)-C(22)	119.5(9)
C(27)-C(26)-C(25)	121.4(11)
C(28)-C(27)-C(26)	119.9(10)
N(8)-C(28)-C(30)	110.1(9)
C(27)-C(28)-N(8)	122.4(9)
C(27)-C(28)-C(30)	127.4(9)
N(8)-C(29)-C(25)	122.2(9)
N(5)-C(30)-C(28)	115.9(9)
N(5)-C(30)-C(31)	106.8(10)

C(31)-C(30)-C(28)	137.3(11)
N(7)-C(31)-C(30)	105.7(11)
N(7)-C(32)-C(33)	112.4(11)
C(32)-C(33)-C(34)	104.0(14)
C(32)-C(33)-C(38)	113.1(12)
C(34)-C(33)-C(38)	113.6(13)
C(39)-C(33)-C(32)	112.2(12)
C(39)-C(33)-C(34)	106.9(12)
C(39)-C(33)-C(38)	107.1(16)
C(33)-C(34)-C(35)	108.3(16)
C(36)-C(35)-C(34)	107.0(18)
C(40)-C(35)-C(34)	106.2(16)
C(40)-C(35)-C(36)	115(3)
C(35)-C(36)-C(37)	111.5(18)
C(38)-C(37)-C(36)	103.7(18)
C(41)-C(37)-C(36)	106.3(18)
C(41)-C(37)-C(38)	111.7(17)
C(37)-C(38)-C(33)	108.2(15)
C(33)-C(39)-C(42)	112.0(13)
C(35)-C(40)-C(42)	109.4(16)
C(42)-C(41)-C(37)	112(3)
C(40)-C(42)-C(39)	111.0(17)
C(41)-C(42)-C(39)	105.4(17)
C(41)-C(42)-C(40)	113.6(18)
N(11)-C(43)-C(44)	116.5(9)

C(43)-C(44)-C(45)	108.9(11)
C(43)-C(44)-C(49)	113.3(12)
C(43)-C(44)-C(50)	110.4(12)
C(45)-C(44)-C(49)	109.5(13)
C(50)-C(44)-C(45)	108.1(12)
C(50)-C(44)-C(49)	106.5(13)
C(44)-C(45)-C(46)	112.3(13)
C(47)-C(46)-C(45)	107(2)
C(47)-C(46)-C(51)	107(2)
C(51)-C(46)-C(45)	109.7(18)
C(46)-C(47)-C(48)	117(2)
C(49)-C(48)-C(47)	93.3(14)
C(52)-C(48)-C(47)	102(2)
C(52)-C(48)-C(49)	107.2(19)
C(44)-C(49)-C(48)	111.7(15)
C(44)-C(50)-C(53)	117.2(13)
C(46)-C(51)-C(53)	121.4(17)
C(48)-C(52)-C(53)	124(2)
C(50)-C(53)-C(51)	107.4(13)
C(52)-C(53)-C(50)	103.3(16)
C(52)-C(53)-C(51)	99.4(18)
N(11)-C(54)-C(55)	105.5(12)
N(9)-C(55)-C(56)	111.9(10)
C(54)-C(55)-N(9)	106.0(10)
C(54)-C(55)-C(56)	142.1(12)

N(12)-C(56)-C(55)	114.3(10)
N(12)-C(56)-C(57)	124.2(9)
C(57)-C(56)-C(55)	121.5(10)
C(56)-C(57)-C(58)	115.7(11)
C(59)-C(58)-C(57)	121.9(11)
C(58)-C(59)-C(60)	117.4(10)
C(58)-C(59)-C(61)	123.1(10)
C(60)-C(59)-C(61)	119.5(10)
N(12)-C(60)-C(59)	123.9(9)
C(62)-C(61)-C(59)	120.6(10)
C(66)-C(61)-C(59)	123.7(10)
C(66)-C(61)-C(62)	115.7(10)
C(63)-C(62)-C(61)	120.0(11)
C(64)-C(63)-C(62)	123.0(11)
C(63)-C(64)-C(67)	121.3(10)
C(65)-C(64)-C(63)	117.3(10)
C(65)-C(64)-C(67)	121.4(10)
C(64)-C(65)-C(66)	120.2(11)
C(61)-C(66)-C(65)	123.2(10)
C(68)-C(67)-C(64)	121.5(10)
C(68)-C(67)-C(69)	117.3(9)
C(69)-C(67)-C(64)	121.2(9)
C(67)-C(68)-N(16)	122.9(9)
C(70)-C(69)-C(67)	120.5(10)
C(69)-C(70)-C(71)	119.9(10)

N(16)-C(71)-C(70)	120.5(9)
N(16)-C(71)-C(72)	113.1(8)
C(70)-C(71)-C(72)	126.4(10)
N(13)-C(72)-C(71)	115.0(9)
N(13)-C(72)-C(73)	107.0(9)
C(73)-C(72)-C(71)	137.9(10)
N(15)-C(73)-C(72)	105.4(9)
N(15)-C(74)-C(75)	115.8(11)
C(74)-C(75)-C(76)	110.1(11)
C(74)-C(75)-C(80)	106.3(12)
C(80)-C(75)-C(76)	109.5(12)
C(81)-C(75)-C(74)	113.7(12)
C(81)-C(75)-C(76)	107.6(14)
C(81)-C(75)-C(80)	109.7(11)
C(77)-C(76)-C(75)	108.6(12)
C(76)-C(77)-C(78)	109.5(16)
C(82)-C(77)-C(76)	110.4(15)
C(82)-C(77)-C(78)	111.4(16)
C(79)-C(78)-C(77)	109.0(16)
C(78)-C(79)-C(83)	107(2)
C(80)-C(79)-C(78)	109.3(16)
C(80)-C(79)-C(83)	112.0(16)
C(79)-C(80)-C(75)	111.0(15)
C(75)-C(81)-C(84)	112.4(12)
C(77)-C(82)-C(84)	113(2)

C(79)-C(83)-C(84)	109.5(14)
C(81)-C(84)-C(83)	105.6(15)
C(82)-C(84)-C(81)	108.6(14)
C(82)-C(84)-C(83)	106.1(16)
F(7)-P(2)-F(9)	92.5(10)
F(7)-P(2)-F(10)	173.8(11)
F(8)-P(2)-F(7)	87.8(10)
F(8)-P(2)-F(9)	88.7(9)
F(8)-P(2)-F(10)	87.8(12)
F(9)-P(2)-F(10)	91.8(9)
F(11)-P(2)-F(7)	101.4(14)
F(11)-P(2)-F(8)	170.7(13)
F(11)-P(2)-F(9)	91.9(8)
F(11)-P(2)-F(10)	82.9(10)
F(11)-P(2)-F(12)	80.8(9)
F(12)-P(2)-F(7)	87.1(9)
F(12)-P(2)-F(8)	98.8(8)
F(12)-P(2)-F(9)	172.5(10)
F(12)-P(2)-F(10)	89.2(9)
F(2)-P(1)-F(1)	92.0(8)
F(2)-P(1)-F(3)	88.6(9)
F(2)-P(1)-F(4)	85.5(7)
F(2)-P(1)-F(6)	105.2(10)
F(3)-P(1)-F(1)	85.0(10)
F(3)-P(1)-F(4)	85.5(7)

F(4)-P(1)-F(1)	170.3(10)
F(5)-P(1)-F(1)	91.2(9)
F(5)-P(1)-F(2)	172.8(9)
F(5)-P(1)-F(3)	85.2(8)
F(5)-P(1)-F(4)	90.2(7)
F(5)-P(1)-F(6)	80.4(9)
F(6)-P(1)-F(1)	100.7(9)
F(6)-P(1)-F(3)	164.7(10)
F(6)-P(1)-F(4)	89.1(9)

Symmetry transformations used to generate equivalent atoms:

#1 -x+y+1,-x+1,z   #2 -y+1,x-y,z

Table S75: Anisotropic displacement parameters ($Å^2 x 10^3$) for **1**·12PF_6. The anisotropic displacement factor exponent takes the form: $-2p^2[h^2 a^{*2}U^{11} + ... + 2 h k a^* b^* U^{12}]$

	U^{11}	U^{22}	U^{33}	U^{23}	U^{13}	U^{12}
Co(1)	86(1)	86(1)	78(2)	0	0	43(1)
Co(2)	72(1)	60(1)	93(1)	-7(1)	11(1)	39(1)
N(1)	90(5)	118(6)	88(5)	-30(4)	-6(4)	59(4)
N(2)	94(6)	151(7)	98(6)	-37(5)	-10(5)	74(5)
N(3)	91(6)	166(8)	105(6)	-44(5)	8(5)	56(6)
N(4)	92(5)	94(5)	71(5)	-14(4)	-2(4)	44(4)
N(5)	67(4)	58(4)	102(5)	-5(4)	16(4)	37(4)
N(6)	90(5)	82(5)	114(6)	-14(5)	23(5)	51(5)
N(7)	84(5)	114(7)	149(7)	-39(5)	16(5)	57(5)
N(8)	71(4)	53(4)	94(5)	-7(4)	5(4)	36(4)
N(9)	79(5)	67(5)	108(5)	-24(4)	-2(4)	44(4)
N(10)	86(5)	73(5)	127(6)	-37(5)	-1(5)	38(4)
N(11)	104(6)	95(5)	120(6)	-49(5)	-14(5)	56(5)
N(12)	84(5)	66(4)	81(5)	-15(4)	5(4)	45(4)
N(13)	65(5)	58(5)	108(6)	13(4)	7(4)	25(4)
N(14)	76(5)	57(5)	130(7)	5(4)	-2(5)	21(4)
N(15)	86(5)	61(5)	149(8)	-1(5)	-21(5)	21(4)
N(16)	61(5)	58(4)	106(6)	19(4)	7(4)	25(4)
C(1)	110(8)	220(11)	124(7)	-43(7)	22(6)	74(8)
C(2)	92(7)	161(9)	110(7)	-37(6)	27(6)	37(6)

C(3)	126(9)	227(12)	131(8)	-43(8)	40(7)	59(8)
C(4)	144(9)	216(12)	140(9)	-43(9)	24(8)	57(9)
C(5)	208(13)	223(12)	179(12)	-35(9)	34(10)	64(10)
C(6)	230(12)	190(11)	170(11)	-13(9)	41(9)	86(10)
C(7)	188(12)	177(10)	153(10)	-38(8)	33(9)	59(8)
C(8)	103(7)	156(9)	115(8)	-21(7)	19(6)	46(6)
C(9)	157(9)	209(12)	130(9)	-12(8)	23(7)	73(9)
C(10)	232(12)	226(12)	165(12)	0(10)	32(10)	115(10)
C(11)	152(9)	210(12)	134(9)	0(8)	23(7)	76(9)
C(12)	103(7)	142(8)	106(8)	-35(6)	25(6)	35(6)
C(13)	90(6)	118(7)	87(6)	-22(5)	5(5)	47(5)
C(14)	87(6)	95(6)	87(6)	-20(5)	11(5)	33(5)
C(15)	118(8)	123(8)	140(9)	-53(7)	43(7)	9(7)
C(16)	118(8)	121(8)	129(9)	-41(7)	31(6)	21(6)
C(17)	105(7)	90(6)	86(6)	-18(5)	15(5)	39(5)
C(18)	90(6)	87(6)	76(6)	-10(5)	0(5)	43(5)
C(19)	112(7)	94(7)	89(7)	-23(5)	13(5)	46(6)
C(20)	117(8)	156(10)	146(10)	-74(8)	28(7)	24(7)
C(21)	129(8)	149(9)	153(10)	-71(8)	28(7)	39(7)
C(22)	107(7)	84(6)	79(6)	-10(5)	3(5)	55(5)
C(23)	104(7)	151(9)	135(9)	-72(8)	12(6)	41(7)
C(24)	110(7)	155(9)	134(9)	-79(8)	17(6)	39(7)
C(25)	102(6)	79(6)	90(6)	-11(5)	5(5)	47(5)
C(26)	109(7)	150(9)	124(8)	-54(7)	-15(6)	72(7)
C(27)	78(6)	132(9)	121(7)	-48(6)	-13(5)	57(6)

C(28)	69(5)	75(6)	104(6)	-16(5)	1(4)	39(5)
C(29)	79(6)	69(6)	85(6)	-5(4)	10(4)	44(5)
C(30)	72(6)	82(6)	116(6)	-24(5)	5(4)	41(5)
C(31)	78(6)	123(9)	157(8)	-50(7)	2(6)	55(6)
C(32)	101(8)	170(9)	197(10)	-54(8)	30(7)	80(7)
C(33)	91(7)	154(8)	243(11)	-65(8)	16(7)	78(7)
C(34)	148(10)	214(11)	302(13)	-66(10)	48(9)	121(9)
C(35)	191(12)	223(13)	326(16)	-80(12)	18(12)	145(10)
C(36)	229(14)	278(17)	354(16)	-83(13)	-8(12)	182(13)
C(37)	228(14)	233(13)	326(14)	-53(11)	-8(11)	171(11)
C(38)	171(11)	198(11)	262(11)	-62(9)	-8(9)	131(10)
C(39)	114(8)	156(9)	278(14)	-53(9)	4(8)	87(7)
C(40)	176(11)	198(12)	327(16)	-62(11)	16(11)	135(10)
C(41)	239(14)	218(13)	324(16)	-48(11)	-2(11)	164(11)
C(42)	186(12)	182(10)	317(15)	-50(10)	5(11)	129(9)
C(43)	122(7)	98(7)	152(9)	-74(6)	-21(6)	51(6)
C(44)	123(8)	103(7)	168(10)	-82(7)	-16(6)	52(6)
C(45)	176(10)	152(9)	241(13)	-129(9)	-38(9)	79(8)
C(46)	217(14)	188(13)	254(15)	-124(11)	-23(10)	112(11)
C(47)	261(16)	252(14)	283(15)	-91(11)	12(11)	153(12)
C(48)	225(13)	237(14)	261(16)	-66(11)	40(10)	138(11)
C(49)	204(11)	188(10)	198(12)	-48(8)	27(9)	105(9)
C(50)	153(10)	135(9)	189(10)	-77(8)	-15(7)	79(8)
C(51)	224(14)	188(11)	258(15)	-119(10)	-15(10)	127(10)
C(52)	230(13)	220(14)	275(16)	-82(10)	26(10)	148(11)

C(53)	200(12)	172(11)	250(14)	-105(9)	-12(9)	121(10)
C(54)	147(9)	103(6)	114(7)	-45(5)	-23(6)	66(7)
C(55)	114(7)	78(6)	104(6)	-24(4)	-5(5)	54(5)
C(56)	116(7)	75(6)	89(6)	-14(4)	-3(5)	58(5)
C(57)	227(13)	94(7)	94(7)	-6(5)	-13(7)	67(7)
C(58)	220(13)	82(7)	96(7)	4(5)	-9(7)	66(7)
C(59)	115(7)	65(5)	89(6)	3(4)	4(5)	42(5)
C(60)	86(6)	67(5)	79(6)	-4(4)	11(5)	45(5)
C(61)	108(7)	65(5)	93(7)	5(5)	3(5)	43(5)
C(62)	181(11)	75(6)	142(9)	0(6)	-43(8)	56(6)
C(63)	159(9)	60(6)	133(8)	5(5)	-37(7)	52(6)
C(64)	96(7)	65(5)	114(8)	16(5)	1(5)	42(5)
C(65)	158(9)	63(6)	135(8)	6(5)	-49(7)	47(6)
C(66)	166(10)	68(6)	127(8)	0(5)	-37(7)	59(6)
C(67)	88(6)	64(5)	113(7)	17(5)	7(5)	36(5)
C(68)	62(5)	50(5)	107(7)	19(5)	6(5)	18(4)
C(69)	111(7)	67(6)	197(11)	13(6)	-31(7)	49(5)
C(70)	93(7)	65(6)	212(12)	7(6)	-41(7)	40(5)
C(71)	69(6)	56(5)	147(9)	10(5)	-18(5)	25(4)
C(72)	61(5)	56(5)	139(8)	11(5)	-6(5)	21(4)
C(73)	70(6)	64(6)	161(9)	8(5)	-23(6)	20(5)
C(74)	87(6)	71(6)	189(9)	-8(6)	-33(6)	11(5)
C(75)	79(7)	80(7)	212(9)	6(6)	-16(6)	15(5)
C(76)	109(8)	109(8)	211(10)	22(7)	-11(7)	23(7)
C(77)	131(10)	143(10)	249(12)	37(8)	12(8)	30(7)

C(78)	135(11)	139(10)	289(14)	20(9)	1(10)	17(8)
C(79)	117(10)	131(10)	296(14)	4(9)	-7(9)	7(7)
C(80)	105(8)	104(8)	258(12)	-18(7)	-22(8)	-2(6)
C(81)	112(8)	122(8)	233(12)	22(8)	-7(8)	46(7)
C(82)	152(12)	166(12)	265(13)	39(10)	19(10)	48(9)
C(83)	130(10)	167(11)	301(16)	12(10)	-12(10)	33(8)
C(84)	120(9)	165(11)	280(13)	29(9)	21(9)	57(8)
P(2)	156(5)	231(7)	284(8)	-25(6)	57(5)	48(5)
F(7)	274(13)	376(16)	317(10)	-15(9)	45(9)	128(12)
F(8)	197(9)	257(10)	358(14)	-32(9)	30(9)	92(8)
F(9)	184(8)	250(9)	353(14)	-31(9)	34(8)	51(7)
F(10)	280(13)	249(12)	319(10)	-25(9)	44(9)	58(9)
F(11)	232(10)	300(12)	405(16)	-43(11)	54(10)	118(9)
F(12)	189(8)	232(9)	343(14)	-22(8)	56(8)	42(7)
P(1)	201(5)	140(4)	246(7)	48(4)	79(5)	69(4)
F(1)	340(14)	260(11)	323(11)	90(9)	102(10)	165(11)
F(2)	298(11)	315(13)	294(13)	80(10)	112(9)	168(10)
F(3)	324(12)	223(9)	310(12)	18(8)	1(9)	131(8)
F(4)	288(11)	223(9)	243(9)	52(7)	68(8)	140(8)
F(5)	241(9)	252(10)	310(12)	49(9)	70(8)	129(8)
F(6)	311(13)	253(10)	393(14)	-22(9)	1(10)	141(9)

Table S76: Hydrogen coordinates (x 10^4) and isotropic displacement parameters ($Å^2$x 10^3) for **1**·12PF_6.

	x	y	z	U(eq)
H(1A)	8514	4429	5357	185
H(1B)	8122	3878	5296	185
H(3A)	8706	4673	5040	210
H(3B)	8325	4111	4984	210
H(4)	8468	4639	4732	218
H(5A)	8254	5214	4758	268
H(5B)	8663	5287	4906	268
H(6)	8016	5447	5061	246
H(7A)	8499	5115	5234	224
H(7B)	7963	4896	5329	224
H(8A)	7269	4044	5214	160
H(8B)	7433	3710	5101	160
H(9A)	7688	3920	4756	208
H(9B)	7637	4354	4659	208
H(10A)	7136	4678	5062	248
H(10B)	7312	4843	4853	248
H(11)	6990	3951	4893	205
H(12)	8220	4807	5651	154
H(15)	7998	5034	6008	179
H(16)	7636	5165	6272	168

H(18)	6447	3798	6200	102
H(20)	7320	5192	6522	191
H(21)	6869	5333	6759	189
H(23)	5619	4160	6590	169
H(24)	6020	4024	6358	174
H(26)	5225	4510	6745	149
H(27)	4849	4728	6971	130
H(29)	6516	5211	7036	89
H(31)	4544	5153	7264	141
H(32A)	4747	5718	7732	181
H(32B)	4283	5394	7600	181
H(34A)	4420	6246	7809	250
H(34B)	3949	5922	7679	250
H(35)	4026	6749	7714	274
H(36A)	4024	6934	7401	315
H(36B)	3754	6344	7424	315
H(37)	4382	6644	7153	286
H(38A)	4682	6082	7247	231
H(38B)	4118	5852	7314	231
H(39A)	5367	6593	7467	209
H(39B)	5273	6621	7683	209
H(40A)	4867	7134	7788	258
H(40B)	4765	7453	7639	258
H(41A)	5245	7074	7251	286
H(41B)	4987	7394	7298	286

H(42)	5466	7389	7545	254
H(43A)	7211	7138	7875	151
H(43B)	7201	6855	8059	151
H(45A)	7216	7774	8065	229
H(45B)	7158	7477	8252	229
H(46)	6857	8065	8296	258
H(47A)	6175	7558	8426	306
H(47B)	6541	7342	8446	306
H(48)	5588	6562	8353	277
H(49A)	6451	6596	8291	233
H(49B)	6092	6305	8122	233
H(50A)	6132	6784	7838	187
H(50B)	6581	7332	7805	187
H(51A)	6560	8051	8013	256
H(51B)	6110	7857	8154	256
H(52A)	5463	7071	8156	271
H(52B)	5453	6636	8039	271
H(53)	5845	7392	7862	234
H(54)	6569	5828	8050	143
H(57)	6043	4773	7977	173
H(58)	5671	3944	7856	164
H(60)	5765	4477	7342	89
H(62)	5613	3275	7704	163
H(63)	5113	2461	7600	142
H(65)	4619	2926	7182	147

H(66)	5136	3754	7280	144
H(68)	4868	1795	7435	93
H(69)	3933	2165	7192	148
H(70)	3412	1341	7114	148
H(73)	2883	297	7044	125
H(74A)	2782	-949	7042	154
H(74B)	2547	-671	6933	154
H(76A)	2530	-1240	7367	188
H(76B)	2575	-729	7437	188
H(77)	1963	-1413	7619	230
H(78A)	1699	-2050	7386	253
H(78B)	1223	-2059	7475	253
H(79)	1145	-2093	7145	247
H(80A)	1741	-1417	6963	216
H(80B)	2007	-1668	7072	216
H(81A)	1847	-610	7079	194
H(81B)	2187	-338	7254	194
H(82A)	1841	-750	7583	250
H(82B)	1319	-1253	7595	250
H(83A)	795	-1676	7316	262
H(83B)	971	-1423	7117	262
H(84)	1299	-737	7354	233

Table S77: Torsion angles [°] for **1**·12PF_6.

Co(1)-N(1)-N(2)-N(3)	175.1(8)
Co(1)-N(1)-C(13)-C(12)	-176.1(8)
Co(1)-N(1)-C(13)-C(14)	1.2(12)
Co(1)-N(4)-C(14)-C(13)	-0.1(12)
Co(1)-N(4)-C(14)-C(15)	-172.0(10)
Co(1)-N(4)-C(18)-C(17)	178.3(7)
Co(2)-N(5)-N(6)-N(7)	177.7(6)
Co(2)-N(5)-C(30)-C(28)	-0.4(11)
Co(2)-N(5)-C(30)-C(31)	-179.2(7)
Co(2)-N(8)-C(28)-C(27)	-174.8(8)
Co(2)-N(8)-C(28)-C(30)	1.2(9)
Co(2)-N(8)-C(29)-C(25)	178.8(6)
Co(2)-N(9)-N(10)-N(11)	177.6(6)
Co(2)-N(9)-C(55)-C(54)	-179.0(7)
Co(2)-N(9)-C(55)-C(56)	1.1(10)
Co(2)-N(12)-C(56)-C(55)	-3.1(11)
Co(2)-N(12)-C(56)-C(57)	176.5(9)
Co(2)-N(12)-C(60)-C(59)	-175.9(7)
Co(2)#2-N(13)-C(72)-C(71)	-0.5(11)
Co(2)#2-N(13)-C(72)-C(73)	180.0(7)
Co(2)#2-N(16)-C(68)-C(67)	-178.7(7)
Co(2)#2-N(16)-C(71)-C(70)	179.2(9)
Co(2)#2-N(16)-C(71)-C(72)	-0.6(11)

N(1)-N(2)-N(3)-C(1)	-176.0(10)
N(1)-N(2)-N(3)-C(12)	0.1(13)
N(1)-C(13)-C(14)-N(4)	-0.7(14)
N(1)-C(13)-C(14)-C(15)	170.5(12)
N(2)-N(1)-C(13)-C(12)	0.1(12)
N(2)-N(1)-C(13)-C(14)	177.4(9)
N(2)-N(3)-C(1)-C(2)	90.8(16)
N(2)-N(3)-C(12)-C(13)	0.0(14)
N(3)-C(1)-C(2)-C(3)	-179.8(11)
N(3)-C(1)-C(2)-C(7)	64.2(17)
N(3)-C(1)-C(2)-C(8)	-60.7(17)
N(3)-C(12)-C(13)-N(1)	-0.1(13)
N(3)-C(12)-C(13)-C(14)	-176.5(13)
N(4)-C(14)-C(15)-C(16)	-10(2)
N(5)-Co(2)-N(9)-N(10)	-89.5(8)
N(5)-Co(2)-N(9)-C(55)	87.6(7)
N(5)-N(6)-N(7)-C(31)	2.0(11)
N(5)-N(6)-N(7)-C(32)	179.4(9)
N(5)-C(30)-C(31)-N(7)	1.5(12)
N(6)-N(5)-C(30)-C(28)	178.5(7)
N(6)-N(5)-C(30)-C(31)	-0.3(11)
N(6)-N(7)-C(31)-C(30)	-2.2(13)
N(6)-N(7)-C(32)-C(33)	96.4(13)
N(7)-C(32)-C(33)-C(34)	-179.1(11)
N(7)-C(32)-C(33)-C(38)	57.2(15)

N(7)-C(32)-C(33)-C(39)	-64.0(17)
N(8)-Co(2)-N(5)-N(6)	-177.8(8)
N(8)-Co(2)-N(5)-C(30)	0.8(7)
N(8)-C(28)-C(30)-N(5)	-0.5(12)
N(8)-C(28)-C(30)-C(31)	177.8(12)
N(9)-Co(2)-N(5)-N(6)	6.0(8)
N(9)-Co(2)-N(5)-C(30)	-175.4(7)
N(9)-N(10)-N(11)-C(43)	179.3(8)
N(9)-N(10)-N(11)-C(54)	0.7(11)
N(9)-C(55)-C(56)-N(12)	1.3(12)
N(9)-C(55)-C(56)-C(57)	-178.3(10)
N(10)-N(9)-C(55)-C(54)	-1.2(11)
N(10)-N(9)-C(55)-C(56)	178.9(8)
N(10)-N(11)-C(43)-C(44)	97.2(14)
N(10)-N(11)-C(54)-C(55)	-1.5(12)
N(11)-C(43)-C(44)-C(45)	178.4(13)
N(11)-C(43)-C(44)-C(49)	56.3(17)
N(11)-C(43)-C(44)-C(50)	-63.1(16)
N(11)-C(54)-C(55)-N(9)	1.6(12)
N(11)-C(54)-C(55)-C(56)	-178.6(13)
N(12)-Co(2)-N(5)-N(6)	88.3(8)
N(12)-Co(2)-N(5)-C(30)	-93.0(7)
N(12)-Co(2)-N(9)-N(10)	-179.4(8)
N(12)-Co(2)-N(9)-C(55)	-2.2(7)
N(12)-C(56)-C(57)-C(58)	1.7(18)

N(13)#1-Co(2)-N(5)-N(6)	-87.5(8)
N(13)#1-Co(2)-N(5)-C(30)	91.1(7)
N(13)#1-Co(2)-N(9)-N(10)	3.8(8)
N(13)#1-Co(2)-N(9)-C(55)	-179.0(7)
N(13)-N(14)-N(15)-C(73)	1.8(11)
N(13)-N(14)-N(15)-C(74)	-178.3(9)
N(13)-C(72)-C(73)-N(15)	0.3(12)
N(14)-N(13)-C(72)-C(71)	-179.6(8)
N(14)-N(13)-C(72)-C(73)	0.8(11)
N(14)-N(15)-C(73)-C(72)	-1.3(12)
N(14)-N(15)-C(74)-C(75)	98.5(12)
N(15)-C(74)-C(75)-C(76)	-58.7(14)
N(15)-C(74)-C(75)-C(80)	-177.2(11)
N(15)-C(74)-C(75)-C(81)	62.1(16)
N(16)#1-Co(2)-N(9)-N(10)	85.7(8)
N(16)#1-Co(2)-N(9)-C(55)	-97.2(7)
N(16)-C(71)-C(72)-N(13)	0.7(13)
C(1)-N(3)-C(12)-C(13)	175.7(12)
C(1)-C(2)-C(3)-C(4)	177.2(15)
C(1)-C(2)-C(7)-C(6)	-175.9(12)
C(1)-C(2)-C(8)-C(11)	-173.4(12)
C(2)-C(3)-C(4)-C(5)	61(2)
C(2)-C(3)-C(4)-C(9)	-61.5(19)
C(2)-C(8)-C(11)-C(9)	54.2(17)
C(2)-C(8)-C(11)-C(10)	-64.2(16)

C(3)-C(2)-C(7)-C(6)	66.0(17)
C(3)-C(2)-C(8)-C(11)	-53.0(17)
C(3)-C(4)-C(5)-C(6)	-61(2)
C(3)-C(4)-C(9)-C(11)	61(2)
C(4)-C(5)-C(6)-C(7)	62(2)
C(4)-C(5)-C(6)-C(10)	-56(3)
C(4)-C(9)-C(11)-C(8)	-57.4(19)
C(4)-C(9)-C(11)-C(10)	58(2)
C(5)-C(4)-C(9)-C(11)	-56(2)
C(5)-C(6)-C(7)-C(2)	-60.5(17)
C(5)-C(6)-C(10)-C(11)	54.9(19)
C(6)-C(10)-C(11)-C(8)	55.7(17)
C(6)-C(10)-C(11)-C(9)	-60.4(19)
C(7)-C(2)-C(3)-C(4)	-66.1(19)
C(7)-C(2)-C(8)-C(11)	65.0(17)
C(7)-C(6)-C(10)-C(11)	-52(2)
C(8)-C(2)-C(3)-C(4)	56.2(19)
C(8)-C(2)-C(7)-C(6)	-52.7(17)
C(9)-C(4)-C(5)-C(6)	58(3)
C(10)-C(6)-C(7)-C(2)	48.5(19)
C(12)-N(3)-C(1)-C(2)	-84.6(17)
C(12)-C(13)-C(14)-N(4)	175.5(14)
C(12)-C(13)-C(14)-C(15)	-13(2)
C(13)-N(1)-N(2)-N(3)	-0.1(11)
C(13)-C(14)-C(15)-C(16)	179.5(13)

C(14)-N(4)-C(18)-C(17)	-4.1(14)
C(14)-C(15)-C(16)-C(17)	3(2)
C(15)-C(16)-C(17)-C(18)	3.1(19)
C(15)-C(16)-C(17)-C(19)	-173.0(12)
C(16)-C(17)-C(18)-N(4)	-2.6(16)
C(16)-C(17)-C(19)-C(20)	-14.6(19)
C(16)-C(17)-C(19)-C(24)	160.3(13)
C(17)-C(19)-C(20)-C(21)	177.5(13)
C(17)-C(19)-C(24)-C(23)	-178.6(13)
C(18)-N(4)-C(14)-C(13)	-177.9(8)
C(18)-N(4)-C(14)-C(15)	10.1(16)
C(18)-C(17)-C(19)-C(20)	169.4(12)
C(18)-C(17)-C(19)-C(24)	-15.7(17)
C(19)-C(17)-C(18)-N(4)	173.6(9)
C(19)-C(20)-C(21)-C(22)	2(2)
C(20)-C(19)-C(24)-C(23)	-3(2)
C(20)-C(21)-C(22)-C(23)	-5(2)
C(20)-C(21)-C(22)-C(25)	-178.4(13)
C(21)-C(22)-C(23)-C(24)	4(2)
C(21)-C(22)-C(25)-C(26)	149.2(13)
C(21)-C(22)-C(25)-C(29)	-26.8(16)
C(22)-C(23)-C(24)-C(19)	0(2)
C(22)-C(25)-C(26)-C(27)	-174.3(10)
C(22)-C(25)-C(29)-N(8)	173.9(8)
C(23)-C(22)-C(25)-C(26)	-23.5(16)

C(23)-C(22)-C(25)-C(29)	160.5(11)
C(24)-C(19)-C(20)-C(21)	2(2)
C(25)-C(22)-C(23)-C(24)	177.4(13)
C(25)-C(26)-C(27)-C(28)	1.9(18)
C(26)-C(25)-C(29)-N(8)	-2.5(13)
C(26)-C(27)-C(28)-N(8)	-5.4(17)
C(26)-C(27)-C(28)-C(30)	179.4(11)
C(27)-C(28)-C(30)-N(5)	175.2(10)
C(27)-C(28)-C(30)-C(31)	-7(2)
C(28)-N(8)-C(29)-C(25)	-0.6(12)
C(28)-C(30)-C(31)-N(7)	-176.9(11)
C(29)-N(8)-C(28)-C(27)	4.7(13)
C(29)-N(8)-C(28)-C(30)	-179.3(7)
C(29)-C(25)-C(26)-C(27)	1.9(16)
C(30)-N(5)-N(6)-N(7)	-1.0(9)
C(31)-N(7)-C(32)-C(33)	-86.8(16)
C(32)-N(7)-C(31)-C(30)	-179.1(12)
C(32)-C(33)-C(34)-C(35)	179.9(14)
C(32)-C(33)-C(38)-C(37)	-179.9(12)
C(32)-C(33)-C(39)-C(42)	-170.8(15)
C(33)-C(34)-C(35)-C(36)	57(2)
C(33)-C(34)-C(35)-C(40)	-66(2)
C(33)-C(39)-C(42)-C(40)	57(2)
C(33)-C(39)-C(42)-C(41)	-66(2)
C(34)-C(33)-C(38)-C(37)	61.9(19)

C(34)-C(33)-C(39)-C(42)	-57(2)
C(34)-C(35)-C(36)-C(37)	-65(3)
C(34)-C(35)-C(40)-C(42)	64(2)
C(35)-C(36)-C(37)-C(38)	68(3)
C(35)-C(36)-C(37)-C(41)	-50(3)
C(35)-C(40)-C(42)-C(39)	-61(3)
C(35)-C(40)-C(42)-C(41)	58(3)
C(36)-C(35)-C(40)-C(42)	-54(2)
C(36)-C(37)-C(38)-C(33)	-60.8(19)
C(36)-C(37)-C(41)-C(42)	54(2)
C(37)-C(41)-C(42)-C(39)	61(2)
C(37)-C(41)-C(42)-C(40)	-61(2)
C(38)-C(33)-C(34)-C(35)	-56.8(19)
C(38)-C(33)-C(39)-C(42)	64.6(18)
C(38)-C(37)-C(41)-C(42)	-58(2)
C(39)-C(33)-C(34)-C(35)	61.0(19)
C(39)-C(33)-C(38)-C(37)	-55.8(15)
C(40)-C(35)-C(36)-C(37)	53(3)
C(41)-C(37)-C(38)-C(33)	53(2)
C(43)-N(11)-C(54)-C(55)	-179.8(10)
C(43)-C(44)-C(45)-C(46)	175.9(17)
C(43)-C(44)-C(49)-C(48)	-173.8(13)
C(43)-C(44)-C(50)-C(53)	-173.6(12)
C(44)-C(45)-C(46)-C(47)	62(3)
C(44)-C(45)-C(46)-C(51)	-54(3)

C(44)-C(50)-C(53)-C(51)	46(2)
C(44)-C(50)-C(53)-C(52)	-58(2)
C(45)-C(44)-C(49)-C(48)	64.4(18)
C(45)-C(44)-C(50)-C(53)	-54.6(18)
C(45)-C(46)-C(47)-C(48)	-68(3)
C(45)-C(46)-C(51)-C(53)	50(3)
C(46)-C(47)-C(48)-C(49)	65(3)
C(46)-C(47)-C(48)-C(52)	-43(3)
C(46)-C(51)-C(53)-C(50)	-45(3)
C(46)-C(51)-C(53)-C(52)	62(3)
C(47)-C(46)-C(51)-C(53)	-66(3)
C(47)-C(48)-C(49)-C(44)	-56.8(19)
C(47)-C(48)-C(52)-C(53)	47(2)
C(48)-C(52)-C(53)-C(50)	55(2)
C(48)-C(52)-C(53)-C(51)	-56(2)
C(49)-C(44)-C(45)-C(46)	-60(2)
C(49)-C(44)-C(50)-C(53)	63.0(16)
C(49)-C(48)-C(52)-C(53)	-50(3)
C(50)-C(44)-C(45)-C(46)	56(2)
C(50)-C(44)-C(49)-C(48)	-52.2(16)
C(51)-C(46)-C(47)-C(48)	50(3)
C(52)-C(48)-C(49)-C(44)	47(2)
C(54)-N(11)-C(43)-C(44)	-84.6(16)
C(54)-C(55)-C(56)-N(12)	-178.5(13)
C(54)-C(55)-C(56)-C(57)	2(2)

C(55)-N(9)-N(10)-N(11)	0.3(10)
C(55)-C(56)-C(57)-C(58)	-178.8(11)
C(56)-N(12)-C(60)-C(59)	-0.1(12)
C(56)-C(57)-C(58)-C(59)	-4(2)
C(57)-C(58)-C(59)-C(60)	3.7(19)
C(57)-C(58)-C(59)-C(61)	-176.0(12)
C(58)-C(59)-C(60)-N(12)	-1.8(15)
C(58)-C(59)-C(61)-C(62)	-26.7(17)
C(58)-C(59)-C(61)-C(66)	152.9(13)
C(59)-C(61)-C(62)-C(63)	173.0(11)
C(59)-C(61)-C(66)-C(65)	-172.0(11)
C(60)-N(12)-C(56)-C(55)	-179.5(8)
C(60)-N(12)-C(56)-C(57)	0.1(15)
C(60)-C(59)-C(61)-C(62)	153.7(10)
C(60)-C(59)-C(61)-C(66)	-26.7(16)
C(61)-C(59)-C(60)-N(12)	177.9(8)
C(61)-C(62)-C(63)-C(64)	1(2)
C(62)-C(61)-C(66)-C(65)	7.7(18)
C(62)-C(63)-C(64)-C(65)	3.5(19)
C(62)-C(63)-C(64)-C(67)	-177.0(11)
C(63)-C(64)-C(65)-C(66)	-2.6(17)
C(63)-C(64)-C(67)-C(68)	-24.7(16)
C(63)-C(64)-C(67)-C(69)	153.5(12)
C(64)-C(65)-C(66)-C(61)	-3.2(19)
C(64)-C(67)-C(68)-N(16)	177.8(9)

C(64)-C(67)-C(69)-C(70)	-177.5(11)
C(65)-C(64)-C(67)-C(68)	154.7(11)
C(65)-C(64)-C(67)-C(69)	-27.0(16)
C(66)-C(61)-C(62)-C(63)	-6.7(17)
C(67)-C(64)-C(65)-C(66)	177.9(11)
C(67)-C(69)-C(70)-C(71)	-0.6(19)
C(68)-N(16)-C(71)-C(70)	0.3(15)
C(68)-N(16)-C(71)-C(72)	-179.5(8)
C(68)-C(67)-C(69)-C(70)	0.8(18)
C(69)-C(67)-C(68)-N(16)	-0.5(15)
C(69)-C(70)-C(71)-N(16)	0.0(18)
C(69)-C(70)-C(71)-C(72)	179.8(12)
C(70)-C(71)-C(72)-N(13)	-179.1(11)
C(70)-C(71)-C(72)-C(73)	0(2)
C(71)-N(16)-C(68)-C(67)	0.0(13)
C(72)-N(13)-N(14)-N(15)	-1.5(10)
C(73)-N(15)-C(74)-C(75)	-81.6(15)
C(74)-N(15)-C(73)-C(72)	178.8(11)
C(74)-C(75)-C(76)-C(77)	-174.5(12)
C(74)-C(75)-C(80)-C(79)	177.4(14)
C(74)-C(75)-C(81)-C(84)	-179.9(13)
C(75)-C(76)-C(77)-C(78)	60.4(18)
C(75)-C(76)-C(77)-C(82)	-63(2)
C(75)-C(81)-C(84)-C(82)	54(2)
C(75)-C(81)-C(84)-C(83)	-59.3(18)

C(76)-C(75)-C(80)-C(79)	58.5(18)
C(76)-C(75)-C(81)-C(84)	-57.7(15)
C(76)-C(77)-C(78)-C(79)	-62(2)
C(76)-C(77)-C(82)-C(84)	61(2)
C(77)-C(78)-C(79)-C(80)	61(2)
C(77)-C(78)-C(79)-C(83)	-60.5(18)
C(77)-C(82)-C(84)-C(81)	-54(2)
C(77)-C(82)-C(84)-C(83)	59.1(19)
C(78)-C(77)-C(82)-C(84)	-61(2)
C(78)-C(79)-C(80)-C(75)	-60(2)
C(78)-C(79)-C(83)-C(84)	62(2)
C(79)-C(83)-C(84)-C(81)	55(2)
C(79)-C(83)-C(84)-C(82)	-60(2)
C(80)-C(75)-C(76)-C(77)	-58.0(17)
C(80)-C(75)-C(81)-C(84)	61.3(18)
C(80)-C(79)-C(83)-C(84)	-58(2)
C(81)-C(75)-C(76)-C(77)	61.1(15)
C(81)-C(75)-C(80)-C(79)	-59.3(19)
C(82)-C(77)-C(78)-C(79)	60(2)
C(83)-C(79)-C(80)-C(75)	59(2)

Symmetry transformations used to generate equivalent atoms:

#1 -x+y+1,-x+1,z #2 -y+1,x-y,z

10. References

1. T. Schröder, M. Gartner, T. Grab, S Bräse, *Org. Biomol. Chem.* **2007**, *5*, 2767.

2. B. H. Lipshutz, B. R. Taft, *Angew. Chem. Int. Ed.* **2006**, *45*, 8235.

3. N. R. Voss, M. Gerstein, *Nucleic Acids Res.* **2010**, *38*, W555.

4. S. Mecozzi, J. Rebek, *Chem. Eur. J.* **1998**, *4*, 1016.

5. D. Zuccaccia, L. Pirondini, R. Pinalli, E. Dalcanale, A. Macchioni, *J. Am. Chem. Soc.* **2005**, *127*, 7025.

6. Y. R. Hristova, M. M. J. Smulders, J. K.Clegg, B. Breiner, J. R. Nitschke, *Chem. Sci.* **2011**, *2*, 638.

7. Data integration and absorption correction: Agilent Technologies, **2013**, *CrysAlisPro*, Agilent Technologies UK Ltd, Yarnton, UK.

8. Structure solution, refinement and graphics (SHELXTL): G. M. Sheldrick , *Acta Cryst.* **2008**, *A64*, 112–122.

9. For example, see I. S. Tidmarsh, T. B. Faust, H. Adams, L. P. Harding, L. Russo, W. Clegg, M. D. Ward, *J. Am. Chem. Soc.* **2008**, *130*, 15167.

10. Graphics and solvent void analysis (OLEX2): O.V. Dolomanov, L.J.Bourhis, R.J. Gildea, J.A.K. Howard, H. Puschmann, *J. Appl. Cryst.*, **2009**, *42*, 339.
